# The global prevalence of hepatitis D virus infection: Systematic review and meta-analysis

**DOI:** 10.1016/j.jhep.2020.04.008

**Published:** 2020-09

**Authors:** Alexander J. Stockdale, Benno Kreuels, Marc Y.R. Henrion, Emanuele Giorgi, Irene Kyomuhangi, Catherine de Martel, Yvan Hutin, Anna Maria Geretti

**Affiliations:** 1 Institute of Infection and Global Health, University of Liverpool, Liverpool, United Kingdom; 2 Malawi-Liverpool-Wellcome Trust Clinical Research Programme, Blantyre, Malawi; 3 College of Medicine, Blantyre, Malawi; 4 University Medical Centre Hamburg-Eppendorf, Hamburg, Germany; 5 Liverpool School of Tropical Medicine, Liverpool, United Kingdom; 6 Centre for Health Informatics, Computing, and Statistics, University of Lancaster, Lancaster, United Kingdom; 7 International Agency for Research on Cancer, Lyon, France; 8 World Health Organization, Geneva, Switzerland

**Keywords:** Hepatitis D, Hepatitis delta virus, Hepatitis B, Liver cirrhosis, Carcinoma, Hepatocellular, Prevalence, Epidemiology, Meta-analysis

## Abstract

**Background and Aims:**

There are uncertainties about the epidemic patterns of HDV infection and its contribution to the burden of liver disease. We estimated the global prevalence of HDV infection and explored its contribution to the development of cirrhosis and hepatocellular carcinoma (HCC) among HBsAg-positive people.

**Methods:**

We searched Pubmed, EMBASE and Scopus for studies reporting on total or IgG anti-HDV among HBsAg-positive people. Anti-HDV prevalence was estimated using a binomial mixed model, weighting for study quality and population size. The population attributable fraction (PAF) of HDV to cirrhosis and HCC among HBsAg-positive people was estimated using random effects models.

**Results:**

We included 282 studies, comprising 376 population samples from 95 countries, which together tested 120,293 HBsAg-positive people for anti-HDV. The estimated anti-HDV prevalence was 4.5% (95% CI 3.6–5.7) among all HBsAg-positive people and 16.4% (14.6–18.6) among those attending hepatology clinics. Worldwide, 0.16% (0.11–0.25) of the general population, totalling 12.0 (8.7–18.7) million people, were estimated to be anti-HDV positive. Prevalence among HBsAg-positive people was highest in Mongolia, the Republic of Moldova and countries in Western and Middle Africa, and was higher in injecting drug users, haemodialysis recipients, men who have sex with men, commercial sex workers, and those with HCV or HIV. Among HBsAg-positive people, preliminary PAF estimates of HDV were 18% (10–26) for cirrhosis and 20% (8–33) for HCC.

**Conclusions:**

An estimated 12 million people worldwide have experienced HDV infection, with higher prevalence in certain geographic areas and populations. HDV is a significant contributor to HBV-associated liver disease. More quality data are needed to improve the precision of burden estimates.

**Lay summary:**

We combined all available studies to estimate how many people with hepatitis B also have hepatitis D, a viral infection that only affects people with hepatitis B. About 1 in 22 people with hepatitis B also have hepatitis D, increasing to 1 in 6 when considering people with liver disease. Hepatitis D may cause about 1 in 6 of the cases of cirrhosis and 1 in 5 of the cases of liver cancer that occur in people with hepatitis B. Hepatitis D is an important contributor to the global burden of liver disease.

## Highlights

• HDV infection is common among HBsAg-positive people worldwide.
• Among HBsAg-positive people, estimated HDV prevalence is 4.5% (95% CI 3.6–5.7).
• HDV prevalence in HBsAg-positive hepatology clinic attendees is 16.4% (14.6–18.6).
• HDV prevalence is higher in people who inject drugs and who have HCV or HIV.
• HDV causes an estimated 18% of cirrhosis and 20% of HCC associated with hepatitis B.

## Graphical abstract

**Figure:**
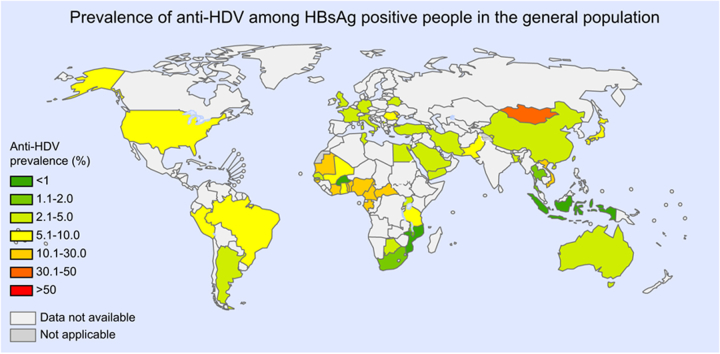

See Editorial, pages 493–495

## Introduction

Globally, chronic infection with the HBV is an important cause of liver-related morbidity and mortality due to its widespread distribution. An estimated 257 to 291 million people are chronically infected with HBV and are at risk of cirrhosis and hepatocellular carcinoma (HCC).^1^^,^^2^ The proportion of cirrhosis attributable to HBV varies geographically according to HBV epidemiology, ranging from 6% in North America, to 6-21% in Latin America, 34–38% in sub-Saharan Africa, and 39% in East Asia.^3^ Worldwide, HBV is associated with a third of deaths from HCC.^4^

HDV is a satellite RNA virus that depends on HBV for propagation.^5^ It uses the HBsAg as a viral envelope and shares the same hepatocyte receptor for viral entry.^6^ HDV is among the smallest of viruses capable of causing human disease, yet HBV co-infection with HDV is the most severe form of viral hepatitis.^5^ HDV transmission follows 2 patterns. Infection occurring simultaneously with HBV can cause extensive hepatic necrosis and manifest as a severe or even fulminant hepatitis with a high case fatality rate.^6^ With recovery, simultaneous infection in adults usually results in clearance of both viruses.^5^ Super-infection of persons with chronic HBV infection typically results in HDV persistence, leading to accelerated progression to cirrhosis and an increased risk of HCC relative to chronic HBV infection alone.^7^ Due to variation in awareness and incomplete testing among HBsAg-positive people,^8^ issues with standardisation of confirmatory molecular diagnostic techniques,^9^ and a historical lack of effective treatment options,^10^ HDV ascertainment has been suboptimal, even in high-income settings. HDV may therefore have an under-recognised role in the causation of liver disease and liver-related deaths.

Estimating HDV prevalence and the relative contribution of HDV towards liver disease, including among general populations and specific population groups, is critical to guide clinical care and policy formulation and inform effective public health interventions and development of new medicines. Yet, obtaining accurate estimates of HDV epidemiology is challenging for several reasons. Firstly, at the population level, large sample sizes are required to identify HBsAg-positive individuals prior to testing for HDV. In settings with low prevalence of HBV infection, sufficiently large surveys may not be feasible. Secondly, heterogeneity in HDV estimates might be expected because of variable and potentially evolving epidemic patterns, as well as variations in methodology. Thirdly, the selection criteria for HBsAg and subsequent HDV testing may lead to non-representative sampling. Therefore, careful assessment of potential biases and assessment of representativeness is required to synthesise epidemiological estimates of HDV prevalence.

In a previous review, we identified foci of HDV endemicity in sub-Saharan Africa.^11^ This systematic review and meta-analysis aimed to identify and collate all globally available epidemiological data on HDV infection from January 1998 to January 2019. We aimed to describe the geographic distribution of HDV infection and to produce regional estimates of HDV infection among general populations, individuals attending hepatology clinics, and selected population groups, being mindful of potential sources of error. We further aimed to estimate the proportion of HBV-associated cirrhosis and HCC attributable to HDV.

## Materials and methods

### Outcomes of the analysis

We performed a systematic review and meta-analysis of HDV prevalence in the 6 World Health Organisation (WHO) regions. The primary outcome was the prevalence of total or IgG anti-HDV among HBsAg-positive people. The prevalence of HDV RNA detection was estimated among individuals positive for HBsAg and anti-HDV based on assays employing PCR. The secondary outcome was the estimation of the population attributable fraction (PAF) of HDV among HBsAg-positive persons with cirrhosis and HCC. We conducted the study in accordance with the principles of the PRISMA and GATHER statements, and registered it with PROSPERO (CRD42018113039).

### Search method

We searched PubMed, EMBASE and Scopus on 17^th^ April 2018 for articles published since 1 January 1998 and repeated the search on 28 January 2019. The search strategy included synonyms of hepatitis D and terms describing HDV epidemiology and diagnosis (detailed in supplementary appendix 1). We included all languages. Articles in 8 languages unknown to the study team were translated into English. Between 18^th^ and 21^st^ February 2019, we also performed searches of the grey literature within the Global Health Data Exchange database, international health surveillance programmes, and official national health surveillance websites (listed in supplementary appendix 2), and reviewed surveys, censuses, vital statistics, and reports not already included in previously identified scientific publications. We contacted 21 corresponding authors to seek clarifications on their published data. We additionally searched GenBank (https://www.ncbi.nlm.nih.gov/genbank/) and the European Nucleotide Archive (http://www.ebi.ac.uk/ena) to retrieve HDV sequences and genotypes.

### Inclusion and exclusion criteria

We included studies that described the geographic and clinical setting of participants with HBsAg and applied a systematic selection method to anti-HDV testing, whereby all eligible consenting participants, or a randomly selected representative sample were tested. Duplicate data, studies that commenced before 1988, and studies with evidence of selective or non-systematic testing were excluded. Studies were also excluded if they used anti-HDV IgM, hepatitis D antigen (HDAg) or HDV RNA as an initial test as these are inconsistently expressed markers of HDV infection.^12^ Studies that focussed on the following groups were excluded: individuals with acute hepatitis, returning (repeat) or remunerated blood donors, migrant populations whose data could be unrepresentative of either the country of origin or the country of destination, children <18 months who may have maternal anti-HDV transfer, and liver transplant registers or recipients, due to the variability of selection criteria. Included studies are referenced in supplementary appendices 3–6.

### Data extraction and assessment of study quality

Following removal of duplicate articles, 2 authors (AJS and BK) independently screened potentially relevant articles to determine suitability for inclusion. We extracted data comprising year of sampling, geographic representation of the sample (for example, a catchment area of a clinic or the geographic boundaries of a community study), population type, clinical setting, testing method and assay manufacturer, anti-HDV and HDV RNA prevalence, and HDV genotype data. We derived a data quality score from an appraisal checklist (detailed in supplementary appendix 7) customized from 2 previously published quality assessment tools for prevalence studies.^13^^,^^14^ The assessment was based on 3 main criteria relating to i) adequacy of description of inclusion and exclusion criteria; ii) recruitment methodology; and iii) assessment of risk of bias. Determination of eligibility for inclusion, data extraction and risk of bias were assessed independently (by AJS and BK) with disagreement resolved by consensus.

### Statistical methods

Anti-HDV prevalence was described among 3 groups: i) general populations, comprising people tested in community surveys, antenatal clinics, or occupational settings, students, and blood donors (unless repeat or remunerated); ii) hepatology clinic populations, comprising patients reviewed in a hepatology service, regardless of disease status; and iii) selected population groups, comprising people who inject drugs (PWID), haemodialysis recipients, men who have sex with men (MSM), commercial sex workers (CSW), and people with HCV or HIV. In sub-Saharan African countries with generalised HIV epidemics (adult prevalence >1% based on UNAIDS estimates, https://aidsinfo.unaids.org/) people with HIV were included in the general population (note: we previously documented a lack of association between HIV status and anti-HDV prevalence in this setting).^11^ The definition of general populations excluded isolated or remote communities to avoid biasing the general estimates.

Anti-HDV prevalence among HBsAg-positive people within general populations and hepatology clinic populations was modelled using a binomial mixed model (detailed in supplementary appendix 8). The estimates were weighted by the data quality score described above (supplementary appendix 7) and the size of the represented population. For each population, the size of the catchment area was estimated from the latest available census data (detailed in supplementary appendix 9).^15^ For nationally representative surveys, United Nations Population Division population estimates for 2018 were used.^16^ To predict anti-HDV prevalence in the overall population (without conditioning on HBsAg status), we used WHO HBsAg prevalence estimates for general populations.^17^ The 95% CIs for the number of people with anti-HDV and HDV RNA was estimated using a parametric bootstrap procedure (supplementary appendix 8). Heterogeneity across populations and between samples (individual data points) was accounted for by the introduction of an explicit covariate and by the random effects model (supplementary appendix 8).

To evaluate the association between anti-HDV prevalence and selected population groups, odds ratios (ORs) were pooled using a random effects model. For studies that did not report a comparable control population, the comparator was drawn from the highest quality available dataset that had been obtained from either the general population or asymptomatic HBsAg-positive people in the same geographic region. Asymptomatic HBsAg-positive people were defined as patients who had been clinically evaluated and found not to have evidence of active chronic hepatitis, cirrhosis or HCC. In the analysis of HIV-positive people, comparator populations were HIV-negative populations or general populations from the same geographic region. Subgroup estimates, stratified by the type of control population used, were produced to examine for potential sources of heterogeneity. We calculated I^2^ and τ^2^ to estimate heterogeneity where I^2^ represents the proportion of variability that could be attributed to heterogeneity and not sampling error and τ^2^ represents an estimate of between-study variance, for each group.

We estimated prevalence of HDV RNA among people with anti-HDV using a random effects model and analysed associations between study characteristics and rates of HDV RNA detection using random effects residual maximum likelihood aggregate level meta-regression with Knapp and Hartung adjustment, using the package *metareg* in Stata. Coefficients were calculated to indicate the change in proportion of HDV RNA per unit change in explanatory variable, where for example, a coefficient of 0.1 indicates a 10% increase in HDV RNA detection per unit increase in the explanatory variable.

To facilitate interpretation of anti-HDV prevalence estimates among patients diagnosed with cirrhosis or HCC, we calculated the PAF of HDV for cirrhosis and HCC among HBsAg-positive people. We used methodology recommended by the WHO, where PAF = prevalence among cases × [(odds ratio − 1)/odds ratio].^18^^,^^19^ where OR is used as an approximation for the disease risk ratio of exposed (anti-HDV positive) *vs.* non-exposed (anti-HDV negative) participants. The OR calculation included HBsAg-positive general populations or asymptomatic HBsAg-positive people (as defined above), using empirical data from the highest quality and largest sample available from the same geographic region as the control population.^3^^,^^4^^,^^20^

We used a parametric bootstrap method to estimate confidence intervals for the PAF for each study, and simulated anti-HDV prevalence in cases and controls using a binomial distribution. We estimated pooled PAF using a DerSimonian-Laird random effects model, with a Knapp-Sidik-Jonkman adjustment as implemented in the R package *meta*.[21], [22], [23]

Analyses were performed in ArcGIS Pro 2.0 (ESRI, Redland, CA, USA), Stata 16.0 (College Station, TX, USA) and R v3.6.0 (R Foundation for Statistical Computing, Vienna).^23^

For further details regarding the materials and methods used, please refer to the CTAT table and supplementary information.

## Results

### Searches

**Fig. 1:**
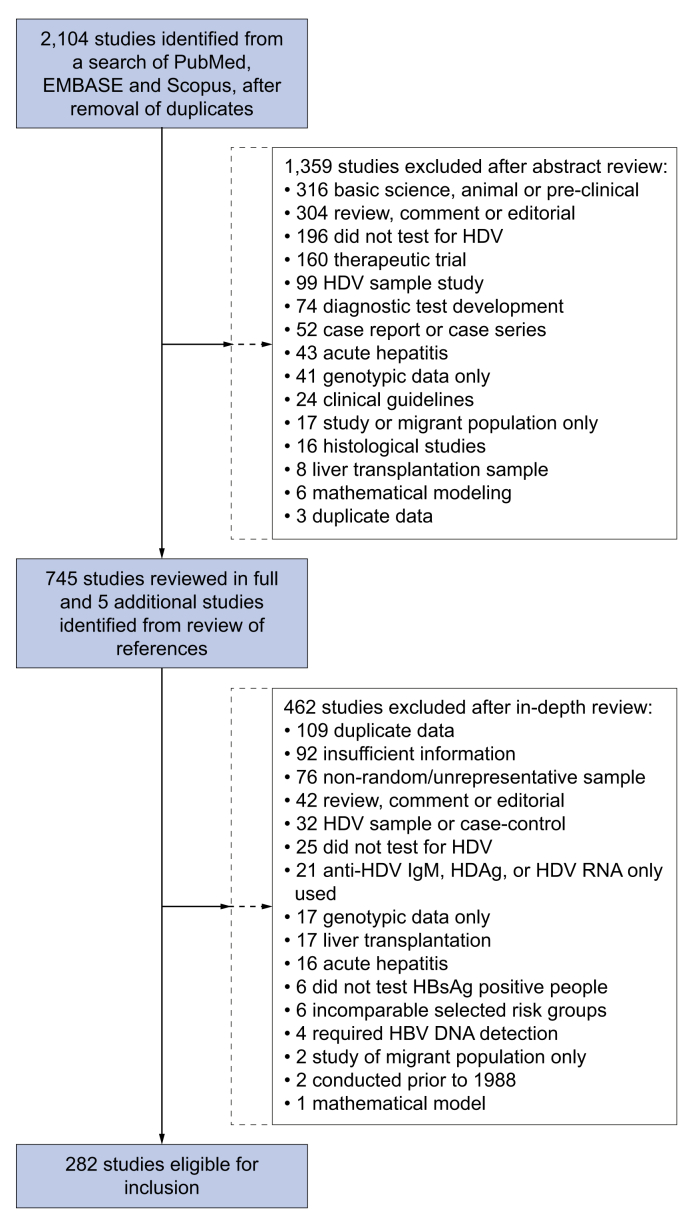
Selection of studies for inclusion in systematic review of global HDV epidemiology.

A search of PubMed, Embase, and Scopus identified 2,104 articles published between 1 January 1998 and 28^th^ January 2019, after removal of duplicate citations. Following a review of abstracts, we selected 745 articles for review of full text (Fig. 1). We identified 329 reports for review in the grey literature, and none was eligible for inclusion, mainly due to selection bias or incomplete reporting. We included 282 studies, reporting data from 376 unique population samples from 95 countries.

### Data availability

**Fig. 2:**
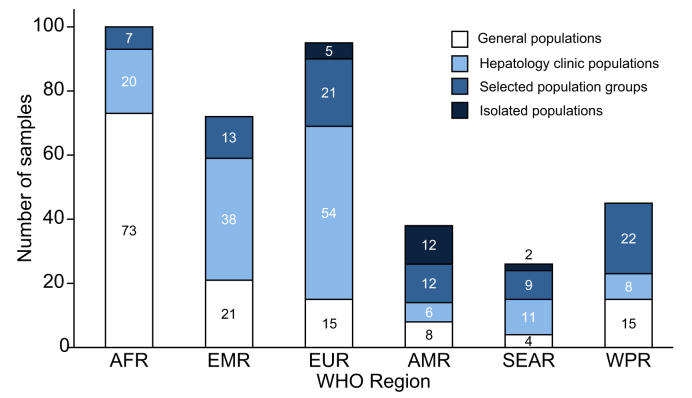
Distribution of included samples by population category and WHO region^a^. ^a^Selected population groups comprised people who inject drugs, haemodialysis recipients, men who have sex with men, commercial sex workers, and people with hepatitis C virus or HIV. In sub-Saharan Africa countries with adult HIV prevalence >1%, HIV populations were included in the general population. Isolated populations were selected sample populations that were considered not representative of the general population. WHO, World Health Organisation; AFR, African Region; EMR, Eastern Mediterranean Region; EUR, European Region; AMR, Region of the Americas; SEAR, South-East Asian Region; WPR, Western Pacific Region.

**Fig. 3:**
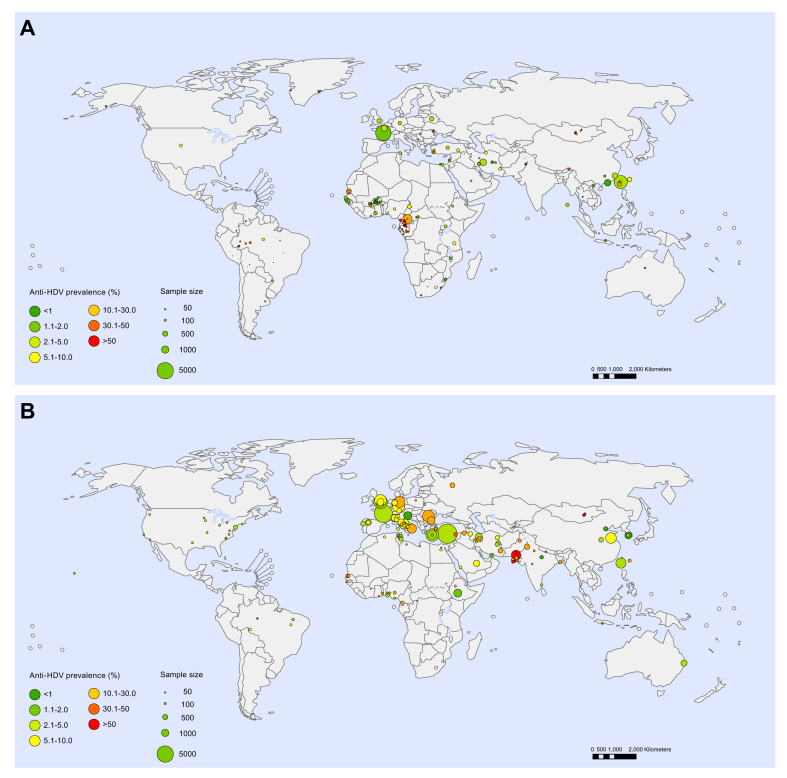
Anti-HDV prevalence in HBsAg-positive people. (A) General populations; (B) Hepatology clinic populations. Each point represents a sample. Point size indicates sample size and colour indicates HDV seroprevalence.

Overall, across 6 WHO regions, 155 samples were from general populations, 137 were from hepatology clinics, and 84 were from selected population groups (Fig. 2, supplementary appendix 10). We excluded from general population estimates 19 studies that sampled isolated island or remote indigenous communities (supplementary appendix 11). Repeated longitudinal testing of the same population was reported from 12 samples. Overall, 120,293 HBsAg-positive individuals were tested for anti-HDV using various assays (supplementary appendix 12); in 12.7% of the studies, the anti-HDV testing method was not specified. Overall, 5,065 anti-HDV positive participants were tested for HDV RNA (Fig. 3).

### Anti-HDV prevalence in general populations and hepatology clinic populations

**Fig. 4:**
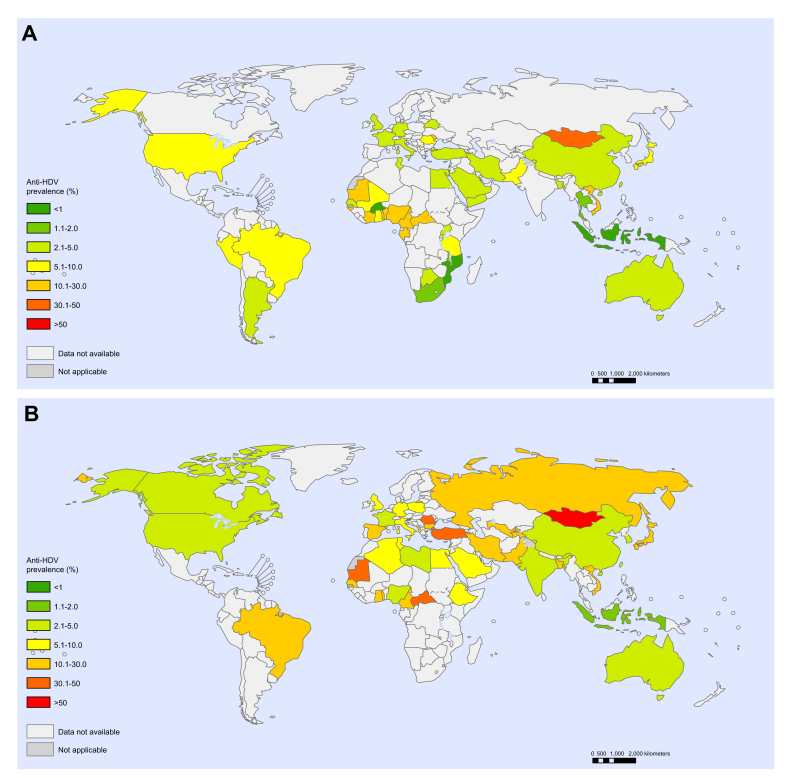
Country-level estimates of anti-HDV prevalence among HBsAg-positive people. (A) General populations; (B) Hepatology clinic populations. Colour indicates HDV seroprevalence.

In the general population, the global estimated anti-HDV prevalence was 4.5% (95% CI 3.6–5.7) among HBsAg-positive people and 0.16% (95% CI 0.11–0.25) overall, with regional estimates for HBsAg-positive people ranging from 3.0% in Europe to 6.0% in Africa (Tables 1 and 2). This represents an estimated 12.0 million HDV seropositive individuals globally (95% CI 8.7–18.7). In hepatology clinic populations, the global estimated anti-HDV prevalence was 16.4% (95% CI 14.6–18.6) among HBsAg-positive people, with estimates ranging from 3.3% in the Americas to 19.5% in Europe (Table 1). Country-level estimates for general populations and hepatology clinic populations are shown in Fig. 4 and are detailed in supplementary appendix 13. Among HBsAg-positive people, Mongolia had the highest national anti-HDV prevalence (36.9%); prevalence rates >10% were also estimated for the Republic of Moldova and countries in Western and Middle Africa. Several isolated communities were noted to have high HDV anti-prevalence, including indigenous Amazonian Amerindian tribes in Bolivia, Brazil, Colombia and Venezuela, indigenous tribes in the Uttar Pradesh region of India, and selected populations in Greenland and Rhodes (Greece) (Fig. 3, supplementary appendix 11). Only limited data were available for North America, Latin America and Southern Africa and, particularly in view of the size of the populations represented, there were small samples available for the Western Pacific and South-East Asia regions relative to other WHO regions (Figs. 2 and 3).

**Table 1:** Estimated anti-HDV prevalence in general and hepatology clinic HBsAg-positive populations, by WHO region.

WHO region	HBsAg-positive populations			
	General		Hepatology clinics	
	%	(95% CI)	%	(95% CI)
AFR	5.97	(4.98–7.24)	12.26	(10.13–14.70)
AMR	5.91	(3.02–9.71)	3.34	(2.58–4.21)
EMR	3.54	(2.10–6.28)	17.36	(11.15–26.34)
EUR	3.00	(2.09–4.21)	19.48	(17.31–21.76)
SEAR	3.20	(0.36–12.4)	4.00	(3.09–5.15)
WPR	4.09	(3.47–4.77)	8.07	(7.50–8.64)
Global	4.49	(3.57–5.68)	16.42	(14.58–18.56)

AFR, African Region; AMR, Region of the Americas; EMR, Eastern Mediterranean Region; EUR, European Region; SEAR, South-East Asian Region; WHO, World Health Organisation; WPR, Western Pacific Region.

**Table 2:** Estimates of HDV prevalence in the general population, by WHO region^a^.

	AFR	AMR	EMR	EUR	SEAR	WPR	Global
Population (thousands)	1,052,766	1,006,458	715,425	928,490	1,982,239	1,945,717	7,631,091
HBsAg prevalence, % (95% CI)	6.1 (4.6–8.5)	0.7 (0.4–1.6)	3.3 (2.6–4.3)	1.6 (1.2–2.6)	2.0 (1.5–4.0)	6.2 (5.1–7.6)	3.5 (2.7–5.0)
Anti-HDV prevalence among people with HBsAg, % (95% CI)	6.0 (5.0–7.2)	5.9 (3.0–9.7)	3.5 (2.1–6.3)	3.0 (2.1–4.2)	3.2 (0.4–12.4)	4.1 (3.5–4.8)	4.5 (3.6–5.7)
Anti-HDV prevalence among the general population, % (95% CI)	0.36 (0.26–0.54)	0.04 (0.02–0.11)	0.12 (0.07–0.23)	0.05 (0.03–0.09)	0.06 (0.01–0.35)	0.25 (0.20–0.33)	0.16 (0.11–0.25)
HDV RNA prevalence among people with anti-HDV, % (95% CI)	41.3 (31.8–51.1)	64.2 (21.5–98.0)	49.4 (30.1–68.7)	64.1 (54.3–73.3)	50.1 (31.4–70.3)	73.3 (57.8–68.7)	58.5 (52.4–64.5)
HDV RNA prevalence among the general population, % (95% CI)	0.15 (0.10–0.24)	0.03 (0.01–0.09)	0.06 (0.03–0.12)	0.03 (0.02–0.06)	0.03 (0.00–0.18)	0.19 (0.13–0.26)	0.09 (0.07–0.15)
Number of people with anti-HDV, thousands, % (95% CI)	3,835 (2,779–5,706)	416 (185–1,135)	836 (482–1,610)	445 (293–833)	1,267 (172–6,841)	4,935 (3,836–6,391)	11,992 (8,662–18,743)
Number of people with HDV RNA, thousands, (95% CI)	1,584 (1,059–2,506)	267 (78–881)	413 (203–877)	285 (184–544)	635 (83–3,622)	3,617 (2,583–4,971)	7,015 (4,994–11,109)

AFR, African Region; AMR, Region of the Americas; EMR, Eastern Mediterranean Region; EUR, European Region; SEAR, South-East Asian Region; WHO, World Health Organisation; WPR, Western Pacific Region.

a Sources: Population data for 2018 from UN Population Division World Population Prospects 2019^16^; HBV prevalence estimates from WHO Global Hepatitis Report 2017.^1^

### Anti-HDV prevalence in selected population groups

**Fig. 6:**
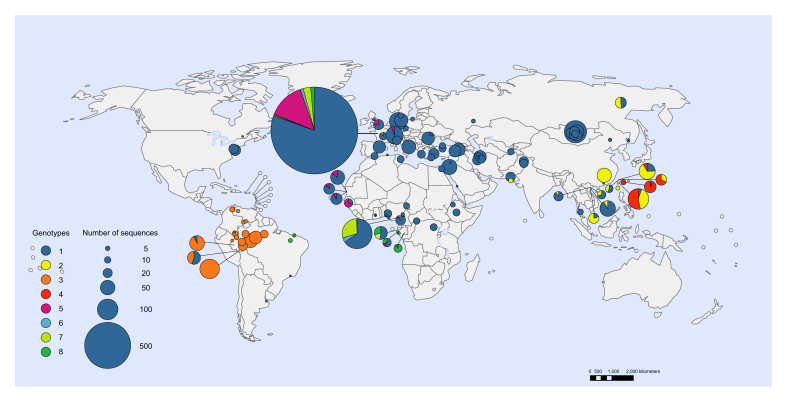
Geographic distribution of HDV genotypes^a^. ^a^Each circle represents a unique sample with area proportional to sample size aGenotype data identified by searches and from publicly available HDV sequences deposited in GenBank (https://www.ncbi.nlm.nih.gov/genbank/) and European Nucleotide Archive Database (http://www.ebi.ac.uk/ena).

The odds of anti-HDV detection were analysed in 6 selected populations relative to general populations or asymptomatic HBsAg-positive people from the same geographic region (Fig. 6 and supplementary appendices 14–20). Anti-HDV prevalence was higher in PWID (pooled OR 19.0) and in haemodialysis recipients (pooled OR 3.4). Five studies from China and Vietnam indicated that CSWs also had higher prevalence (pooled OR 18.7). Three studies from Italy, Burkina Faso and Indonesia investigated anti-HDV prevalence among MSM. No cases were reported in the Burkina Faso study, whereas data from the other 2 studies indicated that anti-HDV prevalence was higher among MSM (pooled OR 16.0). The odds of anti-HDV detection were also higher among anti-HCV positive people (pooled OR 10.0) and among HIV-positive people in countries without generalised HIV epidemics (pooled OR 6.6). In countries with generalised HIV epidemics, the odds of anti-HDV detection were similar in HIV-positive and HIV-negative people (pooled OR 0.8).

### HDV RNA detection and HDV genotypes

HDV RNA was tested in 5,073 anti-HDV positive people. The pooled proportion with HDV RNA detection was 58.5% (95% CI 52.4–64.5) (supplementary appendix 21). Overall, HDV RNA prevalence correlated with anti-HDV prevalence, with a coefficient of 0.03 (95% CI 0.02–0.06; *p* <0.0001) indicating a 3% increase in HDV RNA detection per each 10% increase in anti-HDV prevalence. HDV RNA detection was lower in general populations relative to hepatology clinic populations (coefficient −0.20; 95% CI −0.33 to −0.07; *p* = 0.003). There was also lower HDV RNA detection in the African region compared to other WHO regions (41.3% *vs.* 64.9%; coefficient −0.13; 95% CI −0.26 to −0.002; *p* = 0.05). Subject to the limitations of the available data, a preliminary estimate of 7.1 million people (95% CI 5.0–11.1) had viraemic infection (HDV RNA positive), representing a general population prevalence of viraemia of 0.09% (95% CI 0.07–0.15).

Searches of studies reporting HDV genotype data identified 4,159 individuals with available results (supplementary appendices 22 and 23). Genotype 1 predominated globally (89.9% of published data). Other genotypes were more localised, including genotype 2 in Asia, genotype 3 in Latin America (predominating in the Amazon basin), genotype 4 in Japan and China (Taiwan), genotype 5 in Western Africa, and genotypes 6–8 in Middle Africa (Fig. 6).

### Population attributable fraction for cirrhosis and HCC among HBsAg-positive people

**Fig. 5:**
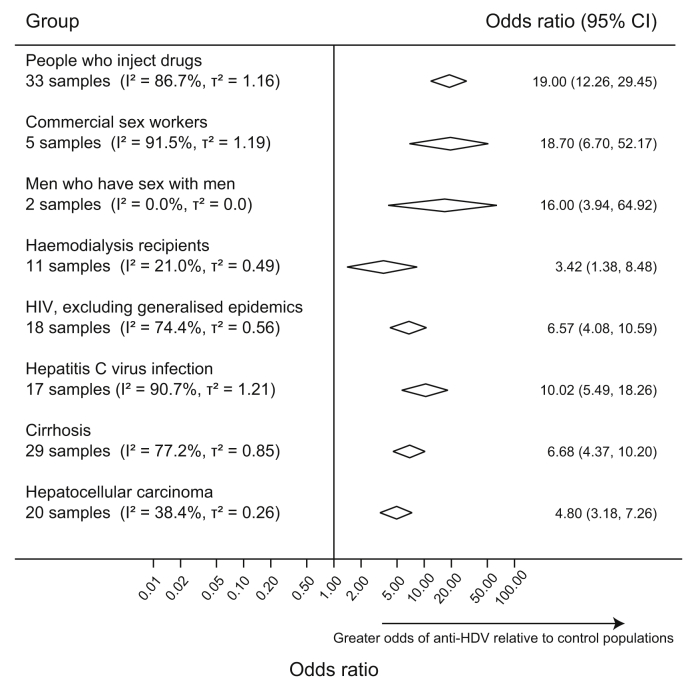
HDV seroprevalence among selected population groups relative to general populations or asymptomatic HBsAg-positive people from the same geographic region^a^. ^a^Comparators are general population or asymptomatic HBsAg-positive samples from the same geographic region. Odds ratios were pooled using a random effects model. Diamonds indicate central estimate and 95% CI for pooled odds ratios for each population group. I^2^ represents the proportion of variability that can be attributed to heterogeneity. τ^2^ represents an estimate of between-study variance, for each group.

Patients with cirrhosis or HCC had higher anti-HDV prevalence relative to comparator populations from the same geographic region, with pooled ORs of 6.7 and 4.8, respectively (Fig. 5 and supplementary appendices 24 and 25). Among HBsAg-positive people with sequelae of infection, our PAF calculation suggested that HDV accounted for 18% of cirrhosis (95% CI 10–26) and 20% of HCC (95% CI 8–33) (supplementary appendices 26 and 27).

## Discussion

There has been uncertainty about the epidemic patterns of HDV infection, at least partly reflecting the challenges in obtaining representative estimates of anti-HDV prevalence.^24^ To address the issue, in this systematic review, we took into account population sizes and representativeness and considered the risk of bias of prevalence studies. Globally, we estimate an anti-HDV prevalence of 4.5% among HBsAg-positive people, which translates into an estimated prevalence of 0.16% in the total population. This represents an estimated 12 million people with serological evidence of HDV infection globally. The geographic distribution of HDV infection is heterogeneous, with particularly high prevalence reported in Mongolia, the Republic of Moldova, and countries in Western and Middle Africa. We also identified an epidemiological association between anti-HDV prevalence and several population groups including PWID, recipients of haemodialysis, CSWs and MSM, and between anti-HDV prevalence and HCV or HIV infection, which may be secondary to shared transmission routes. These data are useful to set priorities about HDV testing. Interventions that prevent HBV and HCV infection should also be effective in preventing HDV, including immunization against hepatitis B and harm reduction strategies in PWID.[25], [26], [27]

There remain limitations to the epidemiological data available to inform this analysis. There are significant data gaps, most strikingly in North America and Latin America, Southern Africa and most of Asia, where more data are required to obtain accurate estimates of anti-HDV prevalence. These gaps should be addressed through future representative epidemiological studies with efforts to elucidate population groups at increased prevalence of infection. There was heterogeneity in anti-HDV prevalence estimates even within closely related geographic areas, which is reflected in the confidence intervals for the estimates. Heterogeneity suggested likely variation in local risk factors and the occurrence of localised, geographically defined foci of transmission. Sources of heterogeneity also included methodological issues, such as variation in study design, inclusion and exclusion criteria and sampling frameworks. It should be noted that estimates in our study consider individuals living in regions with limited or no data for anti-HDV prevalence. To do so we calculated the number of people with anti-HDV in each WHO region, by multiplying the anti-HDV prevalence estimates for each region (based on available data) with the number of people in the general population estimated to be HBsAg positive (based on WHO HBsAg regional estimates). Collection of additional data to inform epidemiological estimates for regions with limited data represents a research priority.

Unbiased ascertainment of anti-HDV prevalence requires large sample sizes to identify the subset with HBV, which poses a particular problem in regions with low HBV prevalence. In these regions, samples derived from settings that are enriched with HBsAg-positive people are often used to study anti-HDV prevalence, such as patients sampled within hepatology clinics. Current guidelines recommend HDV testing in selected patients with specific risk factors^28^ or do not explicitly recommend universal testing.^29^ As a result, in many centres, newly diagnosed HBsAg-positive individuals are not tested for anti-HDV as part of routine practice. Clinician-driven testing carries a risk of introducing an ascertainment bias. Specialist referral to a hepatology clinic may be the result of patients developing characteristics of liver disease. The resulting population may therefore be more likely to test anti-HDV positive and may not be fully representative of the general population. As such, to reduce the risk of overestimating HDV prevalence, studies based in hepatology clinic settings were considered separately from those of general populations. Furthermore, we only included samples that were representative of the target population and decided to exclude many laboratory-based samples or samples that relied on clinician-instigated testing. A previous systematic review estimated a global anti-HDV prevalence among people with HBsAg of 10.6%.^24^ However, as we previously noted, the analysis included data from sources at high risk of bias such as laboratory-based studies, analysed hepatology clinic data together with data from general populations, did not consider the size of the population represented by the included samples, and used data from small isolated communities to produce estimates for general populations.^24^^,^^25^ Our study has considered each of these issues and as a result we estimate a lower anti-HDV prevalence among the general population and provide a distinct estimate for hepatology clinic populations.

This study did not aim to address temporal trends in anti-HDV prevalence, as only a small number of studies reported repeated longitudinal data. We also found very few studies including children. It is anticipated anti-HDV prevalence may be low in children, due to the absence of specific risk factors and a short duration of risk exposure. Global estimates for HBV prevalence are significantly lower among children, largely as a result of HBV vaccination, estimated at 1.3% in children under 5 years, relative to 3.5% among the general population globally.^1^ Accordingly, HDV infection is expected to be uncommon in children and to decline over time due to increasing implementation of HBV vaccination.

Due to the limitations of the available data, alongside concerns about comparability and standardisation of HDV RNA assays,^9^ prevalence estimates in this study were based primarily on detection of anti-HDV. We also provided a provisional estimate of viraemic, HDV RNA-positive individuals. A limited number of studies used HDV RNA detection for confirmation of a current infection in participants with anti-HDV; in these studies, the pooled estimate for HDV RNA positivity was 58.5%. It is important to note that relative to the data available to inform anti-HDV estimates, the data for HDV RNA represent a significantly smaller sample size with limited representation from some regions and the estimate of viraemic infections should therefore be considered provisional. HDV RNA detection was lower in general populations relative to hepatology clinic populations, and positively correlated with anti-HDV prevalence. This may reflect the higher pre-test probability of a current HDV infection in HBsAg-positive people who underwent HDV testing in hepatology clinics relative to general populations. HDV RNA detection was also less common in the Africa region relative to other regions, but this observation should be interpreted with caution: HDV RNA assays have had historical performance issues and particular difficulties with the detection of African genotypes 5–8.^9^ With concerted efforts to improve and standardise HDV RNA detection methods, epidemiological data based on HDV RNA detection may become more informative in the future and also help to ascertain the relationship between viraemia and clinical outcomes.

Furthermore, anti-HDV assays used in the included studies were from a diverse range of manufacturers. Comparative data on the diagnostic performance characteristics of anti-HDV tests are lacking and would be informative. Limited data suggests a potential for false negative anti-HDV ELISA test results, at least when comparing one commercial anti-HDV assay with a novel anti-HDV microarray test in a hyperendemic setting in Mongolia.^26^ This initial finding requires verification in other regions and populations, with diverse HDV genotypes, and across the different anti-HDV assays available commercially to determine whether anti-HDV underestimation is a broader problem.

Estimations of the global burden of disease from viral hepatitis have not previously considered HDV,^30^ and HDV epidemiology and disease control are not considered in the current Global Health Sector Strategy for Viral Hepatitis.^27^ We estimated that, globally, between 1 in 5, to 1 in 6 cases of cirrhosis or HCC among people with hepatitis B are attributable to HDV infection, indicating that hepatitis D may be an important contributor to liver disease. It should be noted that there was a paucity of studies reporting anti-HDV prevalence among people with cirrhosis and HCC from some regions (including the South-East Asia region and the Western Pacific region), and therefore we estimated global PAFs and did not estimate regional PAFs. Our global PAF estimates should be considered preliminary until more data are made available to resolve sources of heterogeneity and obtain more globally representative estimates. Further, our estimates of attributable fraction are based on ascertainment of anti-HDV prevalence among individuals diagnosed with cirrhosis and HCC from cross-sectional studies. These may include individuals with early compensated cirrhosis. Since HDV accelerates disease progression, HDV may have an even greater attributable fraction of HBV-associated mortality from cirrhosis and HCC. As to the long-term impact of HDV RNA positivity on the risk of cirrhosis and HCC, we are limited by the available data, which very rarely include longitudinal follow-up to ascertain clinical outcomes. Ascertainment of the contribution of HDV to mortality is also not currently possible due to the lack of data.

In summary, the data indicate that HDV infection is common among HBsAg-positive people in the general population worldwide, albeit with significant geographic heterogeneity. The data also indicate that PWID, haemodialysis recipients, CSWs and MSM, as well as people with HIV and HCV, are at increased odds of HDV seropositivity, and that HDV is responsible for a substantial proportion of cirrhosis and HCC among HBsAg-positive people. HDV infection should be considered in all patients with chronic HBV infection. Routine HDV ascertainment in clinical settings is of particular importance in view of the suppressive effect of HDV co-infection on HBV DNA levels,^28^ which may lead to misclassification of HBV status. Furthermore, novel therapeutics that target HDV entry, prenylation and nucleic acid replication offer promise for the treatment of HDV infection.[29], [30], [31], [32] If all people newly diagnosed with chronic HBV infection had a routine reflex test for anti-HDV, surveillance and ascertainment of HDV-related disease would be improved. Public health agencies may want to report on HDV infection and implement activities to mitigate the risk of HDV acquisition according to their local epidemic. In addition to the adoption of routine testing for all HBsAg-positive people, the addition of anti-HDV/HDV RNA testing (including the use of dry blood spots) as part of representative population surveys such as demographic health surveys, would be useful in areas with sparse data.

Hepatitis D is widely distributed but neglected disease. A better description of the role that HDV plays in causing liver disease, better efforts to improve epidemiological data collection, ascertainment of temporal trends and identification of locally important risk factors would inform development of new medicines and help in mounting an effective public health response.

### Abbreviations

AFR, African Region; AMR, Region of the Americas; CSW, commercial sex workers; EMR, Eastern Mediterranean Region; EUR, European Region; HCC, hepatocellular carcinoma; MSM, men who have sex with men; OR, odds ratios; PAF, population attributable fraction; PWID, people who inject drugs; SEAR, South-East Asian Region; WHO, World Health Organisation; WPR, Western Pacific Region.

## Financial support

10.13039/100010269Wellcome Trust (United Kingdom) Clinical PhD Fellowship grant 109130/Z/15/Z (for AS), 10.13039/100004423World Health Organization (Geneva, Switzerland) (for AMG).

## Authors' contributions

AJS designed the study, performed searches, extracted data, assessed data quality, performed statistical analyses and wrote the manuscript; BK performed searches, extracted data, assessed data quality and reviewed the manuscript; MYRH assisted with statistical analyses, performed the PAF analysis and reviewed the manuscript; EG and IK developed the models, performed statistical analyses and reviewed the manuscript; CdM provided input into the analysis and reviewed the manuscript; YH provided input into study design and analysis and reviewed the manuscript; AMG supervised the study design, analysis and interpretation, and reviewed and revised the manuscript.

**PROSPERO Registration:** CRD42018113039.

## Disclaimer

The authors alone are responsible for the views expressed in this article and they do not necessarily represent the views, decisions or policies of the institutions with which they are affiliated.

## Conflict of interest

AMG reports personal payments and consulting honoraria from Roche Pharma Research & Early Development, Gilead, Janssen, and ViiV, and research funding from Roche Pharma Research & Early Development, Gilead, Janssen and ViiV, outside of the submitted work. Other authors do not declare any conflicts of interest.

Please refer to the accompanying ICMJE disclosure forms for further details.

## Supplementary data

Supplementary information.pdf

CTAT table.pdf
