# Supplementary material for: The global prevalence of hepatitis D virus infection: Systematic review and meta-analysis
Source: J Hepatol. 2020 Sep;73(3):523–32. doi: 10.1016/j.jhep.2020.04.008 (PMC7438974; doi:10.1016/j.jhep.2020.04.008)
Supplement: Supplementary information.pdf [file mmc1.pdf]

The global prevalence of hepatitis D virus infection: systematic review and meta-analysis

Alexander J. Stockdale, Benno Kreuels, Marc Y. R. Henrion, Emanuele Giorgi, Irene Kyomuhangi, Catherine de Martel, Yvan Hutin, Anna Maria Geretti

Table of contents

Supplementary appendix 1 3
Supplementary appendix 2 4
Supplementary appendix 3 5
Supplementary appendix 4 10
Supplementary appendix 5 11
Supplementary appendix 6 18
Supplementary appendix 7 22
Supplementary appendix 8 24
Supplementary appendix 9 26
Supplementary appendix 10 28
Supplementary appendix 11 29
Supplementary appendix 12 30
Supplementary appendix 13 31
Supplementary appendix 14 33
Supplementary appendix 15 34
Supplementary appendix 16 35
Supplementary appendix 17 36
Supplementary appendix 18 37
Supplementary appendix 19 38
Supplementary appendix 20 39
Supplementary appendix 21 40
Supplementary appendix 22 41

Supplementary appendix 23 46
Supplementary appendix 24 49
Supplementary appendix 25 50
Supplementary appendix 26 51
Supplementary appendix 27 52

Supplementary appendix 1. Search Terms for PubMed, EMBASE, and Scopus

PubMed (https://www.ncbi.nlm.nih.gov/pubmed/)

#1	Hepatitis D [MeSH] OR Hepatitis Delta Virus [MeSH] or hepatitis D virus [Title/Abstract] OR hepatitis d [Title/Abstract] OR hepatitis delta [Title/Abstract] OR hepatitis delta virus [Title/Abstract] OR HDV [Title/Abstract] OR delta virus [Title/Abstract]
#2	Epidemiology [MeSH] OR Prevalence [MeSH] OR prevalence [Title/Abstract] OR Incidence [MeSH] OR incidence [Title/Abstract] OR epidemiology [Title/Abstract] OR serosurvey [Title/Abstract] OR seroprevalence [Title/Abstract] OR anti-HDV [Title/Abstract] OR HDV RNA [Title/Abstract] OR anti-hepatitis delta virus antibody [Title/Abstract] OR ELISA [Title/Abstract] OR Enzyme-Linked Immunosorbent Assay[MeSH] OR enzyme linked immunosorbent assay [Title/Abstract] OR viraemia [Title/Abstract] OR viremia [Title/Abstract] OR Viremia [MeSH] OR Real-Time Polymerase Chain Reaction [MeSH] OR anti-delta antibody [Title/Abstract] OR "HDV IgG" [Title/Abstract] OR HDV-IgG [Title/Abstract] OR "HDV Ab" [Title/Abstract] OR HDVAb [Title/Abstract] OR HDV-Ab [Title/Abstract] OR "antibodies to HDV" [Title/Abstract] OR anti-HD [Title/Abstract]
#3	#1 AND #2

EMBASE (https://www.embase.com/login)

#1	exp DELTAVIRUS/ OR exp "HEPATITIS DELTA VIRUS"/
#2	(hepatitis delta virus OR hdv OR hepatitis d OR delta virus).ti,ab
#3	#1 OR #2
#4	PREVALENCE/ OR EPIDEMIOLOGY/ OR "PREVALENCE STUDY"/ OR "PREVALENCE, SERO"/
#5	(prevalence OR epidemiology OR incidence OR serosurvey OR seroprevalence OR "HDV RNA" OR "hepatitis delta virus ribonucleic acid" OR "anti-HDV" OR "HDV IgG" OR "HDV-IgG" OR "HDV Ab" OR "HDVAb" OR "HDV-Ab" OR "antibodies to HDV" OR "anti-HD" OR "anti-delta antibody" OR "anti-hepatitis delta virus antibody" OR ELISA OR "enzyme linked immunosorbent assay" OR viraemia OR viremia).ti,ab
#6	#4 OR #5
#7	#3 AND #6

SCOPUS (https://www.scopus.com/home.uri)

#1	(TITLE-ABS-KEY ("hepatitis d") OR TITLE-ABS-KEY ("hepatitis delta virus") OR TITLE-ABS-KEY ("delta virus") OR TITLE-ABS-KEY ("hepatitis delta") OR TITLE-ABS-KEY (HDV) OR TITLE-ABS-KEY("hepatitis d virus")) AND (TITLE-ABS-KEY (incidence) OR TITLE-ABS-KEY (prevalence) OR TITLE-ABS-KEY (epidemiology) OR TITLE-ABS-KEY (seroprevalence) OR TITLE-ABS-KEY (serosurvey) OR TITLE-ABS-KEY (ELISA) OR TITLE-ABS-KEY ("enzyme linked immunosorbent assay") OR TITLE-ABS-KEY (viraemia) OR TITLE-ABS-KEY (viremia) OR TITLE-ABS-KEY (anti-HDV) OR TITLE-ABS-KEY ("anti HDV") OR TITLE-ABS-KEY ("HDV IgG") OR TITLE-ABS-KEY (HDV-IgG) OR TITLE-ABS-KEY ("HDV Ab") OR TITLE-ABS-KEY ("HDV-Ab") OR TITLE-ABS-KEY ("antibodies to HDV") OR TITLE-ABS-KEY ("anti-HD") OR TITLE-ABS-KEY ("anti-hepatitis delta") OR TITLE-ABS-KEY ("anti-delta antibody") OR TITLE-ABS-KEY ("HDV RNA") OR TITLE-ABS-KEY ("hepatitis delta RNA") OR TITLE-ABS-KEY ("polymerase chain reaction") OR TITLE-ABS-KEY (PCR)) AND PUBYEAR > 1989

Supplementary appendix 2. Sources of Grey Literature Search

We performed searches of surveys, censuses, vital statistics, and reports using the search terms "hepatitis D" OR "HDV" OR "anti-HDV" OR "hepatitis delta" OR "delta virus", and screened all articles with the keyword "hepatitis" within the following sources:

1	Global Health Data Exchange http://ghdx.healthdata.org/
2	The Demographic and Health Survey Programmes (https://www.dhsprogram.com/)
3	UNICEF Multiple Indicator Cluster Surveys (http://mics.unicef.org/)
4	World Bank Living Standards Measurement Survey (http://iresearch.worldbank.org/lsms/lsmssurveyFinder.htm)
5	Victoria State (Australia) Health Infectious Diseases Surveillance (https://www2.health.vic.gov.au/public-health/infectious-diseases/infectious-diseases-surveillance)
6	Brazil Health Information (TABNET) (http://tabnet.datasus.gov.br/cgi/deftohtm.exe?sinannet/cnv/hepabr.def)
7	Infectious Disease Epidemiology Annual Report – 2016 Robert Koch Institute (Germany) (https://www.rki.de/DE/Content/Infekt/Jahrbuch/Jahrbuecher/2017.html;jsessionid=055F194F3ED98490F0E98039C03B28A9.1_cid290?nn=2374622
8	Public Health England (United Kingdom); Sentinel surveillance of blood borne virus testing in England: 2013-2017 (https://www.gov.uk/government/publications/sentinel-surveillance-of-blood-borne-virus-testing-in-england-2017)

Supplementary appendix 3. Included Studies by WHO region: General Populations

African Region (AFR)

AF1. Ampah KA, Pinho-Nascimento CA, Kerber S, et al. Limited Genetic Diversity of Hepatitis B Virus in the General Population of the Offin River Valley in Ghana. *PloS One* 2016; 11: e0156864.

AF2. Andernach IE, Leiss LV, Tarnagda ZS, et al. Characterization of hepatitis delta virus in sub-Saharan Africa. *J Clin Microbiol* 2014; 52: 1629-36.

AF3. Andersson MI, Maponga TG, Ijaz S, et al. The epidemiology of hepatitis B virus infection in HIV-infected and HIV-uninfected pregnant women in the Western Cape, South Africa. *Vaccine* 2013; 31: 5579-84.

AF4. Belyhun Y, Liebert UG, Maier M. Clade homogeneity and low rate of delta virus despite hyperendemicity of hepatitis B virus in Ethiopia. *Virol J* 2017; 14: 176.

AF5. Butler EK, Rodgers MA, Coller KE, et al. High prevalence of hepatitis delta virus in Cameroon. *Sci Rep* 2018; 8: 11617.

AF6. Coffie PA, Tchounga BK, Bado G, et al. Prevalence of hepatitis B and delta according to HIV-type: a multi-country cross-sectional survey in West Africa. *BMC Infect Dis* 2017; 17: 466.

AF7. Cunha L, Plouzeau C, Ingrand P, et al. Use of replacement blood donors to study the epidemiology of major blood-borne viruses in the general population of Maputo, Mozambique. *J Med Virol* 2007; 79: 1832-40.

AF8. De Paschale M, Ceriani C, Cerulli T, et al. Prevalence of HBV, HDV, HCV, and HIV infection during pregnancy in northern Benin. *J Med Virol* 2014; 86: 1281-7.

AF9. Ducancelle A, Abgueguen P, Birguel J, et al. High endemicity and low molecular diversity of hepatitis B virus infections in pregnant women in a rural district of North Cameroon. *PloS One* 2013; 8: e80346.

AF10. Francois-Souquiere S, Makuwa M, Bisvigou U, Kazanji M. Epidemiological and molecular features of hepatitis B and hepatitis delta virus transmission in a remote rural community in central Africa. *Infect Genet Evol* 2016; 39: 12-21.

AF11. Groc S, Abbate JL, Le Gal F, et al. High prevalence and diversity of hepatitis B and hepatitis delta virus in Gabon. *J Viral Hepat* 2019; 26: 170-82.

AF12. Honge BL, Jespersen S, Medina C, et al. Hepatitis B and delta virus are prevalent but often subclinical co-infections among HIV infected patients in Guinea-Bissau, West Africa: a cross-sectional study. *PloS One* 2014; 9: e99971.

AF13. Ifeorah IM, Bakarey AS, Adeniji JA, Onyemelukwe FN. Seroprevalence of hepatitis B and delta viruses among HIV-infected population attending anti-retroviral clinic in selected health facilities in Abuja, Nigeria. *J Immunoassay Immunochem* 2017; 38: 608-19.

AF14. Jaquet A, Wandeler G, Nouaman M, et al. Alcohol use, viral hepatitis and liver fibrosis among HIV-positive persons in West Africa: a cross-sectional study. *J Int AIDS Soc* 2017; 19: 21424.

AF15. Katwesigye E, Seremba E, Semitala F, Ocama P. Low sero-prevalence of hepatitis delta antibodies in HIV/ hepatitis B co-infected patients attending an urban HIV clinic in Uganda. *African Health Sciences* 2016; 16: 1089-93.

AF16. Komas NP, Ghosh S, Abdou-Chekaraou M, et al. Hepatitis B and hepatitis D virus infections in the Central African Republic, twenty-five years after a fulminant hepatitis outbreak, indicate continuing spread in asymptomatic young adults. *PLoS Negl Trop Dis* 2018; 12: e0006377.

AF17. Lemoine M, Shimakawa Y, Njie R, et al. Acceptability and feasibility of a screen-and-treat programme for hepatitis B virus infection in The Gambia. *Lancet Glob Health* 2016; 4: e559-67.

AF18. Makuwa M, Caron M, Souquiere S, Malonga-Mouelet G, Mahe A, Kazanji M. Prevalence and genetic diversity of hepatitis B and delta viruses in pregnant women in Gabon: Molecular evidence that hepatitis delta virus clade 8 originates from and is endemic in central Africa. *J Clin Microbiol* 2008; 46: 754-6.

AF19. Makuwa M, Mintsa-Ndong A, Souquiere S, Nkoghe D, Leroy EM, Kazanji M. Prevalence and molecular diversity of hepatitis B virus and hepatitis delta virus in urban and rural populations in northern Gabon in central Africa. *J Clin Microbiol* 2009; 47: 2265-8.

AF20. Mansour W, Bollahi MA, Hamed CT, et al. Virological and epidemiological features of hepatitis delta infection among blood donors in Nouakchott, Mauritania. *J Clin Virol* 2012; 55: 12-6.
AF21. Mansour W, Malick FZ, Sidiya A, et al. Prevalence, risk factors, and molecular epidemiology of hepatitis B and hepatitis delta virus in pregnant women and in patients in Mauritania. *J Med Virol* 2012; 84: 1186-98.
AF22. Matthews PC, Beloukas A, Malik A, et al. Prevalence and characteristics of hepatitis B virus (HBV) coinfection among HIV-positive women in South Africa and Botswana. *PloS One* 2015; 10: e0134037.
AF23. Njouom R, Tejiokem MC, Texier G, Fontanet A. Prevalence of hepatitis B, hepatitis C and hepatitis D virus infections in Cameroon: Results from a national population based survey. *J Viral Hepat* 2015; 22: 1-2.
AF24. Pinho-Nascimento CA, Bratschi MW, Hofer R, et al. Transmission of hepatitis B and D viruses in an African rural community. *mSystems* 2018; 3.
AF25. Salpini R, Fokam J, Ceccarelli L, et al. High burden of HBV-infection and atypical HBV strains among HIV-infected cameroonians. *Curr HIV Res* 2016; 14: 165-71.
AF26. Sanou AM, Benkirane K, Tinto B, et al. Prevalence of hepatitis B virus and hepatitis D virus coinfection in western Burkina Faso and molecular characterization of the detected virus strains. *Int J Infect Dis* 2018; 70: 15-9.
AF27. Stockdale AJ, Chaponda M, Beloukas A, et al. Prevalence of hepatitis D virus infection in sub-Saharan Africa: a systematic review and meta-analysis. *Lancet Glob Health* 2017; 5: e992-e1003.
AF28. Torimiro JN, Nanfack A, Takang W, et al. Rates of HBV, HCV, HDV and HIV type 1 among pregnant women and HIV type 1 drug resistance-associated mutations in breastfeeding women on antiretroviral therapy. *BMC Pregnancy Childbirth* 2018; 18: 504.
AF29. Tuaillon E, Kania D, Gordien E, Van de Perre P, Dujols P. Epidemiological data for hepatitis D in Africa. *Lancet Glob Health* 2018; 6: e33.
AF30. Vray M, Debonne JM, Sire JM, et al. Molecular epidemiology of hepatitis B virus in Dakar, Senegal. *J Med Virol* 2006; 78: 329-34.
AF31. Wilson P, Jhaveri R, Parr JB, et al. Efficient dried blood spot based determination of hepatitis B seroprevalence from a national survey in the Democratic Republic of the Congo. *J Viral Hepat* 2018; 25: 92-3.
AF32. Winter A, Letang E, Vedastus Kalinjuma A, et al. Absence of hepatitis delta infection in a large rural HIV cohort in Tanzania. *Int J Infect Dis* 2016; 46: 8-10.

Eastern Mediterranean Region (EMR)

EM1. Alavian SM, Tabatabaei SV, Ghadimi T, et al. Seroprevalence of HBV infection and its risk factors in the west of Iran: A population-based study. *Int J Prev Med* 2012; 3: 770-5.
EM2. Alavian SM, Tabatabaei SV, Nourizad S, et al. Seroepidemiology of HBV infection in Kermanshah, west of Iran; a population based study. *Jundishapur J Microbiol* 2012; 5: 564-9.
EM3. Ali HYM, Yassen SA. Prevalence of hepatitis B & D viral infections among hospital personnel in Mosul Iraq. *Qatar Med J* 2001; 10: 51-3.
EM4. Al-Nabehi BAH, Saeed WSE, Musa AM, El Hassan AM, Khalil EAG, Al-Shamahy H. Sero-molecular epidemiology and risk factors of viral hepatitis in urban Yemen. *Int J Virol* 2015; 11: 133-8.
EM5. Al-Traif I, Ali A, Dafalla M, Al-Tamimi W, Qassem L. Prevalence of hepatitis delta antibody among HBsAg carriers in Saudi Arabia. *Ann Saudi Med* 2004; 24: 343-4.
EM6. Attaran MS, Sharifi Z, Hosseini SM, Samei S, Ataee Z. Prevalence of hepatitis B and hepatitis D coinfection in asymptomatic blood donors in Iran. *Apmis* 2014; 122: 243-7.
EM7. Djebbi A, Rebai WK, Bahri O, Hogga N, Sadraoui A, Triki H. [serological markers, viral RNA and genotype of hepatitis delta virus in HBs antigen positive Tunisian patients]. *Pathol Biol* 2009; 57: 518-23.
EM8. Doosti A, Amini-Bavil-Olyaee S, Tajbakhsh E, Adeli A, Mahboudi F. Prevalence of viral hepatitis and molecular analysis of HBV among voluntary blood donors in west Iran. *New Microbiol* 2009; 32: 193-8.

EM9. Elashry A, Gomaa A, Elzohry HA, Badra G, Waked I, Raouf AA. Prevalence and risk-factors of hepatitis virus markers in Egyptian young men: Evidence for decreasing prevalence of hepatitis C. *Hepatology* 2015; 62.
EM10. Ghadir MR, Belbasi M, Heidari A, et al. Prevalence of hepatitis D virus infection among hepatitis B virus infected patients in Qom province, center of Iran. *Hepat Mon* 2012; 12: 205-8.
EM11. Gomaa NI, Metwally LA, Nemr N, Younis S. Seroprevalence of HDV infection in HBsAg positive population in Ismailia, Egypt. *Egypt J Immunol* 2013; 20: 23-8.
EM12. Javanmard D, Abedi F, Namaei MH, Asghari A, Ziaee M, Alavian SM. High prevalence of hepatitis B virus infection in the village of Esfandiar in south Khorasan province, Iran. *Hepat Mon* 2018; 18.
EM13. Ramia S, El-Zaatari M, Sharara AI, Ramlawi F, Farhat B. Current prevalence of hepatitis delta virus (HDV) infection and the range of HDV genotypes in Lebanon. *Epidemiol Infect* 2007; 135: 959-62.
EM14. Roshandel G, Semnani S, Abdolahi N, et al. Prevalence of hepatitis D virus infection in hepatitis B surface antigen-positive subjects in Golestan province, northeast Iran. *J Microbiol Immunol Infect* 2008; 41: 227-30.
EM15. Salehi M, Alavian SM, Tabatabaei SV, et al. Seroepidemiology of HBV infection in south-east of Iran; a population based study. *Iran Red Crescent Med J* 2012; 14: 283-8.
EM16. Saudy N, Sugauchi F, Tanaka Y, et al. Genotypes and phylogenetic characterization of hepatitis B and delta viruses in Egypt. *J Med Virol* 2003; 70: 529-36.
EM17. Sharifzadeh G, Namaei MH, Ebrahimzadeh A, et al. Prevalence of hepatitis D virus infection and associated factors among HBsAg-positive patients in Birjand, Iran, 2012-2014. *Hepat Mon* 2017; 17.
EM18. Tajbakhsh E, Abbasian B, Esmaeili A, Behjati M. Seroprevalence of anti-HDV Ab and socioepidemiological characteristics among HBsAg-positive blood donors in Charmahal-O-Bakhtiyari province, Iran. *Hepat Mon* 2011; 11: 130-1.
EM19. Ur Rahman S, Hafiz A. Study of hepatitis D in high risk population of hepatitis B. *J Coll Physicians Surg Pak*; 10: 29-32.
EM20. Zaidi G, Idrees M, Malik FA, et al. Prevalence of hepatitis delta virus infection among hepatitis B virus surface antigen positive patients circulating in the largest province of Pakistan. *Virol J* 2010; 7: 283.

European Region (EUR)

EU1. Bissinger AL, Berg CP. Is the HDV seroprevalence in patients admitted to a university hospital representative for all HBV carriers in Germany? *Infection* 2013; 41: 1031-2.
EU2. Brehar-Cioflec D, Claici C, Rosiu N, Negrea DA, Moldovan R, Cosnita M. Hepatitis B virus (HBV) and dual HBV-hepatitis delta virus (HDV) infection in apparently healthy persons. *Rom J Virol* 1998; 49: 3-10.
EU3. Cozzolongo R, Elba S, Petruzzi J, et al. Epidemiology of HCV and HBV in the general population: A survey in a southern Italian town. *J Hepatology* 2009; 50.
EU4. Drobeniuc J, Hutin YJ, Harpaz R, et al. Prevalence of hepatitis B, D and C virus infections among children and pregnant women in Moldova: additional evidence supporting the need for routine hepatitis B vaccination of infants. *Epidemiol Infect* 1999; 123: 463-7.
EU5. Iliescu EL. Epidemiological study of HBV, HCV, HDV and HEV prevalence in Romania. *J Gastroenterol Hepatology* 2013; 28: 423-4.
EU6. Kalinin AL, Zhavoronok SV, Mikhailov MI, et al. [Viral hepatitis delta in the republic of Belarus]. *Zh Mikrobiol Epidemiol Immunobiol* 1998; 6: 74-7.
EU7. Mese S, Nergiz S, Tekes S, Gul K. Seroprevalence of serum HBsAg positivity and hepatitis delta virus infection among blood donors in south-eastern Turkey. *Clin Ter* 2014; 165: 95-8.
EU8. Quaglio G, Ramadani N, Pattaro C, et al. Prevalence and risk factors for viral hepatitis in the Kosovarian population: implications for health policy. *J Med Virol* 2008; 80: 833-40.

EU9. Rosenberg GK, Lattimore S, Brailsford SR, et al. The diversity of chronic hepatitis B virus infections within blood donors in England and North Wales 2005 through 2010. *Transfusion* 2013; 53: 2467-76.
EU10. Sellier PO, Maylin S, Brichler S, et al. Hepatitis B virus-hepatitis D virus mother-to-child co-transmission: A retrospective study in a developed country. *Liver Int* 2018; 38: 611-8.
EU11. Servant-Delmas A, Le Gal F, Gallian P, Gordien E, Laperche S. Increasing prevalence of HDV/HBV infection over 15 years in France. *J Clin Virol* 2014; 59: 126-8.
EU12. Tozun N, Ozdogan O, Cakaloglu Y, et al. Seroprevalence of hepatitis B and C virus infections and risk factors in Turkey: A fieldwork TURHEP study. *Clin Microbiol Infect* 2015; 21: 1020-6.
EU13. Uzun B, Sener AG, Gungor S, Afsar I, Demirci M. Evaluation of hepatitis delta virus (HDV) infection in blood donors in western Turkey. *Transfus Apher Sci* 2014; 50: 388-91.
EU14. Voiculescu M, Iliescu L, Ionescu C, et al. A cross-sectional epidemiological study of HBV, HCV, HDV and HEV prevalence in the SubCarpathian and South-Eastern regions of Romania. *J Gastrointestin Liver Dis* 2010; 19: 43-8.

Region of the Americas (AMR)
AM1. Cabezas J, Trujillo O, Balbuena J, Borda A, Fiestas V, Ramos F. Impact of 20 years of implementation of an immunization program for HBV in Huanta, Peru: First clues of elimination of hepatitis delta virus. *Hepatology* 2018; 68.
AM2. Caetano KAA, Pinheiro RS, Matos MA, et al. Hepatitis B in rural settlement projects in central Brazil: Epidemiology and vaccination. *J Viral Hepat* 2015; 22: 23-4.
AM3. Crispim MA, Fraiji NA, Campello SC, Schriefer NA, Stefani MM, Kiesslich D. Molecular epidemiology of hepatitis B and hepatitis delta viruses circulating in the western Amazon region, north Brazil. *BMC Infect Dis* 2014; 14: 94.
AM4. Delfino CM, Gentile EA, Castillo AI, et al. Hepatitis B virus and hepatitis D virus in blood donors from Argentina: Circulation of HBsAg and reverse transcriptase mutants. *Arch Virol* 2014; 159: 1109-17.
AM5. Njei B, Do A, Lim JK. Prevalence of hepatitis delta infection in the United States: National health and nutrition examination survey, 1999-2012. *Hepatology* 2016; 64: 681-2.
AM6. Villar LM, Milagres FAP, Lampe E, et al. Determination of hepatitis B, C and D prevalence among urban and Amerindian populations from the eastern Brazilian Amazon: A cross sectional study. *BMC Infect Dis* 2018; 18: 411.

South-East Asia Region (SEAR)
SE1. Louisirirotchanakul S, Myint KS, Srimee B, et al. The prevalence of viral hepatitis among the Hmong people of northern Thailand. *Southeast Asian J Trop Med Public Health* 2002; 33: 837-44.
SE2. Lusida MI, Surayah, Sakugawa H, et al. Genotype and subtype analyses of hepatitis B virus (HBV) and possible co-infection of HBV and hepatitis C virus (HCV) or hepatitis D virus (HDV) in blood donors, patients with chronic liver disease and patients on hemodialysis in Surabaya, Indonesia. *Microbiol Immunol* 2003; 47: 969-75.
SE3. Mollah AH, Nahar N, Siddique MA, Anwar KS, Hassan T, Azam MG. Common transfusion-transmitted infectious agents among thalassaemic children in Bangladesh. *J Health Popul Nutr* 2003; 21: 67-71.
SE4. Theamboonlers A, Hansurabhanon T, Verachai V, Chongsrisawat V, Poovorawan Y. Hepatitis D virus infection in Thailand: HDV genotyping by RT-PCR, RFLP and direct sequencing. *Infection* 2002; 30: 140-4.

Western Pacific Region (WPR)
WP1. Arakawa Y, Moriyama M, Taira M, et al. Molecular analysis of hepatitis D virus infection in Miyako Island, a small Japanese island. *J Viral Hepat* 2000; 7: 375-81.
WP2. Chang CJ, Chiang JC, Lu SN, Wang JH. Hepatitis delta virus and GBV-C infection in two neighboring hepatitis B virus and hepatitis C virus-endemic villages in Taiwan. *Chang Gung Med J* 2010; 33: 137-44.

WP3. Chang SY, Yang CL, Ko WS, et al. Molecular epidemiology of hepatitis D virus infection among injecting drug users with and without human immunodeficiency virus infection in Taiwan. *J Clin Microbiol* 2011; 49: 1083-9.
WP4. Chen F, Zhang J, Guo F, et al. Hepatitis B, C, and D virus infection showing distinct patterns between injection drug users and the general population. *J Gastroenterol Hepatol* 2017; 32: 515-20.
WP5. Chen X, Oidovsambuu O, Liu P, et al. A novel quantitative microarray antibody capture assay identifies an extremely high hepatitis delta virus prevalence among hepatitis B virus-infected Mongolians. *Hepatology* 2017; 66: 1739-49.
WP6. Davaalkham D, Ojima T, Uehara R, et al. Hepatitis delta virus infection in mongolia: analyses of geographic distribution, risk factors, and disease severity. *Am J Trop Med Hyg* 2006; 75: 365-9.
WP7. Davies J, Tong SY, Davis JS. Hepatitis D is rare or non-existent in hepatitis B virus-infected Indigenous Australians in the Northern Territory. *Aust N Z J Public Health* 2013; 37: 188-9.
WP8. Dunford L, Carr MJ, Dean J, et al. A multicentre molecular analysis of hepatitis B and blood-borne virus coinfections in Viet Nam. *PLoS One* 2012; 7: e39027.
WP9. Inoue J, Takahashi M, Nishizawa T, et al. High prevalence of hepatitis delta virus infection detectable by enzyme immunoassay among apparently healthy individuals in Mongolia. *J Med Virol* 2005; 76: 333-40.
WP10. Lee MH, You SL, Yang HI, et al. Prevalence and risk factors of hepatitis D virus infection: the REVEAL-HBV/HCV/HDV study. *Hepatology Intern* 2014; 8.
WP11. Li L, He J, Zhao L. [Epidemiologic features of viral hepatitis in Fujian]. *Zhonghua Liu Xing Bing Xue Za Zhi* 1998; 19: 89-92.
WP12. Liu KSH, Seto WK, Lau EHY, et al. A territory-wide prevalence study on blood-borne and enteric viral hepatitis in Hong Kong. *J Infect Dis* 2019.
WP13. Lu SN, Chen TM, Lee CM, Wang JH, Tung HD, Wu JC. Molecular epidemiological and clinical aspects of hepatitis D virus in a unique triple hepatitis viruses (B, C, D) endemic community in Taiwan. *J Med Virol* 2003; 70: 74-80.
WP14. Nguyen VT, McLaws ML, Dore GJ. Highly endemic hepatitis B infection in rural Vietnam. *J Gastroenterol Hepatol* 2007; 22: 2093-100.
WP15. Tsatsralt-Od B, Takahashi M, Nishizawa T, Inoue J, Ulaankhuu D, Okamoto H. High prevalence of hepatitis B, C and delta virus infections among blood donors in Mongolia. *Arch Virol* 2005; 150: 2513-28.

Supplementary appendix 4. Included Studies by WHO Region: Isolated Populations

European Region (EUR)

EU15. Borresen ML, Olsen OR, Ladefoged K, et al. Hepatitis D outbreak among children in a hepatitis B hyper-endemic settlement in Greenland. *J Viral Hep* 2010; 17: 162-70.

EU16. Karatapanis S, Papastergiou V, Papakonstantinou F, et al. Sixteen years after the implementation of compulsory hepatitis B vaccination in a hepatitis delta endemic area in Greece: Comparison between two population based serosurveys. *J Viral Hep* 2018; 25: 188-9.

EU17. Rex KF, Krarup HB, Laurberg P, Andersen S. Population-based comparative epidemiological survey of hepatitis B, D, and C among Inuit migrated to Denmark and in high endemic Greenland. *Scand J Gastroenterol* 2012; 47: 692-701.

Region of the Americas (AMR)

AM7. Braga WS, Brasil LM, de Souza RA, Castilho Mda C, da Fonseca JC. [The occurrence of hepatitis B and delta virus infection within seven Amerindian ethnic groups in the Brazilian western Amazon]. *Rev Soc Bras Med Trop* 2001; 34: 349-55.

AM8. Braga WS, Castilho Mda C, Borges FG, et al. Hepatitis D virus infection in the Western Brazilian Amazon - far from a vanishing disease. *Rev Soc Bras Med Trop* 2012; 45: 691-5.

AM9. de Paula VS, Arruda ME, Vitral CL, Gaspar AM. Seroprevalence of viral hepatitis in riverine communities from the Western Region of the Brazilian Amazon Basin. *Mem Inst Oswaldo Cruz* 2001; 96: 1123-8.

AM10. di Filippo Villa D, Cortes-Mancera F, Payares E, et al. Hepatitis D virus and hepatitis B virus infection in Amerindian communities of the Amazonas state, Colombia. *Virol J* 2015; 12: 172.

AM11. Duarte MC, Cardona N, Poblete F, et al. A comparative epidemiological study of hepatitis B and hepatitis D virus infections in Yanomami and Piaroa Amerindians of Amazonas State, Venezuela. *Trop Med Int Health* 2010; 15: 924-33.

AM12. Harpaz R, McMahon BJ, Margolis HS, et al. Elimination of new chronic hepatitis B virus infections: results of the Alaska immunization program. *J Infect Dis* 2000; 181: 413-8.

AM13. Leon P, Venegas E, Bengoechea L, et al. [Prevalence of infections by hepatitis B, C, D and E viruses in Bolivia]. *Rev Panam Salud Publica* 1999; 5: 144-51.

AM14. Nunes HM, Monteiro MR, Soares Mdo C. [Prevalence of hepatitis B and D serological markers in the Parakana, Apyterewa Indian Reservation, Para State, Brazil]. *Cad Saude Publica* 2007; 23: 2756-66.

AM15. Viana S, Parana R, Moreira RC, Compri AP, Macedo V. High prevalence of hepatitis B virus and hepatitis D virus in the western Brazilian Amazon. *Am J Trop Med Hyg* 2005; 73: 808-14.

AM6. Villar LM, Milagres FAP, Lampe E, et al. Determination of hepatitis B, C and D prevalence among urban and Amerindian populations from the Eastern Brazilian Amazon: a cross sectional study. *BMC Infect Dis* 2018; 18: 411.

South-East Asia Region (SEAR)

SE5. Borkakoty BJ, Biswas D, Mahanta J. Delta hepatitis infection in northeast India [3]. *Indian J Gastroenterol* 2007; 26: 251-2.

SE6. Murhekar MV, Murhekar KM, Arankalle VA, Sehgal SC. Hepatitis delta virus infection among the tribes of the Andaman and Nicobar Islands, India. *Trans R Soc Trop Med Hyg* 2005; 99: 483-4.

Supplementary appendix 5. Included Studies by WHO Region: Hepatology Clinic Populations

African Region (AFR)

AF33. Abdulkareem LO, Uhunmwangho AO, Ndububa DA, Yunusa T. Seroprevalence of delta hepatitis antibodies, liver function test profile and severity of liver disease among individuals with chronic hepatitis B infection seen in a tertiary hospital in Abuja, Nigeria. *United Eur Gastroenterol J* 2018; 6.

AF34. Aberra H, Gordien E, Desalegn H, et al. Hepatitis delta virus infection in a large cohort of chronic hepatitis B patients in Ethiopia. *Liver Int* 2017.

AF35. Amougou MA, Noah DN, Moundipa PF, Pineau P, Njouom R. A prominent role of Hepatitis D Virus in liver cancers documented in Central Africa. *BMC Infect Dis* 2016; 16: 647.

AF2. Andernach IE, Leiss LV, Tarnagda ZS, et al. Characterization of hepatitis delta virus in sub-Saharan Africa. *J Clin Microbiol* 2014; 52: 1629-36.

AF36. Anyovi F, Lahaye FM, Rapp C, et al. Molecular epidemiology of hepatitis D virus in Togo. *Trop Med Int Health* 2017; 22: 133-4.

AF37. Asmah RH, Boamah I, Afodzinu M, et al. Prevalence of hepatitis d infection in patients with hepatitis B virus-related liver diseases in Accra, Ghana. *West Afr J Med* 2014; 33: 32-6.

AF4. Belyhun Y, Liebert UG, Maier M. Clade homogeneity and low rate of delta virus despite hyperendemicity of hepatitis B virus in Ethiopia. *Virol J* 2017; 14: 176.

AF38. Foupouapouognigni Y, Noah DN, Sartre MT, Njouom R. High prevalence and predominance of hepatitis delta virus genotype 1 infection in Cameroon. *J Clin Microbiol* 2011; 49: 1162-4.

AF39. Gourari S, Brichler S, Le Gal F, et al. Hepatitis B virus and hepatitis delta virus subtypes circulating in Algeria and seroprevalence of HDV infection. *J Med Virol* 2019; 91: 72-80.

AF40. Lunel-Fabiani F, Mansour W, Amar AO, et al. Impact of hepatitis B and delta virus co-infection on liver disease in Mauritania: a cross sectional study. *J Infect* 2013; 67: 448-57.

AF41. Mahale P, Aka P, Chen X, et al. Hepatitis D virus infection, cirrhosis, and hepatocellular carcinoma in The Gambia. *J Viral Hepat* 2019.

AF21. Mansour W, Malick FZ, Sidiya A, et al. Prevalence, risk factors, and molecular epidemiology of hepatitis B and hepatitis delta virus in pregnant women and in patients in Mauritania. *J Med Virol* 2012; 84: 1186-98.

AF42. Mbaye PS, Renaudineau Y, Diallo A, et al. [Hepatitis C virus and chronic hepatopathies in Dakar: case-control study]. *Med Trop* 2000; 60: 47-52.

AF43. Nwokediuko SC, Ijeoma U. Seroprevalence of antibody to HDV in Nigerians with hepatitis B virus-related liver diseases. *Niger J Clin Pract* 2009; 12: 439-42.

AF44. Olal SO, Akere A, Otegbayo JA, et al. Are patients with primary hepatocellular carcinoma infectious of hepatitis B, C and D viruses? *Afr J Med Med Sci* 2012; 41 Suppl: 187-91.

AF45. Onyekwere CA, Audu RA, Duro-Emmanuel F, Ige FA. Hepatitis D infection in Nigeria. *Indian J Gastroenterol* 2012; 31: 34-5.

AF46. Opaleye OO, Japhet OM, Adewumi OM, et al. Molecular epidemiology of hepatitis D virus circulating in Southwestern Nigeria. *Virol J* 2016; 13: 61.

AF26. Sanou AM, Benkirane K, Tinto B, et al. Prevalence of Hepatitis B virus and Hepatitis D virus Coinfection in Western Burkina Faso and molecular characterization of the detected virus strains. *Int J Infect Dis* 2018; 70: 15-9.

AF30. Vray M, Debonne JM, Sire JM, et al. Molecular epidemiology of hepatitis B virus in Dakar, Senegal. *J Med Virol* 2006; 78: 329-34.

Eastern Mediterranean Region (EMR)

EM21. Abbasi A, Bhutto AR, Butt N, Mahmood K. HDV seroprevalence in HBsAg positive patients. *J Coll Physicians Surg Pak* 2014; 24: 624-7.

EM22. Alavian SM, Imanieh MH, Imanieh MH. Predictive Factors in the Incidence of Cirrhosis in Chronic Hepatitis B Virus Infections. *Hepat Mon* 2016; 16: e34790.

EM5. Al-Traif I, Ali A, Dafalla M, Al-Tamimi W, Qassem L. Prevalence of hepatitis delta antibody among HBsAG carriers in Saudi Arabia. *Ann Saudi Med* 2004; 24: 343-4.

EM23. Ataei B, Yazdani MR, Kalantari H, et al. Hepatitis D virus infection in Isfahan, central Iran: Prevalence and risk factors among chronic HBV infection cases. *Hepat Mon* 2011; 11: 269-72.
EM24. Bakhshipour A, Mashhadi M, Mohammadi M, Nezam SK. Seroprevalence and risk factors of hepatitis delta virus in chronic hepatitis B virus infection in Zahedan. *Acta Med Iran* 2013; 51: 260-4.
EM25. Das K, Ali H, Mahmood T, Munir SM, Ahmed T, Farooq MU. Comparative analysis of disease activity in patients of chronic hepatitis B virus, with and without superinfection with hepatitis D virus; an experience at tertiary care centre. *J Ayub Med Coll Abbottabad* 2008; 20: 39-42.
EM26. Elzouki AN, Bashir SM, Elahmer O, Elzouki I, Alkhattali F. Prevalence and risk factors of hepatitis D virus infection in patients with chronic hepatitis B infection attending the three main tertiary hospitals in Libya. *Arab J Gastroenterol* 2017; 18: 216-9.
EM27. Faiz MS, Sheikh S, Mughal SA. Frequency of hepatitis-D in HBsAg positive cases at Peoples University of Medical & Health Sciences for Women Hospital Nawabshah. *Med Forum Mon* 2012; 23: 52-4.
EM28. Fouad R, Abdo M, Eldeen HG, et al. Influence of delta virus infection on the virologic status in Egyptian patients with chronic hepatitis B virus genotype D. *J Med Virol* 2016; 88: 837-42.
EM29. Hajiani E, Hashemi SJ, Masjedizadeh R, Ahmadzadeh S. Liver cirrhosis seen in GI clinics of Ahvaz, Iran. *Govaresh* 2012; 17: 178-82.
EM30. Hosseini S, Esmaeilzadeh A, Mokhtarifar A, Vosughi H, Saadatnia H, Khosravi A. Seroepidemiology And Co-Infections of Hepatitis D Virus Infection in the North-East of Iran. *Med J Mashhad Univ Med Sciences* 2010; 53: 69-72.
EM31. Jalil I, Arshad M, Rafaque Z, et al. Seroprevalence of HDV among non-hospitalized HBsAg positive patients from KPK-region of Pakistan. *Asian Pacific J Trop Biomed* 2016; 6: 609-13.
EM32. Jamjoom GA, El-Daly MM, Azhar EI, et al. Prevalence and molecular characterization of hepatitis D virus in Saudi Arabia: A single-center study. *Saudi J Gastroenterol* 2017; 23: 176-82.
EM33. Keshvari M, Alavian SM, Aghaee B, et al. Seroepidemiology and clinical features of hepatitis delta among HBsAg carriers: a study from hepatitis clinic of Iranian Blood Transfusion Organization. *Transfus Med* 2014; 24: 411-7.
EM34. Khan AA, Khalil-ur-Rehman A, Haider Z, Shafqat F. Seromarkers of hepatitis B and C in patients with cirrhosis. *J Coll Physicians Surgeons Pakistan* 2002; 12 105-7.
EM35. Khazaee T, Ebrahimzadeh A, Moghaddam E, Ghafori M. Assessment of prevalence and determine infections of hepatitis C and hepatitis D in patients with chronic hepatitis B. *Govaresh* 2016; 20: 230-6.
EM36. Lal N, Memon MS, Zaki M, et al. Clinical and virological characteristics of hepatitis B & D in chronically infected patients with respect to level of education in Pakistan. *Hepat Intern* 2011; 5: 205-6.
EM37. Mhalla S, Kadri Y, Alibi S, Letaief A, Boukadida J, Hannachi N. Hepatitis D virus infection among hepatitis B surface antigen carriers and in "isolated anti-HBc" antibodies profile in Central Tunisia. *Hepat Mon* 2016; 16: e32354.
EM38. Mohammad Alizadeh AH, Ranjbar M, Tehrani AS, et al. Seroprevalence of hepatitis D virus and its risk factors in the west of Iran. *J Microbiol Immunol Infect* 2010; 43: 519-23.
EM39. Morcos MM, Mikhail TH, Hanna WM, Abdel-Fattah S, el-Rasad MM, Wassef EL. The prevalence of delta virus infection in chronic liver disease in Egyptian children in comparison with some other countries. *Panminerva Med* 2000; 42: 97-100.
EM40. Muhammad SK, Shaikh BA, Gurbakshani KM, Jalbani AU. Frequency triple hepatitis (hepatitis B and C and D virus) in HBs Ag positive patients. *Rawal Med J* 2012; 37: 142-7.
EM41. Nazarnezhad M, Moosavy SH, Dvoodian P, Najaf M, Mahboobi H. Prevalence of hepatitis D virus in hepatitis B virus infected patients referred to Shahid Mohammadi hospital in Bandar Abbas, south of Iran. *Hepatitis Mon* 2017; 17: 81-2.
EM42. Omar M, Khan A, Altaf A, et al. Predictive factors for cirrhosis in chronic hepatitis B mono-infection compared to HBV/ HDV, and HBV/HCV co-infection: Single center experience from Lahore. *Hepatology* 2018; 68.

EM43. Pournik O, Alavian SM, Ghalichi L, Hajibeigi B, Razavi AR, Eslami S. Lower intrafamilial transmission rate of hepatitis B in patients with hepatitis d coinfection: a data-mining approach. *Hepat Mon* 2013; 13: e7652.
EM44. Rahimkhani M, Daneshvar HK. Survay of hepatitis D in cirrhotic patients. *Histopathology* 2012; 61: 156.
EM13. Ramia S, El-Zaatari M, Sharara AI, Ramlawi F, Farhat B. Current prevalence of hepatitis delta virus (HDV) infection and the range of HDV genotypes in Lebanon. *Epidemiol Infect* 2007; 135: 959-62.
EM16. Saudy N, Sugauchi F, Tanaka Y, et al. Genotypes and phylogenetic characterization of hepatitis B and delta viruses in Egypt. *J Med Virol* 2003; 70: 529-36.
EM45. Sayad B, Naderi Y, Alavian SM, et al. Hepatitis D virus infection in Kermanshah, west of Iran: seroprevalence and viremic infections. *Gastroenterol Hepatol Bed Bench* 2018; 11: 145-52.
EM46. Seetlani NK, Abbas Z, Raza S, Yakoob J, Jafris W. Prevalence of hepatitis D in HBsAg positive patients visiting liver clinics. *J Pak Med Assoc* 2009; 59: 434-7.
EM47. Shaikh MA, Shaikh WM, Solangi GA, Shaikh BA, Soomro MA. Frequency of hepatitis D virus infection in hepatitis B surface antigen-positive liver diseases. *J Coll Physicians Surg Pak* 2011; 21: 23-5.
EM48. Sofan M, Rad EG, Farazi AA, et al. Frequency of hepatitis D virus infection in chronic hepatitis B patients in Arak. *Hepat Mon* 2017; 17: 44.
EM49. Somi MH, Farhang S, Miri SM, Pouri AA, Mjidi G, Alavian SM. The frequency of hepatitis D virus in patients with hepatitis B in Iran: an increasing rate? *Trop Doct* 2009; 39: 154-6.
EM50. Taghavi SA, Sedighi S, Mehrabani D, Khademolhosseini F. Hepatitis D in chronic active hepatitis B: Prevalence, liver enzyme levels and histopathology - An epidemiological study in Shiraz, Southern Iran, 2003-2004. *Hepat Mon* 2008; 8: 248-51.
EM19. Ur Rahman S, Hafiz A. Study of hepatitis D in high risk population of hepatitis B. *J Coll Physicians Surgeons Pakistan* 2000; 10: 29-32.
EM51. Yacoubi L, Brichler S, Mansour W, et al. Molecular epidemiology of hepatitis B and Delta virus strains that spread in the Mediterranean North East Coast of Tunisia. *J Clin Virol* 2015; 72: 126-32.
EM52. Zaki SA, Abou Khatwa M, Mikail H. Prevalence of infection with delta virus in patients positive for hepatitis B surface antigen. *Hepatol Intern* 2011; 5: 153.
EM53. Ziaee M, Azarkar G. Prevalence of hepatitis d virus infection among patients with chronic hepatitis B attending birjand hepatitis clinic (East of iran) in 2012. *Hepat Mon* 2013; 13: e11168.
EM54. Zuberi BF, Quraishy MS, Afsar S, Akhtar N, Kumar A, Dodani SK. Treatment outcome in patients of hepatitis B with hepatitis D: experience of 4 years at a tertiary care centre in Pakistan. J Coll Physicians Surg Pak 2007; 17: 320-2.

European Region (EUR)

EU18. Aguilera A, Trastoy R, Rodriguez-Calvino J, Manso T, de Mendoza C, Soriano V. Prevalence and incidence of hepatitis delta in patients with chronic hepatitis B in Spain. *Eur J Gastroenterol Hepatol* 2018; 30: 1060-2.
EU19. Ayaz C, Koruk ST, Yalci A, et al. Epidemiology and risk factors of hepatitis delta infection in Turkey. *J Pure App Microbiol* 2013; 7: 2809-13.
EU20. Bahcecioglu IH, Aygun C, Gozel N, Poyrazoglu OK, Bulut Y, Yalniz M. Prevalence of hepatitis delta virus (HDV) infection in chronic hepatitis B patients in eastern Turkey: still a serious problem to consider. *J Viral Hepat* 2011; 18: 518-24.
EU21. Bayan K, Yilmaz S, Tuzun Y, Yildirim Y. Epidemiological and clinical aspects of liver cirrhosis in adult patients living in Southeastern Anatolia: leading role of HBV in 505 cases. *Hepatogastroenterol* 2007; 54: 2198-202.
EU22. Bielawski KP, Zietkowski D, Charmuszko U, Sikorska K, Stalke P. Hepatitis delta virus infection in chronically HBV-infected patients from northern Poland. *Arch Virol* 2006; 151: 1207-15.
EU1. Bissinger AL, Berg CP. Is the HDV seroprevalence in patients admitted to a university hospital representative for all HBV carriers in Germany? *Infection* 2013; 41: 1031-2.

EU23. Brancaccio G, Nardi A, Madonia S, et al. The present profile of chronic hepatitis B virus infection highlights future challenges: An analysis of the Multicenter Italian MASTER-B cohort. *Dig Liver Dis* 2018.
EU24. Brichler S, Layese R, Roudot-Thoraval F, et al. Epidemiological, clinical, biological and virological factors influencing the occurrence of hepatocellular carcinoma and validation of predictive scores in 317 HBV-related cirrhotic patients. *Hepatology* 2016; 64.
EU25. Byrne R, Childs K, Sadler MD, et al. Active HDV RNA replication is associated with high HBsAg levels and severe liver damage; a role for HBsAg/HBV DNA ratio to identify those most at risk. *Hepatology* 2016; 64.
EU26. Celen MK, Ayaz C, Hosoglu S, Geyik MF, Ulug M. Anti-hepatitis delta virus seroprevalence and risk factors in patients with hepatitis B in Southeast Turkey. *Saudi Med J* 2006; 27: 617-20.
EU27. Chlabicz S, Grzeszczuk A, Lapinski TW, Prokopowicz D, Panasiuk A. Search for hepatitis delta virus (HDV) infection in hepatitis C patients in north-eastern Poland. Comparison with anti-HDV prevalence in chronic hepatitis B. *Eur J Epidemiol* 2003; 18: 559-61.
EU28. Cross TJ, Rizzi P, Horner M, et al. The increasing prevalence of hepatitis delta virus (HDV) infection in South London. *J Med Virol* 2008; 80: 277-82.
EU29. De Giorgio M, Pecere S, Pasulo L, et al. Nuc therapy in HBV-related chronic liver diseases: Any impact on HCC development? *Liver Transplant* 2014; 20.
EU30. De Paschale M, Manco MT, Belvisi L, et al. Epidemiology of hepatitis D virus (HDV) infection in an urban area of northern Italy. *Infection 2*012; 40: 485-91.
EU31. Degertekin H, Sit D. The prevalence of hepatocellular carcinoma in patients with liver cirrhosis in Southeastern Turkey. *Turkish J Gastroenterol* 1998; 9: 217-20.
EU32. Demirturk N, Demirdal T. Inactive hepatitis B surface antigen carriers and intrafamilial tramsmission: results of a 10-year study. *Clin Mol Hepatol* 2014; 20: 56-60.
EU33. El Bouzidi K, Elamin W, Kranzer K, et al. Hepatitis delta virus testing, epidemiology and management: a multicentre cross-sectional study of patients in London. *J Clin Virol* 2015; 66: 33-7.
EU34. Erhardt A, Hoernke M, Heinzel-Pleines U, Sagir A, Gobel T, Haussinger D. Retrospective analysis of chronic hepatitis D in a West German University Clinic over two decades: migratory pattern, prevalence and clinical outcome. *Z Gastroenterol* 2010; 48: 813-7.
EU35. Gaeta GB, Stroffolini T, Chiaramonte M, et al. Chronic hepatitis D: a vanishing Disease? An Italian multicenter study. *Hepatology* 2000; 32: 824-7.
EU36. Gheorghe L, Csiki IE, Iacob S, et al. Hepatitis Delta Virus Infection in Romania: Prevalence and Risk Factors. *J Gastrointestin Liver Dis* 2015; 24: 413-21.
EU37. Grigorescu M, Pascu O, Acalovschi M, Radu C. What is the actual prevalence of the D virus infection in chronic hepatitis and liver cirrhosis in Romania? *Romanian J Gastroenterol* 2003; 12: 179-82.
EU38. Heidrich B, Deterding K, Tillmann HL, Raupach R, Manns MP, Wedemeyer H. Virological and clinical characteristics of delta hepatitis in Central Europe. *J Viral Hepat* 2009; 16: 883-94.
EU39. Inoyatova FI, Abdumadjidova SU. Delta infection among children with chronic viral hepatitis. *Turkish J Gastroenterol* 1998; 9: 21-3.
EU40. Ivanova L, Russev V. Chronic liver diseases and parenterally transmitted hepatitis viruses. *Eur J Inflam* 2007; 5: 1-6.
EU41. Jelen MM, Hosnjak L, Stunf S, et al. Hepatitis D virus infection in Slovenian patients with chronic hepatitis B virus infection: a national prevalence study and literature review. *Acta Dermatovenerol Alp Pannonica Adriat* 2016; 25: 49-53.
EU6. Kalinin AL, Zhavoronok SV, Mikhailov MI, et al. [Viral hepatitis delta in the republic of Belarus]. *Zh Mikrobiol Epidemiol Immunobiol* 1998; 6: 74-7.
EU42. Khan A, Kurbanov F, Tanaka Y, et al. Epidemiological and clinical evaluation of hepatitis B, hepatitis C, and delta hepatitis viruses in Tajikistan. *J Med Virol* 2008; 80: 268-76.

EU43. Kondili LA, Cuko L, Chionne P, et al. Hepatitis B, C and Delta virus infections in Albanian patients with chronic liver disease: evaluation of possible changes during the last 10 years. *Eur J Gastroenterol Hepatol* 2010; 22: 167-71.
EU44. Kose S, Ece G, Gozaydin A, Turken M. Study on seroprevalence of hepatitis delta in a regional hospital in western Turkey. *J Infect Dev Ctries* 2012; 6: 782-5.
EU45. Neudorf-Grauss R, Bujanover Y, Dinari G, et al. Chronic hepatitis B virus in children in Israel: clinical and epidemiological characteristics and response to interferon therapy. *Isr Med Assoc J* 2000; 2: 164-8.
EU46. Niro GA, Gravinese E, Martini E, et al. Clearance of hepatitis B surface antigen in chronic carriers of hepatitis delta antibodies. *Liver* 2001; 21: 254-9.
EU47. Okten A, Demir K, Kaymakoglu S, Cakaloglu Y, Dincer D, Besisik F. Kronik hepatitlerin etyolojik dagilimiEtiologies of chronic hepatitis. *Turkish J Gastroenterol* 1998; 9: 113-5.
EU48. Olivero A, Ciancio A, Caviglia G, et al. Epidemiology and clinical course of hepatitis delta virus infection over 15 years in a liver center in Northern Italy. *J Viral Hep* 2015; 22: 106.
EU49. Ordieres C, Navascues CA, Gonzalez-Dieguez ML, et al. Prevalence and epidemiology of hepatitis D among patients with chronic hepatitis B virus infection: a report from Northern Spain. *Eur J Gastroenterol Hepatol* 2017; 29: 277-83.
EU50. Ozgenc F, Ecevit CO, Erdemir G, Sertoz R, Yagci RV. Prevelance of hepatitis D co-enfection in children with hepatitis B infection: cross-sectional analyses from Western Turkey. *Turkisk J Gastroenterol* 2013; 24: 345-8.
EU51. Ozkaya D, Yuksel E, Mungan G. The evaluation of the patients with hepatitis delta virus infection. *Hepatol Intern* 2014; 8.
EU52. Piccolo P, Lenci I, Telesca C, et al. Patterns of chronic hepatitis B in Central Italy: a cross-sectional study. *Eur J Public Health* 2010; 20: 711-3.
EU53. Pol S. A French cohort of hepatitis B virus (HBV) infected patients. *Hepatology* 2018; 68.
EU54. Popescu GA, Otelea D, Gavriliu LC, et al. Epidemiology of hepatitis D in patients infected with hepatitis B virus in Bucharest: a cross-sectional study. *J Med Virol* 2013; 85: 769-74.
EU55. Reinheimer C, Doerr HW, Berger A. Hepatitis delta: on soft paws across Germany. *Infection* 2012; 40: 621-5.
EU56. Sagnelli E, Coppola N, Scolastico C, et al. Virologic and clinical expressions of reciprocal inhibitory effect of hepatitis B, C, and delta viruses in patients with chronic hepatitis. *Hepatology* 2000; 32: 1106-10.
EU57. Sagnelli E, Stroffolini T, Mele A, et al. Impact of comorbidities on the severity of chronic hepatitis B at presentation. *World J Gastroenterol* 2012; 18: 1616-21.
EU58. Sagnelli E, Stroffolini T, Sagnelli C, et al. Epidemiological and clinical scenario of chronic liver diseases in Italy: Data from a multicenter nationwide survey. *Dig Liver Dis* 2016; 48: 1066-71.
EU59. Semenov AV. [Prevalence of seronegative hepatitis D among patients with chronic viral hepatitis B]. *Zh Mikrobiol Epidemiol Immunobiol* 2012; 6: 106-9.
EU60. Stasi C, Silvestri C, Berni R, et al. Clinical epidemiology of chronic viral hepatitis B: A Tuscany real-word large-scale cohort study. *World J Hepatol* 2018; 10: 409-16.
EU61. Stroffolini T, Almasio PL, Sagnelli E, Mele A, Gaeta GB. Evolving clinical landscape of chronic hepatitis B: A multicenter Italian study. *J Med Virol* 2009; 81: 1999-2006.
EU62. Topdagi O, Okcu N, Bilen N. The frequency of complications and the etiology of disease in patients with liver cirrhosis in erzurum. *Eurasian J Med* 2014; 46: 110-4.
EU63. Torre F, Basso M, Giannini EG, et al. Clinical and virological survey of patients with hepatitis B surface antigen in an Italian region: clinical considerations and disease burden. *J Med Virol* 2009; 81: 1882-6.
EU64. Tulek N, Ertem GT, Erdinc FS, Ozsoy M, Aktepe E. Prevalence of hepatitis delta virus infection in chronic hepatitis B patients: A seroepidemiological investigation from central Anatoli. *Hepatol Int* 2018; 12.

EU65. Turcanu A, Pitel E, Dumbrava VT, et al. Profile of hepatocellular carcinoma in the Republic of Moldova: first-hand information on the presentation, distribution and etiologies. *Romanian J Intern Med* 2018.
EU66. Turkdogan MK, Bozkurt H, Uygan I, et al. Chronic hepatitis delta virus infection in Van region of eastern Turkey. *Turkish J Gastroenterol* 2005; 16: 17-20.
EU67. William Tong CY, Asher R, Toby M, et al. A re-assessment of the epidemiology and patient characteristics of hepatitis D virus infection in inner city London. *J Infect* 2013; 66: 521-7.
EU68. Yalçin K, Yakut M, Değertekin H, et al. Clinical and epidemiological characteristics of hepatocellular carcinoma cases in east and Southeastern region of Turkey: A multicenter retrospective study. *Turkiye Klinikleri J Med Sci* 2013; 33: 806-13.
EU69. Yesmembetov KI, Mukhin NA, Abdurakhmanov DT. Hepatitis delta - Still of major concern in Russia, due to immigration and intravenous drug usage: View from large single center cohort. *J Hepatol* 2012; 56.

Region of the Americas (AMR)

AM16. Barros LM, Gomes-Gouvea MS, Pinho JR, et al. Hepatitis Delta virus genotype 8 infection in Northeast Brazil: inheritance from African slaves? *Virus Res* 2011; 160: 333-9.
AM17. Benias P, Gopal K, Kim J, Feldman DM, Bodenheimer HC, Min AD. Low prevalence of hepatitis B and delta virus infection in an Urban medical center in the United States. *Gastroenterology* 2011; 140.
AM3. Crispim MA, Fraiji NA, Campello SC, Schriefer NA, Stefani MM, Kiesslich D. Molecular epidemiology of hepatitis B and hepatitis delta viruses circulating in the Western Amazon region, North Brazil. *BMC Infect Dis* 2014; 14: 94.
AM18. Santos MD, Gomes-Gouvea MS, Nunes JD, et al. The hepatitis delta genotype 8 in Northeast Brazil: The North Atlantic slave trade as the potential route for infection. *Virus Res* 2016; 224: 6-11.
AM19. Terrault N, Ghany MG, Hoofnagle JH, et al. Seroprevalence and clinical features of hepatitis D virus (HDV) infection in a north american cohort. *Hepatology* 2015; 62.
AM20. Ton ET, Ton JT, Salcedo JMV, Dallacqua DV, Vasconcelos MPA. Prevalencia de hepatite delta nos pacientes HBsAg positivos diagnosticados em 2017 no estado de Rondonia. *Brazil J Infect Dis* 2018; 22: 116-7.

South-East Asia Region (SEAR)

SE7. Chakraborty P, Kailash U, Jain A, et al. Seroprevalence of hepatitis D virus in patients with hepatitis B virus-related liver diseases. *Indian J Med Res* 2005; 122: 254-7.
SE8. Ishaque SM, Azad AK, Saha SK, et al. Aetiology of cirrhosis of liver of different viral origin in Bangladesh. *J Med Teachers Fed* 2000; 5: 3-9.
SE9. Jaiswal SP, Chitnis DS, Artwani KK, Naik G, Jain AK. Prevalence of anti-delta antibodies in central India. *Trop Gastroenterol* 1999; 20: 29-32.
SE10. Jat SL, Gupta N, Kumar T, et al. Prevalence of hepatitis D virus infection among hepatitis B virus-infected individuals in India. *Indian J Gastroenterol* 2015; 34: 164-8.
SE11. Jeong SH, Kim JM, Ahn HJ, et al. [The prevalence and clinical characteristics of hepatitis-delta infection in Korea]. *Korean J Hepatol* 2005; 11: 43-50.
SE12. Kim HS, Kim SJ, Park HW, et al. Prevalence and clinical significance of hepatitis D virus co-infection in patients with chronic hepatitis B in Korea. *J Med Virol* 2011; 83: 1172-7.
SE2. Lusida MI, Surayah, Sakugawa H, et al. Genotype and subtype analyses of hepatitis B virus (HBV) and possible co-infection of HBV and hepatitis C virus (HCV) or hepatitis D virus (HDV) in blood donors, patients with chronic liver disease and patients on hemodialysis in Surabaya, Indonesia. *Microbiol Immunol* 2003; 47: 969-75.
SE13. Menner AS, Kinkel HT, Rieke B, Esser A, Kupper T. Hepatitis b prevalence in upper Dolpo in the mid western Himalaya of Nepal. *Med Sport* 2014; 18: 117.
SE14. Regmi K, Shrestha JK, Kc S, et al. Prevalence of Hepatitis D Among Patients with Hepatitis B Viral Infection Attending a Tertiary Care Centre of Nepal. *J Nepal Med Assoc* 2017; 56: 417-20.

SE15. Saravanan S, Velu V, Kumarasamy N, et al. Seroprevalence of hepatitis delta virus infection among subjects with underlying hepatic diseases in Chennai, southern India. *Trans R Soc Trop Med Hyg* 2008; 102: 793-6.
SE16. Zaki H, Darmstadt GL, Baten A, Ahsan CR, Saha SK. Seroepidemiology of hepatitis B and delta virus infections in Bangladesh. *J Trop Pediatr* 2003; 49: 371-4.

Western Pacific Region (WPR)

WP16. Chen X, Xuan M, Yin Y. [Study of HDV infection in Shandong province]. *Zhonghua Liu Xing Bing Xue Za Zhi* 1998; 19: 138-40.
WP17. Lin HH, Lee SS, Yu ML, et al. Changing hepatitis D virus epidemiology in a hepatitis B virus endemic area with a national vaccination program. *Hepatology* 2015; 61: 1870-9.
WP18. Nakasone H, Sakugawa H, Shokita H, et al. Prevalence and clinical features of hepatitis delta virus infection in the Miyako Islands, Okinawa, Japan. *J Gastroenterol* 1998; 33: 850-4.
WP19. Oyunsuren T, Kurbanov F, Tanaka Y, et al. High frequency of hepatocellular carcinoma in Mongolia; association with mono-, or co-infection with hepatitis C, B, and delta viruses. *J Med Virol* 2006; 78: 1688-95.
WP20. Tsatsralt-Od B, Takahashi M, Nishizawa T, Endo K, Inoue J, Okamoto H. High prevalence of dual or triple infection of hepatitis B, C, and delta viruses among patients with chronic liver disease in Mongolia. *J Med Virol* 2005; 77: 491-9.
WP21. Turner S, O'Sullivan T, Wright P, Stuart K. Delta hepatitis testing, epidemiology and management in a large tertiary hospital in Australia. *J Gastroenterol Hepatol* 2016; 31: 84-5.
WP22. Wang Y, Yu F, Wang L, et al. Hepatitis D virus infections among hepatitis B virus (HBV) infected individuals with or without HIV co-infection: A retrospective cross-sectional study in one hospital in Beijing. *Hepatol Intern* 2017; 11.
WP23. Xu PS, Han SY, Sun CY, Zhao J. [The analysis of hepatitis delta virus infective markers in the hepatitis is B virus infective people]. *Zhonghua Shi Yan He Lin Chuang Bing Du Xue Za Zhi* 2012; 26: 307-9.

Supplementary appendix 6. Included Studies by WHO Region: Selected Population Groups

African Region (AFR)

AF4. Belyhun Y, Liebert UG, Maier M. Clade homogeneity and low rate of delta virus despite hyperendemicity of hepatitis B virus in Ethiopia. Virol J 2017; 14: 176.

AF47. Birguel J, Ndong JG, Aurenche C, et al. Viral markers of hepatitis B, C and D and HB vaccination status of a health care team in a rural district of Cameroon. *Med Trop* 2011; 71: 201-2.

AF6. Coffie PA, Tchounga BK, Bado G, et al. Prevalence of hepatitis B and delta according to HIV-type: a multi-country cross-sectional survey in West Africa. *BMC Infect Dis* 2017; 17: 466.

AF48. Diop-Ndiaye H, Toure-Kane C, Etard JF, et al. Hepatitis B, C seroprevalence and delta viruses in HIV-1 Senegalese patients at HAART initiation (retrospective study). *J Med Virol* 2008; 80: 1332-6.

AF14. Jaquet A, Wandeler G, Nouaman M, et al. Alcohol use, viral hepatitis and liver fibrosis among HIV-positive persons in West Africa: a cross-sectional study. *J Int AIDS Soc* 2017; 19: 21424.

AF49. Ouedraogo HG, Kouanda S, Grosso A, et al. Hepatitis B, C, and D virus and human T-cell leukemia virus types 1 and 2 infections and correlates among men who have sex with men in Ouagadougou, Burkina Faso. *Virol J* 2018; 15: 194.

AF50. Savassi F, Soares C, Pina De Araujo I, Gomes SA, Motta-Castro AR, Lago BV. Hepatitis B, delta and C viruses detection among HIV-infected individuals from Cape Verde. *J Viral Hepat* 2015; 22: 104-5.

Eastern Mediterranean Region (EMR)

EM55. Aghasadeghi MR, Mohraz M, Bahramali G, et al. Frequency and genotype of hepatitis D virus infection in patients infected with HIV and those undergoing hemodialysis. *Hepat Mon* 2013; 13: e7481.

EM56. Bahri F, Kargar Kheirabad A, Ghasemzadeh I, Shoja S, Gouklani H. Hepatitis viruses B and D and Human Immunodeficiency Virus infections in hemodialysis patients in the south of Iran: Prevalence and genotypes. *Hepat Mon* 2016; 16: e32971.

EM57. Masood Z, Roghiye A, Hasan NM. Prevalence of HCV infection in hemodialysis patients of South Khorasan in comparison with HBV, HDV, HTLV I/II, And HIV infection. *Bangladesh J Med Science* 2014; 13: 36-9.

EM58. Motamedifar MP, Taheri MM, Lankarani KBM, et al. The Prevalence and Risk Factors of Hepatitis Delta Virus in HIV/HBV Co-Infected Patients in Shiraz, Iran, 2012. *Iran J Med Sci* 2015; 40: 448-53.

EM59. Njoh J, Zimmo S. Prevalence of antibody to hepatitis D virus among HBsAg-positive drug-dependent patients in Jeddah, Saudi Arabia. *East Afr Med J* 1998; 75: 327-8.

EM13. Ramia S, El-Zaatari M, Sharara AI, Ramlawi F, Farhat B. Current prevalence of hepatitis delta virus (HDV) infection and the range of HDV genotypes in Lebanon. *Epidemiol Infect* 2007; 135: 959-62.

EM60. Saeed Ur R, Hafiz A. Seroprevalence of HDV in hemodialysis and drug addicts in Karachi. *J Coll Physicians Surgeons Pakistan* 2000; 10: 470-2.

EM61. Yadegarynia D, Hatamai H, Roodsari SR, Arab-Mazar Z. Seroprevalence of hepatitis B, C and D viral among hemodialysis patients in Tehran. *Iran J Microbiol* 2017; 9: 195-9.

EM62. Zahedi MJ, Darvish Moghaddam S, Alavian SM, Dalili M. Seroprevalence of hepatitis viruses B, C, D and HIV infection among hemodialysis patients in Kerman province, south-east Iran. *Hepat Mon* 2012; 12: 339-43.

EM52. Zaki SA, Abou Khatwa M, Mikail H. Prevalence of infection with delta virus in patients positive for hepatitis B surface antigen. *Hepatol Intern* 2011; 5: 153.

European Region (EUR)

EU70. Aguilera A, Trastoy R, Barreiro P, et al. Decline and changing profile of hepatitis delta among injection drug users in Spain. *Antivir Ther* 2018; 23: 87-90.

EU71. Arbune M, Georgescu C. Characteristics of Hepatitis B Co-infection and Disease Evolution in HIV-Positive Paediatric Patients in Romania. *Balkan Med J* 2013; 30: 263-7.

EU72. Arribas JR, Gonzalez-Garcia JJ, Lorenzo A, et al. Single (B or C), dual (BC or BD) and triple (BCD) viral hepatitis in HIV-infected patients in Madrid, Spain. *AIDS* 2005; 19: 1361-5.

EU73. Beguelin C, Moradpour D, Sahli R, et al. Hepatitis delta-associated mortality in HIV/HBV-coinfected patients. *J Hepatol* 2017; 66: 297-303.
EU74. Boyd A, Lacombe K, Miailhes P, et al. Longitudinal evaluation of viral interactions in treated HIV-hepatitis B co-infected patients with additional hepatitis C and D virus. *J Viral Hepat* 2010; 17: 65-76.
EU75. Catalani C, Giuffreda G, Baldi L, et al. Prevalence of HBV, HDV, HCV infection and alleged risk factors in the Pistoia (Italy) haemodialysis population. *Italian J Allergy Clin Immunol* 2008; 18: 22-9.
EU76. Childs K, Welz T, Taylor C. Epidemiology and outcomes of hepatitis delta infection in a large, ethnically diverse UK HIV cohort. *HIV Med* 2010; 11: 11.
EU77. Chircu LV, Barbacini IG, Goldoni E, et al. Evolution of liver damage due to hepatitis viruses (HBV, HDV, HCV), HIV and the development of AIDS. *Mediterranean J Infectious Parasitic Dis* 1998; 13: 21-7.
EU78. Fernandez-Montero JV, Vispo E, Barreiro P, et al. Hepatitis delta is a major determinant of liver decompensation events and death in HIV-infected patients. *Clin Infect Dis* 2014; 58: 1549-53.
EU79. Gaeta GB, Milensu S, Cozzi-Lepri A, et al. Prevalence and clinical outcome of hepatitis delta infection in patients with HIV/HBV coinfection. *J Hepatol* 2018; 68.
EU80. Malliori M, Sypsa V, Psichogiou M, et al. A survey of bloodborne viruses and associated risk behaviours in Greek prisons. *Addiction* 1998; 93: 243-51.
EU81. Rehak V, Krekulova L. Hidden microepidemic of hepatitis D in the population of injecting drug users in Prague, Czech Republic. *Hepatol Int* 2013; 7.
EU82. Santana Rodriguez OE, Male Gil ML, HernandezSantana JF, Liminana Canal JM, Martin Sanchez AM. Prevalence of serologic markers of HBV, HDV, HCV and HIV in non-injection drug users compared to injection drug users in Gran Canaria, Spain. *Eur J Epidemiol* 1998; 14: 555-61.
EU83. Soriano V, Grint D, d'Arminio Monforte A, et al. Hepatitis delta in HIV-infected individuals in Europe. *AIDS* 2011; 25: 1987-92.
EU84. Stroffolini T, D'Egidio PF, Aceti A, et al. Hepatitis C virus infection among drug addicts in Italy. *J Med Virol* 2012; 84: 1608-12.
EU14. Voiculescu M, Iliescu L, Ionescu C, et al. A cross-sectional epidemiological study of HBV, HCV, HDV and HEV prevalence in the Subcarpathian and South-Eastern regions of Romania. *J Gastrointestin Liver Dis* 2010; 19: 43-8.
EU85. Zerdali E, Vanli M, Bozkurt M, et al. Hepatitis A, B, C and D seroprevalence among Turkish HIV/AIDS patients. *Hepatol Int* 2018; 12.

Region of the Americas (AMR)

AM21. Alvarado-Esquivel C, Sablon E, Martinez-Garcia S, Estrada-Martinez S. Hepatitis virus and HIV infections in inmates of a state correctional facility in Mexico. *Epidemiol Infect* 2005; 133: 679-85.
AM22. Barton EN, King SD, Douglas LL. The seroprevalence of hepatitis and retroviral infection in Jamaican haemodialysis patients. *West Indian Med J* 1998; 47: 105-7.
AM23. Braga WS, da Costa Castilho M, dos Santos IC, Moura MA, Segurado AC. Low prevalence of hepatitis B virus, hepatitis D virus and hepatitis C virus among patients with human immunodeficiency virus or acquired immunodeficiency syndrome in the Brazilian Amazon basin. *Rev Soc Bras Med Trop* 2006; 39: 519-22.
AM24. Cocozella DR, Albuquerque MM, Borzi S, et al. [Prevalence of hepatic involvement, alcoholism, hepatis B, C and HIV in patients with background history of drug use]. *Acta Gastroenterol Latino Am* 2003; 33: 177-81.
AM25. de Almeida Pereira RA, Mussi AD, de Azevedo e Silva VC, Souto FJ. Hepatitis B Virus infection in HIV-positive population in Brazil: results of a survey in the state of Mato Grosso and a comparative analysis with other regions of Brazil. *BMC Infect Dis* 2006; 6: 34.
AM26. Fainboim H, Gonzalez J, Fassio E, et al. Prevalence of hepatitis viruses in an anti-human immunodeficiency virus-positive population from Argentina. A multicentre study. *J Viral Hepat* 1999; 6: 53-7.

AM27. Freitas SZ, Soares CC, Tanaka TS, et al. Prevalence, risk factors and genotypes of hepatitis B infection among HIV-infected patients in the State of MS, Central Brazil. *Braz J Infect Dis* 2014; 18: 473-80.
AM28. Kucirka LM, Farzadegan H, Feld JJ, et al. Prevalence, correlates, and viral dynamics of hepatitis delta among injection drug users. *J Infect Dis* 2010; 202: 845-52.
AM29. Leon P, Venegas E, Bengoechea L, et al. [Prevalence of infections by hepatitis B, C, D and E viruses in Bolivia]. *Rev Panam Salud Publica* 1999; 5: 144-51.
AM30. Mahale P, Aka PV, Chen X, et al. Hepatitis D viremia among injection drug users in San Francisco. *J Infect Dis* 2018; 217: 1902-6.
AM31. Mendes-Correa MC, Gomes-Gouvea MS, Alvarado-Mora MV, et al. Hepatitis delta in HIV/HBV co-infected patients in Brazil: is it important? *Int J Infect Dis* 2011; 15: e828-32.
AM32. Oliveira ML, Bastos FI, Telles PR, et al. Prevalence and risk factors for HBV, HCV and HDV infections among injecting drug users from Rio de Janeiro, Brazil. *Braz J Med Biol Res* 1999; 32: 1107-14.
AM33. Pierre A, Feldner A, Carvalho Filho RJ, et al. Prevalence of hepatitis delta virus among hemodialysis and renal transplant patients. *Int J Artif Organs* 2018; 41: 171-4.

South-East Asia Region (SEAR)

SE17. Dinesha TR, Boobalan J, Sivamalar S, et al. Occult HBV infection in HIV-infected adults and evaluation of pooled NAT for HBV. *J Viral Hepat* 2018; 25: 718-23.
SE18. Gibney L, Macaluso M, Kirk K, et al. Prevalence of infectious diseases in Bangladeshi women living adjacent to a truck stand: HIV/STD/hepatitis/genital tract infections. *Sex Transm Infect* 2001; 77: 344-50.
SE7. Jaiswal SB, Chitnis DS, Salgia P, Sepaha A. Prevalence of hepatitis viruses among chronic renal failure patients on hemodialysis in central India. *Dial Transplant* 2002; 31: 234-40.
SE2. Lusida MI, Surayah, Sakugawa H, et al. Genotype and subtype analyses of hepatitis B virus (HBV) and possible co-infection of HBV and hepatitis C virus (HCV) or hepatitis D virus (HDV) in blood donors, patients with chronic liver disease and patients on hemodialysis in Surabaya, Indonesia. *Microbiol Immunol* 2003; 47: 969-75.
SE19. Mittal G, Gupta P, Thakuria B, Mukhiya GK, Mittal M. Profile of hepatitis B virus, hepatitis C virus, hepatitis d virus and human immunodeficiency virus infections in hemodialysis patients of a tertiary care hospital in uttarakhand. *J Clin Exp Hepatol* 2013; 3: 24-8.
SE20. Prasetyo AA, Ariapramuda R, Kindi EA, et al. Men having sex with men in Surakarta, Indonesia: demographics, behavioral characteristics and prevalence of blood borne pathogens. *Southeast Asian J Trop Med Pub Health* 2014; 45: 1032-47.
SE21. Prasetyo AA, Dharmawan R, Raharjo I, Hudiyono. Human leukocyte antigen-E alleles are associated with hepatitis C virus, Torque Teno virus, and Toxoplasma vo-infections but are not associated with hepatitis B virus, hepatitis D virus, and GB virus co-infections in human immunodeficiency virus patients. *J Glob Infect Dis* 2016; 8: 75-81.
SE22. Prasetyo AA, Dirgahayu P, Sari Y, Hudiyono H, Kageyama S. Molecular epidemiology of HIV, HBV, HCV, and HTLV-1/2 in drug abuser inmates in central Javan prisons, Indonesia. *J Infect Dev Ctries* 2013; 7: 453-67.
SE4. Theamboonlers A, Hansurabhanon T, Verachai V, Chongsrisawat V, Poovorawan Y. Hepatitis D virus infection in Thailand: HDV genotyping by RT-PCR, RFLP and direct sequencing. *Infection* 2002; 30: 140-4.

Western Pacific Region (WPR)

WP3. Chang SY, Yang CL, Ko WS, et al. Molecular epidemiology of hepatitis D virus infection among injecting drug users with and without human immunodeficiency virus infection in Taiwan. *J Clin Microbiol* 2011; 49: 1083-9.
WP4. Chen F, Zhang J, Guo F, et al. Hepatitis B, C, and D virus infection showing distinct patterns between injection drug users and the general population. *J Gastroenterol Hepatol* 2017; 32: 515-20.

WP24. Chu FY, Chiang SC, Su FH, Chang YY, Cheng SH. Prevalence of human immunodeficiency virus and its association with hepatitis B, C, and D virus infections among incarcerated male substance abusers in Taiwan. *J Med Virol* 2009; 81: 973-8.
WP8. Dunford L, Carr MJ, Dean J, et al. A multicentre molecular analysis of hepatitis B and blood-borne virus coinfections in Viet Nam. *PLoS One* 2012; 7: e39027.
WP25. Hall N, Thuy LN, Diem Tdo T, et al. High prevalence of hepatitis delta virus among persons who inject drugs, Vietnam. *Emerg Infect Dis* 2015; 21: 540-3.
WP26. Hung CC, Wu SM, Lin PH, et al. Increasing incidence of recent hepatitis D virus infection in HIV-infected patients in an area hyperendemic for hepatitis B virus infection. *Clin Infect Dis* 2014; 58: 1625-33.
WP27. Huo TI, Wu JC, Wu SI, et al. Changing seroepidemiology of hepatitis B, C, and D virus infections in high-risk populations. *J Med Virol* 2004; 72: 41-5.
WP28. Kao JH, Chen PJ, Lai MY, Chen DS. Hepatitis D virus genotypes in intravenous drug users in taiwan: decreasing prevalence and lack of correlation with hepatitis B virus genotypes. *J Clin Microbiol* 2002; 40: 3047-9.
WP29. Kemp R, Miller J, Lungley S, Baker M. Injecting behaviours and prevalence of hepatitis B, C and D markers in New Zealand injecting drug user populations. *N Z Med J* 1998; 111: 50-3.
WP17. Lin HH, Lee SS, Yu ML, et al. Changing hepatitis D virus epidemiology in a hepatitis B virus endemic area with a national vaccination program. *Hepatology* 2015; 61: 1870-9.
WP30. Wang LJ, Lin SK, Chiang SC, Su LW, Chen CK. Risk factors for HIV, viral hepatitis, and syphilis among heroin users in northern Taiwan. *Subst Use Misuse* 2013; 48: 89-98.
WP22. Wang Y, Yu F, Wang L, et al. Hepatitis D virus infections among hepatitis B virus (HBV) infected individuals with or without HIV co-infection: A retrospective cross-sectional study in one hospital in Beijing. *Hepatol Int* 2017; 11.

Supplementary appendix 7. Quality Assessment Checklist

1. Are the characteristics of included participants and the study setting described in detail?
Yes: The authors report the total number, age, and gender distribution of the participants, recruitment setting, clinical setting (if relevant), and selected population group (if relevant).
Partially reported: Relevant characteristics not completely reported.
No: None of the relevant characteristics of the participants are reported.

2. Are the eligibility criteria (inclusion and exclusion criteria) for the study explicit and appropriate?
Yes: The eligibility criteria are clearly stated and replicable. A statement on serological testing entry criteria, and any relevant important inclusion or exclusion criteria are clearly reported.
No: The eligibility criteria are not clearly stated.

3. Is the method of recruitment adequately described and appropriate?
Yes: There is a clear statement of the method of selection of participants.
Unclear: The method used to recruit participants is not clearly stated or no information is provided about the method used to recruit participants in the study.
No: The participants were recruited based on other criteria, such as access to intervention or availability of resources, that could lead to a significant selection bias.

4. Is the sample representative of the target population? Consider which of these target populations is represented:
A. General populations (e.g., community samples, antenatal, blood donors).
B. Selected population groups (e.g., injecting drug users, haemodialysis recipients, men who have sex with men, commercial sex workers, people with HCV or HIV).
C. Liver disease populations (e.g., people attending hepatology clinics, patients with cirrhosis or HCC).

Yes: The recruited population represents the underlying source population.
Unclear: It is not clear whether the recruitment population is representative.
No: The recruited population has material differences from the source population; it is not a representative sample, or there are significant inclusion or exclusion criteria that reduce applicability to the target population.

5. Detail any significant non-representative factors
a) Was this a previously known high prevalence community?
b) Was this a selective sub group identified as high risk e.g., sex workers
c) Were there significant exclusions (e.g., criteria based on ALT results or HCV results)

6. Is there significant bias arising from recall for follow up or retrospective data?
Yes: Only a subset of patients returned for analysis; there is incomplete analysis of the eligible participants (<80%).
Partially/unclear: There is incomplete analysis of eligible participants (<100%); or it is unclear whether all eligible individuals were tested.
No: All eligible individuals or a random sample of eligible individuals who consented to participate were tested.

7. Is the HBsAg laboratory method clearly described in the methods section?
Yes: The HBsAg method is reproducible from the description in the methods.
No: The HBsAg method is not adequately described to permit replication.

8. Is the anti-HDV and/or HDV RNA laboratory method clearly described in the methods section?
Yes: The method is reproducible from the description in the methods/referenced article.
No: The method is not adequately described to permit replication.

9. Is the response rate reported?
Yes: The number or proportion of patients who refused to participate is reported.

Unclear: It is not clear from the information provided how many participants who were eligible who did not participate.
No: The number or proportion of patients who did not participate is not reported.

10. Are both competing interest and source of support for the study reported?
Yes: Both competing interest and source of support (financial or other) received for the study are reported, or the absence of any competing interest and source of support is acknowledged.
Partially reported: Only one of these elements is reported.
No: Either there is no information available about competing interests and sources of support, or only one of these elements is reported.

Supplementary appendix 8. Binomial Mixed Model and Parametric Bootstrapping Procedure

A binomial mixed model was developed to estimate the probability of anti-HDV detection among HBsAg-positive people in general and hepatology clinic populations. Here we describe details of the statistical model and how prevalence estimates have been obtained.

Let Y_i denote the number of HBsAg-positive people who tested positive for anti-HDV out of n_i for the i-th survey, for $i = 1, \ldots, n$. We then assumed that, conditionally on independent and identically distributed Gaussian random effects Z_i with mean zero and variance σ^2, the Y_i was mutually independent. Binomial distribution with probability of a positive HDV test p_i, such that

$$log\left\{\frac{p_i}{1-p_i}\right\} = \beta_0 + \beta_1 LD_i + Z_i, \qquad (1)$$

where LD_i is a dummy variable taking value 1 if the i-th survey was conducted in a hepatology clinic and 0 otherwise.

To estimate the unknown regression coefficients (β_0 and β_1) and random effects variance (σ^2), we accounted for data-quality variation as follows:

Let $[X]$ be a shorthand notation for "the distribution of the random variable X." We then defined a weighted likelihood function for $\theta = (\beta_0, \beta_1, \sigma^2)$as

$$L(\theta) = \prod_{i=1}^{n} L_i(\theta)^{w_i}, \qquad (2)$$

where

$$L_i(\theta) = \int_{-\infty}^{+\infty} [Z_i][Y_i \mid Z_i]\, dZ_i \qquad (3)$$

and $w_i = e^{q_i}$, with q_i denoting a quality score obtained via the principal component analysis run using answers to questions detailed in Supplementary appendix 7. Finally, we maximised (2) by approximating the integral in (3) with a Quasi Monte Carlo method.

Let Cdenote the set of surveys falling within a predefined geographical area (e.g., country or WHO region) and for a given target population (general population or hepatology clinic). To predict anti-HDV prevalence among HBsAg-positive people for C, we took $B = 10{,}000$ samples, say $p_i^{(j)}$, from the distribution of $p_i$conditioned on the data, also known as the predictive distribution for p_i, to obtain

$$\hat{p}_C = \frac{1}{B}\sum_{j=1}^{B} \frac{\sum_{i\in C} N_i w_i p_i^{(j)}}{\sum_{i\in C} N_i w_i}, \qquad (4)$$

where $N_i$is the size of the target population for the i-th survey.

To predict anti-HDV prevalence without conditioning on HBsAg-positive status, we modified equation (4) as

$$\hat{p}_C = \frac{1}{B}\sum_{j=1}^{B} \frac{\sum_{i\in C} N_i w_i r_i^{(j)} p_i^{(j)}}{\sum_{i\in C} N_i w_i}, \qquad (5)$$

where $r_i^{(j)}$corresponds the j-th sample drawn from a prior distribution for the probability of carrying HBsAg. The prior distributions were obtained by matching the lower and upper bound of the reported estimate of HBsAg prevalence to the 0.025 and 0.975 quantiles of a Beta distribution.

The number of people with anti-HDV was computed by multiplying the regional HBV prevalence estimate (from the WHO Global Hepatitis Report 2017, (Available at: http://www.whohbsagdashboard.com/#global-strategies) and HBsAg conditional anti-HDV prevalence estimates and multiplying the resulting anti-HDV prevalence with the United National Population Division (UNPD) population estimates for 2018 (World Population Prospects 2019 (Available at: https://population.un.org/wpp/), with countries grouped by WHO regions.

To derive 95% confidence intervals (CIs) we used a parametric bootstrap procedure. From the 95% CIs for the population level HBV prevalence and the HBsAg conditional anti-HDV prevalence estimates, we found the beta distributions for the 2.5^{th} and 97.5^{th} percentiles which matched the 95% CI bounds for HBV prevalence and anti-HDV, conditional on HBsAg prevalence respectively. We generated N=10,000,000 prevalence samples from each distribution and multiplied these. This was used to obtain a distribution of N estimates of the population anti-HDV prevalence.

We then took the empirical 2.5th and 97.5th quantiles of this sample to be the lower and upper bound of the 95% CI for the population level anti-HDV estimate. To take the product of prevalences, we had to assume both prevalence estimates (HBV, HDV conditional on HBsAg) to be independent. As they were obtained from separate datasets, this was a reasonable assumption. Finally, the CI for the number of people with anti-HDV was derived by multiplying the 95% CI for the anti-HDV prevalence with the UNPD 2018 population estimates.

Statistical source code for the binomial mixed model and the parametric bootstrapping procedure are available on request from the authors.

Supplementary appendix 9. Data Sources for Estimations of Population Sizes

Country / Group	Source	URL
Albania	Instituti i Statistikës	http://www.instat.gov.al/
Algeria	Office National des Statistiques	http://www.ons.dz/
Argentina	Instituto Nacional de Estadística y Censos	http://www.indec.gov.ar/
Benin	Institut National de la Statistique	http://www.insae-bj.org/
Bolivia	Instituto Nacional de Estadística	http://www.ine.gob.bo/
Botswana	Central Statistics Office	http://www.cso.gov.bw/
Brazil	Instituto Brasileiro de Geografia e Estatistica	http://www.igbe.gov.br/
Bulgaria	National Statistical Institute	http://www.nsi.bg/
Burkina Faso	Institut National de la Statistique et de la Demographie	http://www.insd.bf/
Cameroon	Bureau Central des Recensements et des Etudes de Population	http://www.bucrep.cm/
Cameroon	Statistics Cameroon	http://www.statistics-cameroon.org/
CAR	Division des Statistiques, des Etudes Economiques et Sociales	http://www.stat-centrafrique.com/
Colombia	Departamento Administrativo Nacional de Estadistica	http://www.dane.gov.co
Cote d'Ivoire	Institut National de la Statistique	http://www.ins.ci/
Czechia	Czech Statistical Office	http://www.czso.cz/
Denmark	Denmark Statistik (StatBank)	http://www.statbank.dk
Egypt	Central Agency for Public Mobilization and Statistics	http://www.capmas.gov.eg/
Ethiopia	Central Statistical Agency	http://www.csa.gov.et/
Europe	Eurostat	https://ec.europa.eu/eurostat/en/
France	Institut National de la Statistique et des Études Économiques	http://www.insee.fr/
Gabon	Direction Générale des Statistiques	http://www.statgabon.ga/
Gambia	Gambia Bureau of Statistics	http://www.gbos.gov.gm
Germany	Statistisches Bundesamt	http://www.destatis.de/
Germany	Statistik Hessen	https://statistik.hessen.de/
Ghana	Ghana Statistical Service	http://www.statsghana.gov.gh/
Global	United Nations/ UNAIDS AIDS Info	http://aidsinfo.unaids.org/
Guinea-Bissau	Instituto Nacional de Estatística	http://www.stat-guinebissau.com/
Iran	Statistical Centre	http://www.amar.org.ir
Iraq	Central Organization for Statistics and Information Technology	http://www.cosit.gov.iq/
Italy	Istituto Nazionale di Statistica	http://www.istat.it
Jamaica	Jamaica Information Service	https://jis.gov.jm/
Kosovo	Kosovo Agency of Statistics	http://www.ask.rks-gov.net/
Latin America	Ethnologue: Languages of the World	http://www.ethnologue.com/
Libya	Bureau of Statistics and Census	http://www.bsc.ly/
Mali	Institut National de la Statistiques	http://www.instat.gov.ml/
Mauritius	Office National de la Statistique	http://www.ons.mr/
Mexico	Instituto Nacional de Estadística y Geografía	http://www.inegi.org.mx/
Republic of Moldova	Departamentul Statistica si Sociologie	http://www.statistica.md/

Mozambique	Instituto Nacional de Estatistica	http://www.ine.gov.mz/
Nigeria	National Bureau of Statistics	http://www.nigerianstat.gov.ng/
Pakistan	Pakistan Bureau of Statistics	http://www.pbs.gov.pk/
Peru	Instituto Nacional de Estadística e Informática	http://www.inei.gob.pe
Romania	Romania National Institute of Statistics	http://www.insse.ro/
Russian Federation	Federal State Statistics Service	http://www.gks.ru/
Saudi Arabia	Central Department of Statistics & Information	http://www.cdsi.gov.sa/
Senegal	Agence Nationale de la Statistique et de la Démographie	http://www.ansd.sn/
South Africa	Statistics South Africa	http://www.statssa.gov.za/
Spain	Instituto Nacional de Estadística	http://www.ine.es/
Tajikistan	State Statistical Committee	http://www.stat.tj/
Togo	Direction Générale de la Statistique et de la Comptabilité Nationale	http://www.stat-togo.org
Tunisia	Institut National de la Statistique	http://www.ins.tn/
Turkey	State Institute of Statistics	http://www.turkstat.gov.tr
Uganda	Uganda Bureau of Statistics	http://www.ubos.org
United Kingdom	Office for National Statistics	https://www.ons.gov.uk/
USA	US Census Bureau	http://www.census.gov/
Uzbekistan	State Committee of Uzbekistan on Statistics	http://www.stat.uz/
Venezuela	Instituto Nacional de Estadística	http://www.pib.socioambiental.org
Yemen	Central Statistical Organisation	http://www.cso-yemen.org/
Country-level population estimates	United Nations Population Division. World Population Prospects: 2019 Revision; Census reports and other statistical publications from national statistical offices; Eurostat: Demographic Statistics; United Nations Statistical Division: Population and Vital Statistics Report; US Census Bureau: International Database; Secretariat of the Pacific Community: Statistics and Demography Programme	https://population.un.org/wpp/; https://ec.europa.eu/eurostat/web/population-demography-migration-projections/data; https://unstats.un.org/unsd/demographic-social/products/vitstats/index.cshtm; https://www.census.gov/programs-surveys/international-programs/about/idb.html; https://prism.spc.int/
PWID	UNAIDS estimates	http://aidsinfo.unaids.org/; Ref[a]
MSM	UNAIDS estimates	http://aidsinfo.unaids.org/
HCV-positive	Polaris Observatory HCV Collaborators	Ref[b]
HIV-positive	UNAIDS estimates	http://aidsinfo.unaids.org/

[a]Degenhardt L, Peacock A, Colledge S, et al. Global prevalence of injecting drug use and sociodemographic characteristics and prevalence of HIV, HBV, and HCV in people who inject drugs: a multistage systematic review. *Lancet Glob Health* 2017; 5: e1192-e1207.
[b]Global prevalence and genotype distribution of hepatitis C virus infection in 2015: a modelling study. *Lancet Gastroenterol Hepatol* 2017; 2: 161-76.

Abbreviations: CAR, Central African Republic; USA, United States of America; PWID, People who inject drugs; MSM, Men who have sex with men; HCV, Hepatitis C virus.

Supplementary appendix 10. Included Populations by WHO Region

	AFR	EMR	EUR	AMR	SEAR	WPR	Total
Overall population[a], millions	1,0152.8	715.4	928.5	1,006.5	1,982,2	1,945,7	7,631.1
**General population**							
Population represented[b], millions	158.9	308.7	313.2	360.0	148.4	236.9	1,526.1
Number tested for anti-HDV	7,940	2,455	6,924	417	170	6,118	24,024
Community survey	4,400	801	259	210	29	6,043	11,742
Blood donors	1,244	1,645	5,545	207	141	30	8,812
Antenatal clinic	1,098	0	1,118	0	0	0	2,216
HIV clinic[c]	1,088	0	0	0	0	0	1,088
Student / occupational setting	110	9	2	0	0	45	166
**Hepatology clinic populations**							
Population represented[b], millions	96.4	365.0	527.2	19.5	304.0	245.5	1,557.6
Number tested for anti-HDV	3,526	15,194	58,958	1,951	2,364	6,208	88,201
**Selected populations groups[d]**							
Population represented[b], millions	1.7	1.2	6.9	7.2	1.0	0.9	19.1
Number tested for anti-HDV	314	423	2,870	813	216	3,432	8,068
People who inject drugs	0	112	713	166	67	1,696	2,754
Haemodialysis recipients	17	105	19	119	121	41	422
Men who have sex with men	68	0	29	0	14	0	111
Commercial sex workers	0	0	0	1	14	424	439
HCV-positive	0	19	891	257	0	432	1,599
HIV-positive	229	187	1,218	270	0	839	2,743

[a]United Nations Population Division 2018 population estimates (World Population Prospects 2019).
[b]Refers to the sum of the catchment populations represented by included studies, excluding overlapping or repeated samples. For hepatology clinic populations, this refers to the catchment area of the clinical service. For selected population groups, this was based on estimates of the size of the population at risk.
[c]Included in general populations in countries with adult HIV prevalence >1%.
[d]Data from selected population groups partially overlap with hepatology clinic and/or general population samples.

Abbreviations: AFR, African Region; EMR, Eastern Mediterranean Region; EUR, European Region; AMR, Region of the Americas; SEAR, South-East Asian Region; WPR, Western Pacific Region; HCV, Hepatitis C virus.

Supplementary appendix 11. Isolated Populations Tested for Anti-HDV

Country Reference	Population and Region	Year	Tested for HBsAg Number	HBsAg prevalence % (95% CI)	HBsAg-positive tested for anti-HDV Number	Anti-HDV prevalence % (95% CI)
Bolivia AM13	Yurakare, Yuki and Trinitario ethnicities, Bolivian Amazonia	1996	366	1.6 (0.8, 3.5)	6	16.7 (3.0, 56.4)
Bolivia AM13	Rural Andean Highlands	1996	187	0.5 (0.1, 3.0)	1	0.0 (0.0, 79.3)
Brazil AM7	Jurua, Purus and Wood Valleys, Amazon State	<2001	688	9.7 (7.7, 12.2)	67	13.4 (7.2, 23.6)
Brazil AM8	Labrea, South West Amazonas	2005-2006	787	11.8 (9.7, 14.3)	93	41.9 (32.4, 52.1)
Brazil AM9	Acre and Purus Rivers, States of Acre and Amazonas	1997	349	5.2 (3.3, 8.0)	18	66.7 (43.7, 83.7)
Brazil AM14	Parakana Indians Apakterewa and Xingu villages	1995	196	5.6 (3.2, 9.8)	11	0.0 (0.0, 25.9)
		2003-2005	258	3.9 (2.1, 7.0)	10	0.0 (0.0, 27.8)
Brazil AM15	Acre State	2002	2656	3.4 (2.7, 4.1)	89	21.3 (14.1, 31.0)
Brazil AM6	Eastern Amazon	2011-2017	603	0.5 (0.1, 1.5)	3	0.0 (0.0, 56.1)
Colombia AM10	Amazonas State	2011-2013	862	2.7 (1.8, 4.0)	23	26.1 (12.5, 46.5)
Venezuela AM11	Piaroan communities	2002-2004	412	5.1 (3.4, 7.7)	21	19.0 (7.7, 40.0)
Venezuela AM11	Yanomami communities	2002-2004	231	14.3 (10.4, 19.4)	33	6.1 (1.7, 19.6)
USA AM12	Alaska Native Population, Bristol Bay	1993-1994	603	8.8 (6.8, 11.3)	53	0.0 (0.0, 6.8)
Denmark EU15	Itilleq, Greenland	2005-2007	115	27.0 (19.7, 35.7)	31	51.6 (34.8, 68.0)
Denmark EU15	Sarfannguaq, Greenland	2005-2007	72	5.6 (2.2, 13.4)	4	0.0 (0.0, 49.0)
Denmark EU17	West Greenland	<2012	153	2.6 (1.0, 6.5)	4	0.0 (0.0, 49.0)
Denmark EU17	East Greenland	<2012	288	28.8 (23.9, 34.3)	483	1.2 (0.2, 6.5)
Greece EU16	Archangelos, Rhodes	1997	1938	11.5 (10.2, 13.0)	223	25.1 (19.9, 31.2)
Greece EU16	Archangelos, Rhodes	2013	1076	3.3 (2.3, 4.5)	35	20.0 (10.0, 35.9)
India SE6	Andaman and Nicobar Islands	2002	NS	NS	223	3.6 (1.8, 6.9)
India SE5	Upper Dibang Valley, Uttar Pradesh	2005	NS	NS	93	35.5 (26.5, 45.6)

Abbreviations: HBsAg, Hepatitis B surface antigen; CI, Confidence interval; USA, The United States of America; NS, Not stated

Supplementary appendix 12. List of anti-HDV assays used in included studies

Method and Manufacturer[a]	N	%
ELISA, Abbott, USA	71	18.0
ELISA, ETI-AB-DELTAK-2, Diasorin, Italy	66	16.8
Method not specified	51	12.9
ELISA, Dia.Pro Diagnostic Bioprobes, Italy	50	12.7
ELISA, Diasorin, Italy	39	9.9
ELISA, manufacturer not specified	39	9.9
ELISA, Murex anti-delta, Wellcozyme, United Kingdom	9	2.3
ELISA, Radim Diagnostics, Germany	8	2.0
ELISA, Diagnostic Automation, USA	6	1.5
ELISA, Wantai, China	6	1.5
Architect chemiluminescence assay, Abbott, USA	4	1.0
ELISA, In-house assay	4	1.0
RIA, Abbott, USA	4	1.0
ELISA, DRG Diagnostics, Germany	3	0.8
ELISA, Hepanostika, Biomeriux, France	3	0.8
ELISA, Biovendor, Germany	3	0.8
ELISA, Biokit, Spain	2	0.5
RIA, Diasorin, Italy	2	0.5
ELISA, Globe Diagnostics, Italy	2	0.5
ELISA, Institute of Chinese Academy of Preventive Medicine, China	2	0.5
ELISA, RPC Diagnostics Systems, Russian Federation	2	0.5
ELISA, Sorin Biomedica, Italy	2	0.5
Western blot	2	0.5
ELISA, MBS, Italy	1	0.3
ELISA, Beijing Bell Bioengineering, China	1	0.3
Cobas e601 chemilumiscence assay, Roche, Switzerland	1	0.3
ELISA, Creative Diagnostics, USA	1	0.3
ELISA, Deltassay, Sanofi Pasteur Diagnostic, France	1	0.3
ELISA, Detect, Biochem Immunosystems, Italy	1	0.3
ELISA, Equipar, Italy	1	0.3
ELISA, Genway Biotech, USA	1	0.3
ELISA, InGen, France	1	0.3
ELISA, Ortho Diagnostics, USA	1	0.3
RIA, manufacturer not specified	1	0.3
RIA, Dianabot, Japan	1	0.3
ELISA, SmarTest Diagnostics, Israel	1	0.3
ELISA, TKA 4HD, Teknolabo, Italy	1	0.3

[a]Some studies used more than one assay.

Abbreviations: ELISA Enzyme linked immunosorbent assay; USA, The United States of America; RIA Radioimmunoassay.

Supplementary appendix 13. Country-Level Anti-HDV Prevalence Estimates in General Populations (HBsAg-Positive and Total) and Hepatology Clinic Populations (HBsAg-Positive)

WHO Region	Country	General Populations % (95% CI)		Hepatology Clinic Populations % (95% CI)
		HBsAg-positive	Total	HBsAg-positive
AFR	Algeria	-	-	5.6 (2.2, 10.3)
AFR	Benin	10.5 (3.7, 20.4)	1.7 (0.6, 3.4)	-
AFR	Botswana	3.7 (0.2, 15.6)	0.1 (0.01, 0.7)	-
AFR	Burkina Faso	1.7 (1.1, 2.4)	0.2 (0.1, 0.2)	3.5 (0.2, 13.3)
AFR	Cameroon	11.1 (8.9, 14.9)	1.0 (0.9, 1.2)	28.7 (22.2, 35.4)
AFR	CAR	11.2 (6.9, 16.2)	0.9 (0.6, 1.4)	43.6 (21.2, 67.7)
AFR	Cote d'Ivoire	14.7 (6.2, 26.0)	1.2 (0.5, 2.3)	-
AFR	Ethiopia	3.4 (0.9, 7.5)	0.3 (0.1, 0.6)	9.7 (5.6, 14.8)
AFR	Gabon	22.0 (16.1, 29.2)	2.0 (1.5, 2.8)	-
AFR	Gambia	1.4 (0.7, 2.3)	0.1 (0.05, 0.1)	11.5 (7.4, 16.3)
AFR	Ghana	5.6 (3.1, 8.9)	0.5 (0.3, 0.8)	11.8 (6.3, 18.8)
AFR	Guinea-Bissau	23.9 (15.1, 34.2)	3.9 (2.4, 5.9)	-
AFR	Malawi	1.8 (0.3, 4.6)	0.2 (0.05, 0.5)	-
AFR	Mali	6.5 (0.2, 31.9)	0.8 (0.02, 3.9)	-
AFR	Mauritania	19.4 (16.1, 22.9)	2.9 (2.4, 3.5)	32.3 (27.1, 37.5)
AFR	Mozambique	0.7 (0.1, 2.2)	0.03 (0.003, 0.1)	-
AFR	Nigeria	10.6 (5.1, 18.0)	1.6 (0.7, 3.1)	4.7 (3.2, 7.8)
AFR	Senegal	3.0 (1.1, 5.8)	0.2 (0.1, 0.5)	13.8 (5.6, 26)
AFR	South Africa	1.6 (0.4, 4.0)	0.1 (0.02, 0.2)	-
AFR	United Republic of Tanzania	5.0 (2.6, 8.3)	0.2 (0.1, 0.4)	-
AFR	Togo	18.5 (4.5, 40.0)	1.1 (0.2, 2.6)	14.0 (5.7, 26.5)
AFR	Uganda	3.3 (1.3, 6.1)	0.2 (0.1, 0.4)	-
EMR	Egypt	3.8 (1.9, 6.6)	0.1 (0.03, 0.1)	7.4 (2.6, 16.2)
EMR	Iran	3.2 (1.9, 5.3)	0.1 (0.04, 0.1)	11.5 (9.7, 13.3)
EMR	Iraq	3.7 (0.1, 16.1)	0.2 (0.01, 0.8)	-
EMR	Lebanon	1.4 (0.2, 4.1)	0.02 (0.003, 0.05)	3.0 (0.9, 7.0)
EMR	Libya	-	-	2.6 (1.2, 4.6)
EMR	Pakistan	9.0 (1.6, 22.7)	0.6 (0.1, 1.7)	20.5 (11.8, 33.2)
EMR	Saudi Arabia	3.6 (0.7, 8.9)	0.1 (0.01, 0.2)	6.7 (4.5, 9.6)
EMR	Tunisia	4.7 (2.1, 8.1)	0.2 (0.1, 0.4)	5.7 (2.5, 12.4)
EMR	Yemen	2.9 (0.1, 11.8)	0.1 (0.004, 0.4)	-
EUR	Albania	-	-	10.6 (6.4, 15.7)
EUR	Belarus	3.4 (1.8, 5.4)	0.1 (0.05, 0.1)	21.7 (15.5, 28.5)
EUR	Bulgaria	-	-	10.7 (6.0, 16.2)
EUR	France	2.0 (1.7, 2.4)	0.01 (0.004, 0.01)	4.7 (4.1, 5.4)
EUR	Germany	2.6 (1.1, 4.8)	0.02 (0.01, 0.03)	6.6 (5.9, 7.3)
EUR	Israel	-	-	9.7 (3.5, 18.5)
EUR	Italy	3.2 (0.2, 13.2)	0.02 (0, 0.1)	9.3 (8.4, 10.2)
EUR	Kosovo	4.0 (0.4, 12.1)	0.1 (0.01, 0.3)	-
EUR	Republic of Moldova	15.0 (9.4, 22.6)	1.3 (0.8, 1.9)	46.4 (33.5, 59.5)
EUR	Poland	-	-	5.1 (1.6, 10.6)

EUR	Romania	7.0 (1.0, 27.2)	0.4 (0.1, 1.5)	32.0 (29.7, 34.3)
EUR	Russian Federation	-	-	19.7 (16.5, 23.0)
EUR	Slovenia	-	-	0.4 (0.1, 0.7)
EUR	Spain	-	-	10.8 (8.4, 13.5)
EUR	Tajikistan	-	-	24.0 (13.1, 36.3)
EUR	Turkey	3.1 (1.5, 5.4)	0.1 (0.1, 0.2)	32.7 (26.8, 39.1)
EUR	United Kingdom	2.1 (0.9, 3.9)	0.02 (0.001, 0.1)	5.3 (4.7, 6.0)
EUR	Uzbekistan	-	-	17.3 (10.4, 25.6)
AMR	Argentina	2.7 (0.5, 7.6)	0.004 (0.001, 0.01)	-
AMR	Brazil	7.2 (1.1, 30.5)	0.05 (0.01, 0.2)	11.0 (8.7, 13.7)
AMR	Canada	-	-	2.6 (1.8, 3.6)
AMR	Peru	5.7 (0.2, 26.1)	0.1 (0.001, 0.3)	-
AMR	USA	5.9 (3.0, 9.8)	0.02 (0.01, 0.03)	2.6 (1.8, 3.6)
SEAR	Bangladesh	4.1 (0.2, 17.5)	0.3 (0.01, 1.4)	15.4 (11.3, 20.6)
SEAR	India	-	-	4.9 (3.5, 6.6)
SEAR	Indonesia	0.8 (0.1, 2.8)	0.02 (0.002, 0.07)	0.7 (0.1, 2.0)
SEAR	Nepal	-	-	2.0 (0.1, 6.8)
SEAR	Republic of Korea	-	-	2.0 (1.2, 3.1)
SEAR	Thailand	1.6 (0.1, 6.1)	0.02 (0.002, 0.1)	-
WPR	Australia	1.3 (0.1, 4.5)	0.01 (0.0, 0.02)	4.4 (3.1, 6.0)
WPR	China	2.1 (1.6, 2.7)	0.4 (0.3, 0.5)	4.5 (4.0, 5.1)
WPR	Japan	8.5 (5.9, 11.4)	0.7 (0.5, 0.9)	21.3 (17.4, 25.5)
WPR	Mongolia	36.9 (31.3,43.1)	4.0 (3.2, 4.8)	74.7 (69.3, 79.8)
WPR	Vietnam	10.8 (5.2, 18.2)	1.4 (0.7, 2.3)	-

Abbreviations: HBsAg, Hepatitis B surface antigen; CI, confidence interval; AFR, African Region; EMR, Eastern Mediterranean Region; EUR, European Region; AMR, Region of the Americas; SEAR, South-East Asian Region; WPR, Western Pacific Region; CAR, Central African Republic; USA, The United States of America

Supplementary appendix 14. Forest Plot of Anti-HDV Prevalence Among People Who Inject Drugs Relative to Comparator Populations[a]

Reference	Country	Year(s) of study	Control Reference	Control Type	Odds ratio (95% CI)	% Weight
**Internal General / Selected Population Controls**						
EU83	Europe	1994 – 2011		HIV	12.98 (6.95, 24.25)	3.96
EU73	Switzerland	1988–2014		HIV	15.26 (9.46, 24.62)	4.09
AM26	Argentina	1994–95		HIV	4.97 (0.58, 42.39)	2.12
SE4	Thailand	<2001		CO	20.98 (1.20, 366.56)	1.52
WP3	China	2006–09		CO	84.67 (22.21, 322.78)	3.07
WP3	China	2006–09		CO	129.89 (39.34, 428.81)	3.26
WP4	China	2014		CO	19.25 (4.61, 80.44)	2.95
Subtotal (I² = 69.3%, τ² = 0.58)					24.84 (11.75, 52.50)	20.98
**Internal Hepatology Clinic Controls**						
EM50	Iran	2003–04		HEP	7.11 (1.26, 40.21)	2.57
EU35	Italy	1997		HEP	10.16 (3.02, 34.26)	3.23
EU30	Italy	2007–08		HEP	26.89 (9.08, 79.59)	3.41
EU36	Romania	2011		HEP	1.48 (0.64, 3.43)	3.72
EU49	Spain	1998–12		HEP	110.73 (61.91, 198.05)	4.00
EU67	United Kingdom	2008–12		HEP	5.04 (1.48, 17.16)	3.22
Subtotal (I–squared = 93.8%, τ²= 3.88)					11.05 (2.13, 57.28	20.15
**External General Population Controls**						
EM52	Egypt	<2011	EM9	CO	18.85 (0.80, 442.93)	1.33
EM60	Pakistan	1995	EM19	BD	2.70 (0.38, 18.96)	2.33
EM59	Saudi Arabia	1996–97	EM5	BD	4.56 (0.97, 21.39)	2.80
EU77	Italy	1986–1997	EU3	CO	22.45 (1.30, 387.10)	1.53
EU84	Italy	2009	EU3	CO	7.00 (0.33, 150.06)	1.39
AM24	Argentina	NS	AM4	BD	155.00 (4.95, 4854.73)	1.18
AM28	USA	2005–06	AM5	CO	15.80 (6.41, 38.92)	3.64
AM30	USA	NS	AM5	CO	8.74 (3.93, 19.43)	3.77
WP27	China	1985	WP10	CO	172.28 (55.74, 532.49)	3.35
WP28	China	1997	WP10	CO	29.53 (21.50, 40.56)	4.21
WP27	China	2001–02	WP10	CO	7.35 (3.82, 14.14)	3.93
WP17	China	2001–12	WP10	CO	35.95 (24.35, 53.10)	4.16
WP17	China	2001–12	WP10	CO	137.13 (95.02, 197.91)	4.18
WP24	China	2005	WP10	CO	27.57 (12.96, 58.61)	3.82
WP30	China	2006	WP10	CO	33.41 (13.03, 85.66)	3.59
WP25	Vietnam	2010–11	WP14	CO	11.21 (2.26, 55.76)	2.73
WP25	Vietnam	2010–11	WP14	CO	6.93 (1.36, 35.19)	2.70
WP25	Vietnam	2010–11	WP14	CO	4.36 (0.78, 24.36)	2.59
WP25	Vietnam	2010–11	WP14	CO	34.02 (7.30, 158.53)	2.81
WP25	Vietnam	2010–11	WP14	CO	32.71 (7.16, 149.43)	2.84
Subtotal (I–squared = 85.2%, τ² = 0.96)					20.70 (12.17, 35.21)	58.87
Overall (I–squared = 86.7%, τ² = 1.16)					19.00 (12.26, 29.45)	100.00

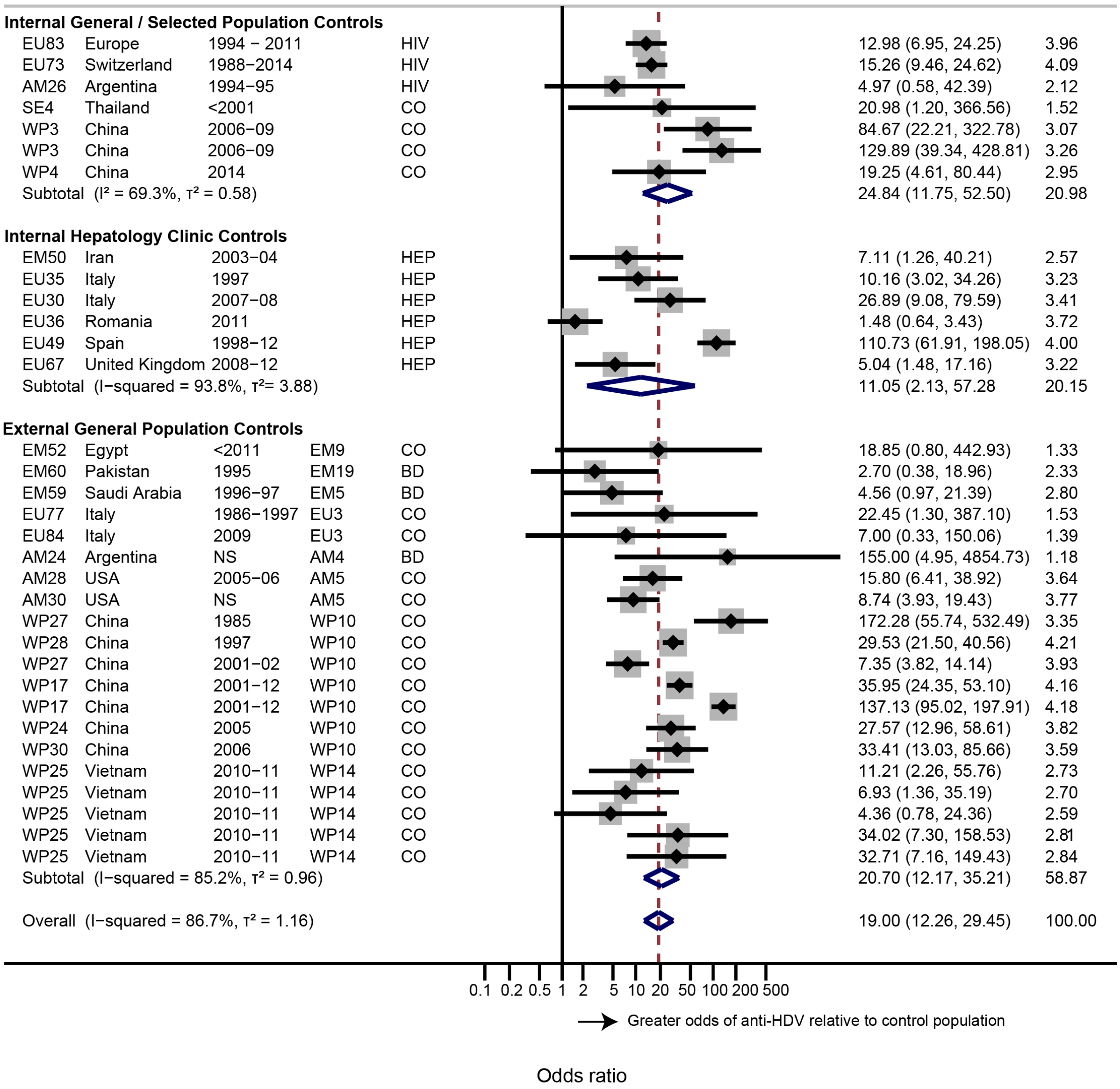

[a]Comparator populations: HIV clinic attendees (HIV); Community (CO), Hepatology clinic population (HEP), Blood donors (BD); [b]Europe includes 34 European countries and Argentina.

Abbreviations: CI, confidence interval; USA, The United States of America.

Supplementary appendix 15. Forest Plot of Anti-HDV Prevalence Among Haemodialysis Recipients Relative to Comparator Populations[a]

Reference	Country	Year(s) of study	Control Reference	Control Type	Odds ratio (95% CI)	% Weight
**Internal General Population Controls**						
EM13	Lebanon	2002–04		BD	1.73 (0.07, 44.01)	6.69
**Internal Hepatology Clinic Controls**						
SE7	India	1992–2000		ASY	0.49 (0.02, 12.49)	6.67
**External General Population Controls**						
EM52	Egypt	<2011	EM9	CO	5.98 (0.27, 131.66)	7.22
EM56	Iran	2015	EM6	BD	2.38 (0.13, 42.43)	8.12
EM62	Iran	2010	EM6	BD	1.37 (0.08, 23.71)	8.26
EM57	Iran	2010	EM17	CO	6.26 (0.22, 176.30)	6.34
EM61	Iran	2016	EM6	BD	4.11 (0.22, 77.10)	7.89
EM55	Iran	<2013	EM6	BD	23.22 (5.38, 100.24)	20.62
EM60	Pakistan	1995	EM19	BD	0.47 (0.04, 5.69)	10.26
WP8	Vietnam	2008–11	WP14	CO	1.96 (0.17, 22.19)	10.65
Subtotal (I^2 = 33.2%, τ^2 = 0.82)					3.76 (1.24, 11.37)	79.37
**External Hepatology Clinic Controls**						
EU75	Italy	2007	EU57	ASY	7.16 (0.33, 155.86)	7.27
Overall (I^2 = 21.0%, τ^2 = 0.49)					3.42 (1.38, 8.48)	100.00

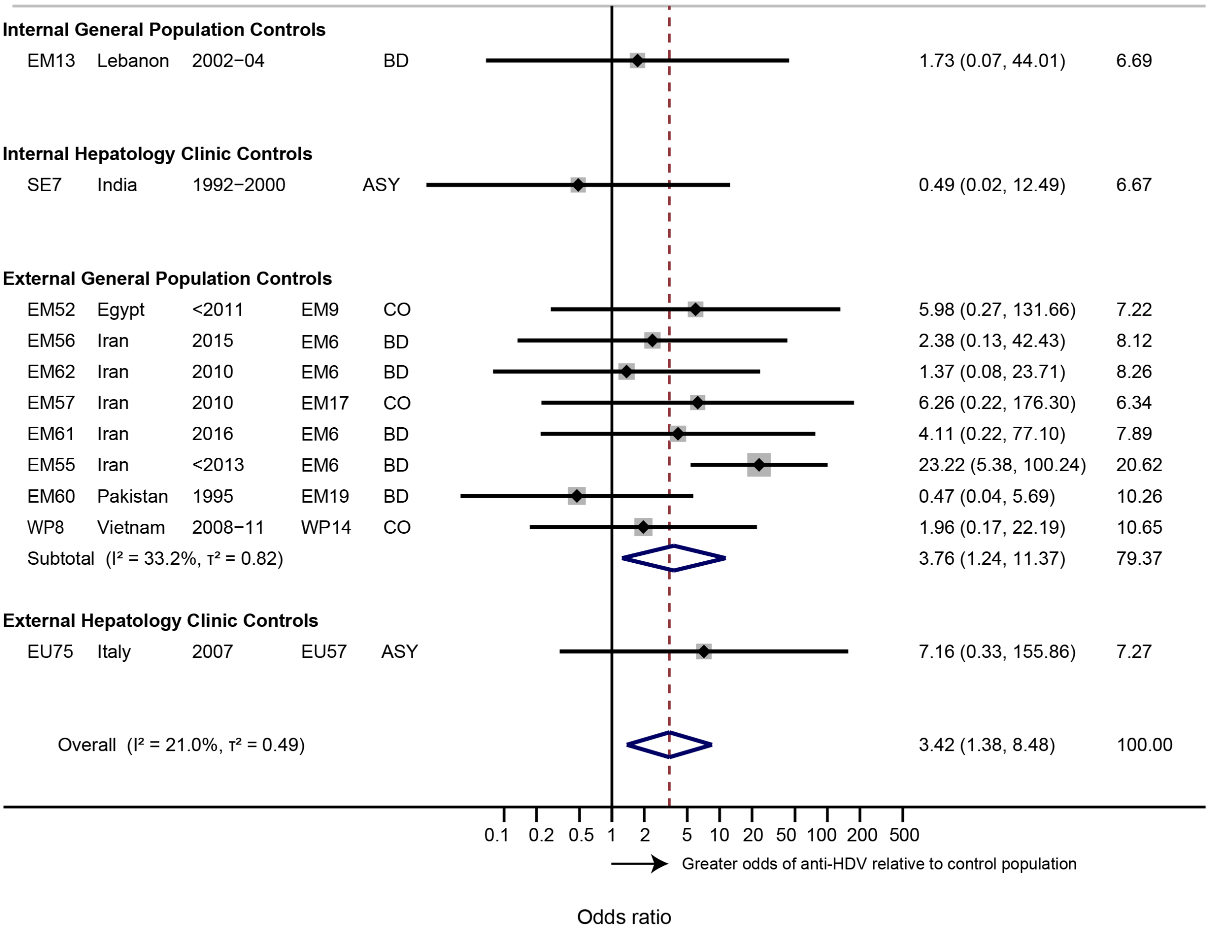

[a]Comparator populations: Blood donors (BD), Asymptomatic HBsAg-positive people (ASY), Community (CO).

Abbreviations: CI, confidence interval.

Supplementary appendix 16. Forest Plot of Anti-HDV Prevalence among Commercial Sex Workers Relative to Comparator Populations[a]

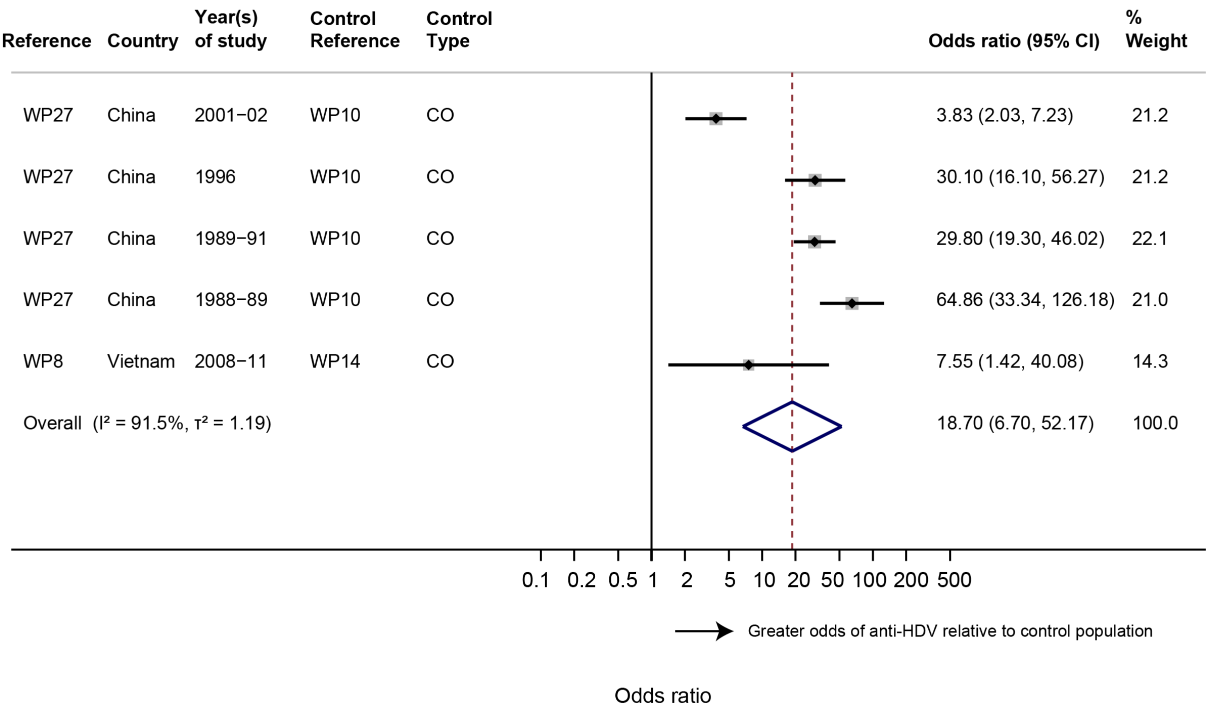

[a]Comparator populations: All were external community (CO) populations from the same geographic region.

Abbreviations: CI, confidence interval.

Supplementary appendix 17. Forest Plot of Anti-HDV Prevalence Among Men Who Have Sex with Men Relative to Comparator Populations[a]

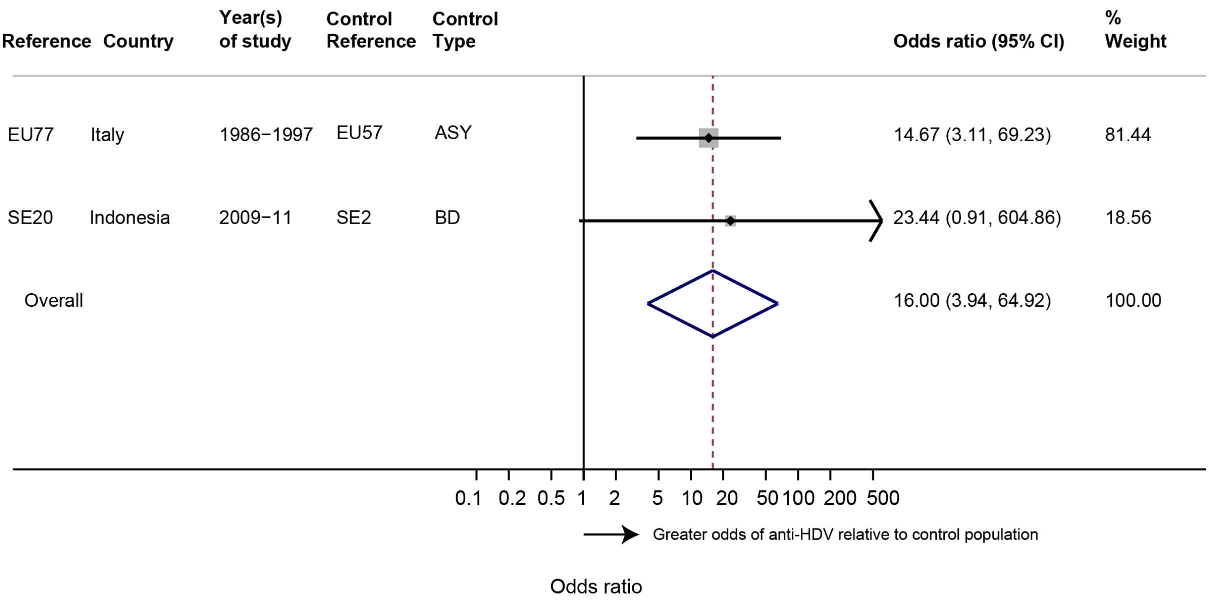

[a]Comparator populations: Asymptomatic HBsAg-positive people (ASY), Blood donors (BD).

Abbreviations: CI, confidence interval.

Supplementary appendix 18. Forest Plot of Anti-HDV Prevalence Among Hepatitis C Virus (HCV)-Antibody Positive People Relative to Comparator Populations[a]

Reference	Country	Year(s) of study	Population Type	Odds ratio (95% CI)	% Weight
**Internal General Population Controls**					
EU74	France	2010	HIV	13.95 (5.53, 35.20)	6.52
EU77	Italy	1998	PWID	2.22 (1.20, 4.10)	7.14
EU83	Europe	2011	HIV	8.96 (4.89, 16.42)	7.16
EU72	Spain	2005	HIV	60.00 (6.01, 599.49)	3.62
EU73	Switzerland	2017	HIV	184.18 (44.57, 761.07)	5.39
AM28	USA	2010	PWID	1443.00 (27.20, 76548.96)	1.76
AM5	USA	2016	CO	4.25 (0.43, 42.06)	3.63
WP17	China (Taiwan)	2015	CO	25.15 (18.48, 34.24)	7.57
WP26	China (Taiwan)	2014	HIV	5.49 (2.61, 11.51)	6.91
Subtotal (I² = 89.7%, τ²= 1.36)				15.65 (6.41, 38.19)	49.71
**Internal Hepatology Clinic Controls**					
EM51	Tunisia	2015	HEP	1.92 (0.42, 8.67)	5.19
EU38	Germany	2009	HEP	5.30 (3.82, 7.36)	7.55
EU18	Spain	2018	HEP	43.04 (14.94, 123.96)	6.23
EU49	Spain	2017	HEP	34.36 (20.86, 56.60)	7.33
EU28	UK	2008	HEP	8.84 (4.82, 16.19)	7.16
EU67	UK	2013	HEP	6.07 (2.01, 18.30)	6.12
AM19	USA and Canada	2015	HEP	2.98 (0.38, 23.52)	4.03
WP20	Mongolia	2005	HEP	0.77 (0.33, 1.81)	6.69
Subtotal (I−squared = 91.6%, τ²= 1.41)				6.52 (2.64, 16.06)	50.29
Overall (I−squared = 90.7%, τ²= 1.22)				10.02 (5.49, 18.26)	100.00

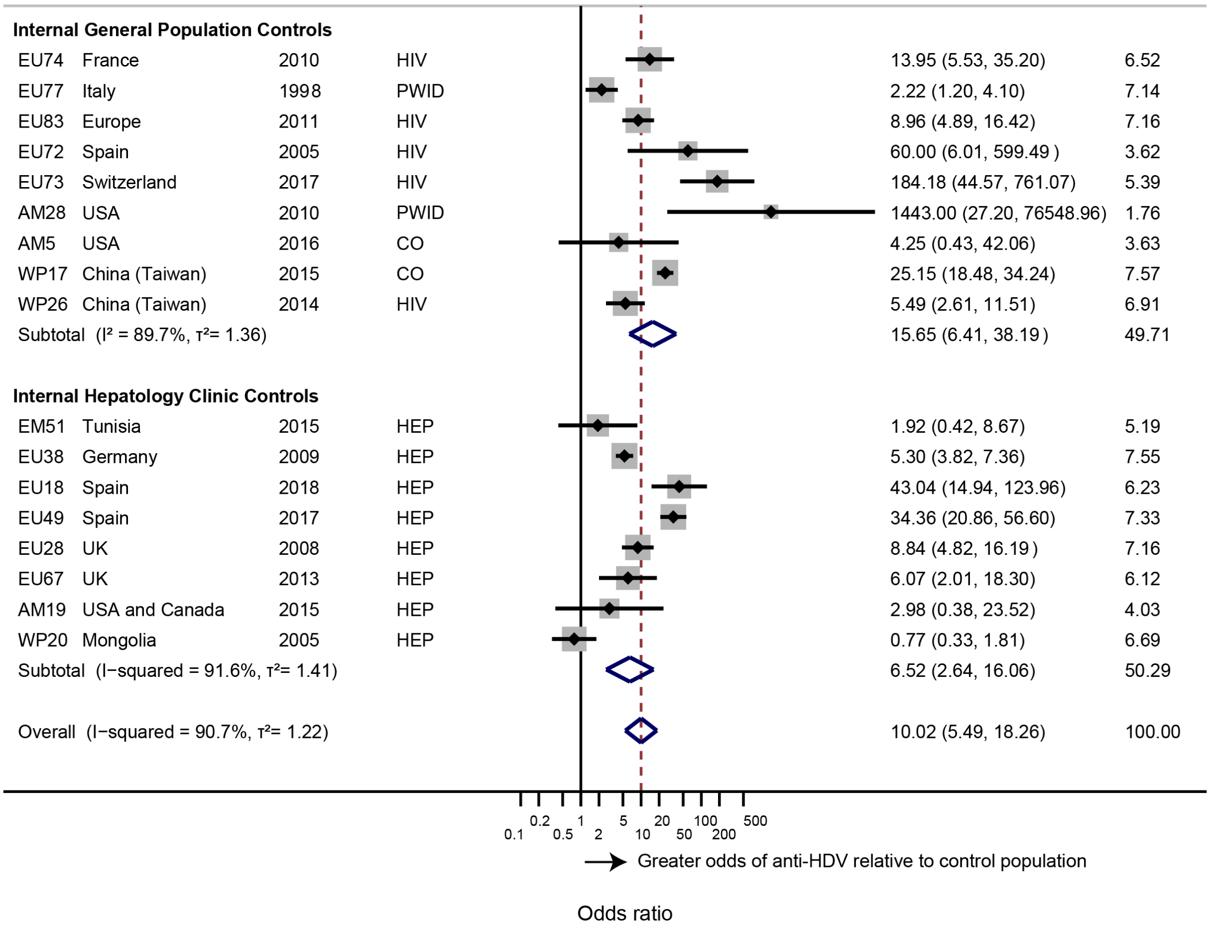

[a]Comparator populations: HIV clinic attendees (HIV), people who inject drugs (PWID), Community (CO), Hepatology clinic attendees (all, analysed by HCV status) (HEP); [b]Europe includes 34 European countries and Argentina.

Abbreviations: CI, confidence interval; USA, The United States of America.

Supplementary appendix 19. Forest Plot of Anti-HDV Prevalence Among People with HIV Relative to Comparator Populations[a] in Countries Without Generalised HIV Epidemics[b]

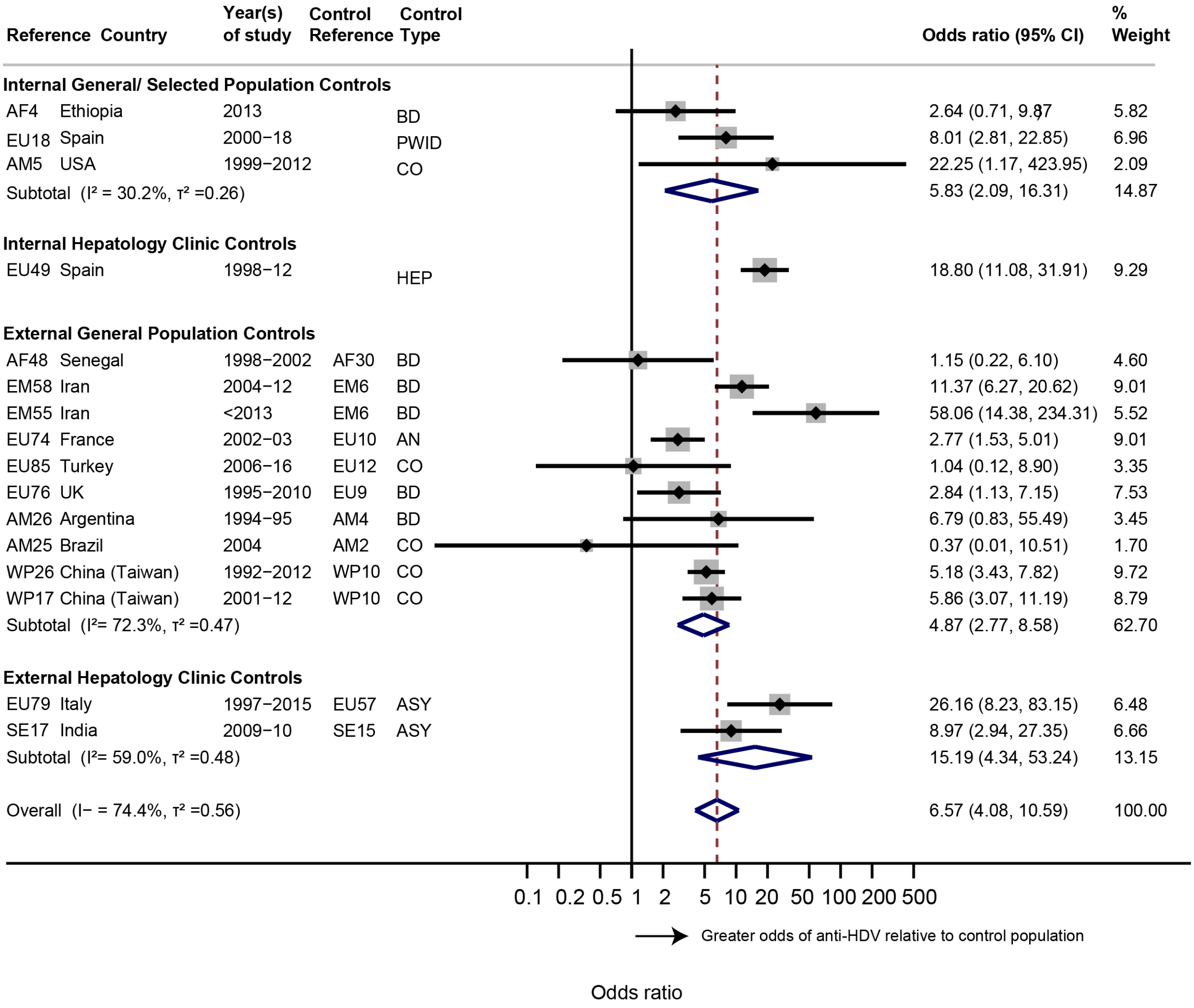

[a]Comparator populations: Blood donors (BD), HIV-positive and HIV-negative people who inject drugs (PWID), Community (CO), Hepatology clinic population (all, analysed by HIV status) (HEP), Antenatal population (AN), Asymptomatic HBsAg-positive people (ASY); [b]Generalised HIV epidemics were defined by an adult HIV prevalence >1%.

Abbreviations: CI, confidence interval; USA, The United States of America.

Supplementary appendix 20. Forest Plot of Anti-HDV Prevalence Among People with HIV Relative to Comparator Populations[a] in Countries with Generalised HIV Epidemics[b]

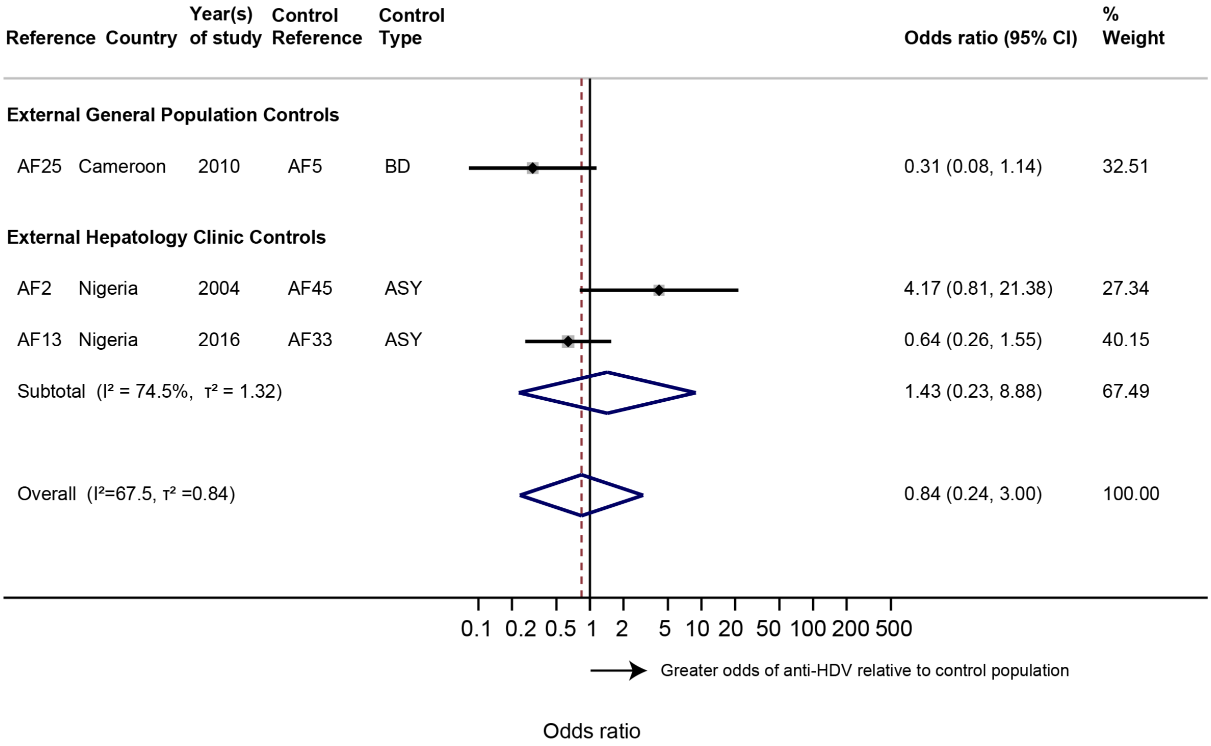

[a]Comparator populations: Blood donors (BD), Asymptomatic HBsAg-positive people (ASY); Generalised HIV epidemics were defined by an adult HIV prevalence >1%.

Abbreviations: CI, confidence interval.

Supplementary appendix 21. Meta-Regression Analysis of Characteristics Associated with HDV RNA Detection Among People with Anti-HDV

Characteristic	HDV RNA prevalence % (95% CI)		Co-efficient[a] (95% CI)		P value
Overall	58.5	(52.4 - 64.5)			
**WHO Region**					
Europe	64.1	(54.3 – 73.3)	Reference		
Africa	41.3	(31.8 – 51.1)	-0.13	(-0.28 – 0.02)	0.09
Americas	64.2	(21.5 – 98.0)	0	(-0.29 – 0.30)	0.98
Eastern Mediterranean	49.4	(30.1 – 68.7)	-0.09	(-0.27 – 0.09)	0.32
South-East Asia	50.1	(31.4 – 70.3)	-0.09	(-0.47 – 0.29)	0.65
Western Pacific	73.3	(57.8 – 86.7)	0.07	(-0.07 – 0.22)	0.33
**Type**					
Hepatology clinic	65.7	(58.3 – 72.7)	Reference		
General population	38.5	(25.9 – 51.8)	-0.20	(-0.33 – -0.07)	0.003
Isolated population	62.7	(26.2 – 93.8)	-0.02	(-0.48 – 0.44)	0.95
Selected population group	63.8	(49.6 – 77.0)	-0.02	(-0.16 – 0.11)	0.73
**HDV RNA detection method**					
qRT-PCR	50.1	(41.6 – 58.6)	Reference		
End point PCR	63.3	(53.8 – 72.4)	0.05	(-0.06 – 0.17)	0.33
Not specified	76.6	(52.4 – 64.5)			
**Anti-HDV prevalence**					
Per 10% increase			0.03	(0.02 – 0.06)	<0.001

[a]The co-efficient refers to the change in HDV RNA prevalence per unit change in the reported characteristics/explanatory variables, where for example a co-efficient of 0.1 refers to an increase of 10% in HDV RNA detection.

Abbreviations: CI, confidence interval; qRT-PCR, quantitative real time reverse transcriptase polymerase chain reaction.

Supplementary appendix 22. List of Included Studies Reporting HDV Genotype Data by WHO Region

African Region (AFR)

AF2. Andernach IE, Leiss LV, Tarnagda ZS, et al. Characterization of hepatitis delta virus in sub-Saharan Africa. *J Clin Microbiol* 2014; 52: 1629-36.

AF4. Belyhun Y, Liebert UG, Maier M. Clade homogeneity and low rate of delta virus despite hyperendemicity of hepatitis B virus in Ethiopia. *Virol J* 2017; 14: 176.

AF5. Butler EK, Rodgers MA, Coller KE, et al. High prevalence of hepatitis delta virus in Cameroon. *Sci Rep* 2018; 8: 11617.

AF9. Ducancelle A, Abgueguen P, Birguel J, et al. High endemicity and low molecular diversity of hepatitis B virus infections in pregnant women in a rural district of North Cameroon. *PloS One* 2013; 8: e80346.

AF11. Groc S, Abbate JL, Le Gal F, et al. High prevalence and diversity of hepatitis B and hepatitis delta virus in Gabon*. J Viral Hepat* 2019; 26: 170-82.

AF16. Komas NP, Ghosh S, Abdou-Chekaraou M, et al. Hepatitis B and hepatitis D virus infections in the Central African Republic, twenty-five years after a fulminant hepatitis outbreak, indicate continuing spread in asymptomatic young adults. *PLoS Negl Trop Dis* 2018; 12: e0006377.

AF18. Makuwa M, Caron M, Souquiere S, Malonga-Mouelet G, Mahe A, Kazanji M. Prevalence and genetic diversity of hepatitis B and delta viruses in pregnant women in Gabon: molecular evidence that hepatitis delta virus clade 8 originates from and is endemic in central Africa*. J Clin Microbiol* 2008; 46: 754-6.

AF19. Makuwa M, Mintsa-Ndong A, Souquiere S, Nkoghe D, Leroy EM, Kazanji M. Prevalence and molecular diversity of hepatitis B virus and hepatitis delta virus in urban and rural populations in northern Gabon in central Africa. *J Clin Microbiol* 2009; 47: 2265-8.

AF20. Mansour W, Bollahi MA, Hamed CT, et al. Virological and epidemiological features of hepatitis delta infection among blood donors in Nouakchott, Mauritania. *J Clin Virol* 2012; 55: 12-6.

AF21. Mansour W, Malick FZ, Sidiya A, et al. Prevalence, risk factors, and molecular epidemiology of hepatitis B and hepatitis delta virus in pregnant women and in patients in Mauritania. *J Med Virol* 2012; 84: 1186-98.

AF24. Pinho-Nascimento CA, Bratschi MW, Hofer R, et al. Transmission of Hepatitis B and D Viruses in an African Rural Community. *mSystems* 2018; 3.

AF27. Stockdale AJ, Chaponda M, Beloukas A, et al. Prevalence of hepatitis D virus infection in sub-Saharan Africa: a systematic review and meta-analysis. Lancet Glob Health 2017; 5: e992-e1003.

AF34. Aberra H, Gordien E, Desalegn H, et al. Hepatitis delta virus infection in a large cohort of chronic hepatitis B patients in Ethiopia. *Liver Int* 2017.

AF38. Foupouapouognigni Y, Noah DN, Sartre MT, Njouom R. High prevalence and predominance of hepatitis delta virus genotype 1 infection in Cameroon. *J Clin Microbiol* 2011; 49: 1162-4.

AF39. Gourari S, Brichler S, Le Gal F, et al. Hepatitis B virus and hepatitis delta virus subtypes circulating in Algeria and seroprevalence of HDV infection. *J Med Virol* 2019; 91: 72-80.

AF40. Lunel-Fabiani F, Mansour W, Amar AO, et al. Impact of hepatitis B and delta virus co-infection on liver disease in Mauritania: a cross sectional study. *J Infect* 2013; 67: 448-57.

AF41. Mahale P, Aka P, Chen X, et al. Hepatitis D virus infection, cirrhosis, and hepatocellular carcinoma in The Gambia. *J Viral Hepat* 2019.

AF46. Opaleye OO, Japhet OM, Adewumi OM, et al. Molecular epidemiology of hepatitis D virus circulating in southwestern Nigeria. *Virol J* 2016; 13: 61.

AF51. Makiala-Mandanda S, Le Gal F, Ngwaka-Matsung N, et al. High Prevalence and diversity of hepatitis viruses in suspected cases of Yellow Fever in the Democratic Republic of Congo. *J Clin Microbiol* 2017; 55: 1299-312.

Eastern Mediterranean Region (EMR)

EM7. Djebbi A, Rebai WK, Bahri O, Hogga N, Sadraoui A, Triki H. [serological markers, viral RNA and genotype of hepatitis delta virus in hbs antigen positive tunisian patients]. *Pathol Biol* 2009; 57: 518-23.

EM13. Ramia S, El-Zaatari M, Sharara AI, Ramlawi F, Farhat B. Current prevalence of hepatitis delta virus (HDV) infection and the range of HDV genotypes in Lebanon. *Epidemiol Infect* 2007; 135: 959-62.

EM16. Saudy N, Sugauchi F, Tanaka Y, et al. Genotypes and phylogenetic characterization of hepatitis B and delta viruses in Egypt. *J Med Virol* 2003; 70: 529-36.

EM32. Jamjoom GA, El-Daly MM, Azhar EI, et al. Prevalence and molecular characterization of hepatitis D virus in Saudi Arabia: A single-center study. *Saudi J Gastroenterol* 2017; 23: 176-82.

EM51. Yacoubi L, Brichler S, Mansour W, et al. Molecular epidemiology of hepatitis B and Delta virus strains that spread in the Mediterranean North East Coast of Tunisia. *J Clin Virol* 2015; 72: 126-32.

EM55. Aghasadeghi MR, Mohraz M, Bahramali G, et al. Frequency and Genotype of Hepatitis D Virus Infection in Patients Infected with HIV and Those Undergoing Hemodialysis. *Hepat Mon* 2013; 13: e7481.

EM63. Aftab M, Amin I, Idrees M, Ali A, Rafique S, Naz S. Molecular epidemiology of hepatitis delta and hepatitis B viruses circulating in two major provinces (East and North-West) of Pakistan. *Infect Genet Evol* 2018; 64: 65-9.

EM64. Behzadian F, Sabahi F, Karimi M, et al. Molecular phylogenetic analysis of Iranian HDV complete genome. *Virus Genes* 2005; 30: 383-93.

EM65. Butt FA, Amin I, Idrees M, Iqbal M. Hepatitis delta virus genotype-1 alone cocirculates with hepatitis B virus genotypes A and D in Pakistan. *Eur J Gastroenterol Hepatol* 2014; 26: 319-24.

EM66. Esmaeili R, Sabouri E, Edalat R, et al. Phylogenetic analysis of twenty-six cases of hepatitis delta virus isolates in Tehran, Iran. *Hepat Mon* 2009; 9: 196-200.

EM67. Karimzadeh H, Usman Z, Frishman D, Roggendorf M. Genetic diversity of hepatitis D virus genotype-1 in Europe allows classification into subtypes. *J Viral Hepat* 2019; 26: 900-10.

EM68. Mohebbi SR, Zali N, Derakhshan F, et al. Molecular epidemiology of hepatitis delta virus (HDV) in Iran: a preliminary report. *J Med Virol* 2008; 80: 2092-9.

EM69. Perveen S, Nasir MI, Shahid SM, Azhar A, Khan OY. Phylogenetic analysis of HDV isolates from HBsAg positive patients in Karachi, Pakistan. *Virol J* 2012; 9: 162.

EM70. Shirvani-Dastgerdi E, Amini-Bavil-Olyaee S, Alavian SM, Trautwein C, Tacke F. Comprehensive analysis of mutations in the hepatitis delta virus genome based on full-length sequencing in a nationwide cohort study and evolutionary pattern during disease progression. Clin Microbiol Infect 2015; 21: 510.e11-510.e23.

European Region (EUR)

EU9. Rosenberg GK, Lattimore S, Brailsford SR, et al. The diversity of chronic hepatitis B virus infections within blood donors in England and North Wales 2005 through 2010. *Transfusion* 2013; 53: 2467-76.

EU11. Servant-Delmas A, Le Gal F, Gallian P, Gordien E, Laperche S. Increasing prevalence of HDV/HBV infection over 15 years in France. *J Clin Virol* 2014; 59: 126-8.

EU22. Bielawski KP, Zietkowski D, Charmuszko U, Sikorska K, Stalke P. Hepatitis delta virus infection in chronically HBV-infected patients from northern Poland. *Arch Virol* 2006; 151: 1207-15.

EU33. El Bouzidi K, Elamin W, Kranzer K, et al. Hepatitis delta virus testing, epidemiology and management: a multicentre cross-sectional study of patients in London. *J Clin Virol* 2015; 66: 33-7.

EU42. Khan A, Kurbanov F, Tanaka Y, et al. Epidemiological and clinical evaluation of hepatitis B, hepatitis C, and delta hepatitis viruses in Tajikistan. *J Med Virol* 2008; 80: 268-76.

EU54. Popescu GA, Otelea D, Gavriliu LC, et al. Epidemiology of hepatitis D in patients infected with hepatitis B virus in Bucharest: A cross-sectional study. *J Med Virol* 2013; 85: 769-74.

EU73. Beguelin C, Moradpour D, Sahli R, et al. Hepatitis delta-associated mortality in HIV/HBV-coinfected patients. J Hepatol 2017; 66: 297-303.

EU81. Rehak V, Krekulova L. Hidden microepidemic of hepatitis D in the population of injecting drug users in Prague, Czech Republic. *Hepatol Int* 2013; 7.
EU86. Altuglu I, Ozacar T, Sertoz RY, Erensoy S. Hepatitis delta virus (HDV) genotypes in patients with chronic hepatitis: molecular epidemiology of HDV in Turkey. *Int J Infect Dis* 2007; 11: 58-62.
EU87. Bozdayi AM, Aslan N, Bozdayi G, et al. Molecular epidemiology of hepatitis B, C and D viruses in Turkish patients. *Arch Virol* 2004; 149: 2115-29.
EU88. Bulut Y, Bahcecioglu IH, Aygun C, Oner PD, Ozercan I, Demirdag K. High genetic diversity of hepatitis delta virus in eastern Turkey. *J Infect Devel Cntrs* 2014; 8: 74-8.
EU89. Çelik I, Karatayli E, Çevik E, et al. Complete genome sequences and phylogenetic analysis of hepatitis delta viruses isolated from nine Turkish patients. *Arch Virol* 2011; 156: 2215-20.
EU90. Cotrina M, Buti M, Jardi R, et al. Hepatitis delta genotypes in chronic delta infection in the northeast of Spain (Catalonia). *J Hepatol* 1998; 28: 971-7.
EU91. Flodgren E, Bengtsson S, Knutsson M, et al. Recent high incidence of fulminant hepatitis in Samara, Russia: Molecular analysis of prevailing hepatitis B and D virus strains. *J Clin Microbiol* 2000; 38: 3311-6.
EU92. Gori A, Gheorgita S, Spinu C, et al. Hepatitis B, C and D virus genotypes detected in HBsAg- or anti-HCV-positive people from the Republic of Moldova. *Arch Virol* 2018; 163: 431-8.
EU93. Hofmann J, Frenzel K, Minh BQ, et al. Quantitative detection and typing of hepatitis D virus in human serum by real-time polymerase chain reaction and melting curve analysis. *Diagn Microbiol Infect Dis* 2010; 67: 172-9.
EU94. Ivaniushina V, Radjef N, Alexeeva M, et al. Hepatitis delta virus genotypes I and II cocirculate in an endemic area of Yakutia, Russia. *J Gen Virol* 2001; 82: 2709-18.
EU95. Karimzadeh H, Usman Z, Frishman D, Roggendorf M. Genetic diversity of hepatitis D virus genotype-1 in Europe allows classification into subtypes. *J Viral Hepat* 2019; 26: 900-10.
EU96. Le Gal F, Badur S, Hawajri NA, et al. Current hepatitis delta virus type 1 (HDV1) infections in central and eastern Turkey indicate a wide genetic diversity that is probably linked to different HDV1 origins. *Arch Virol* 2012; 157: 647-59.
EU97. Le Gal F, Brichler S, Drugan T, et al. Genetic diversity and worldwide distribution of the deltavirus genus: A study of 2,152 clinical strains. *Hepatology* 2017; 66: 1826-41.
EU98. Niro GA, Smedile A, Andriulli A, Rizzetto M, Gerin JL, Casey JL. The predominance of hepatitis delta virus genotype I among chronically infected Italian patients. *Hepatology* 1997; 25: 728-34.
EU99. Shakil AO, Hadziyannis S, Hoofnagle JH, Di Bisceglie AM, Gerin JL, Casey JL. Geographic distribution and genetic variability of hepatitis delta virus genotype I. *Virology* 1997; 234: 160-7.
EU100. Shirazi R, Ram D, Rakovsky A, et al. Characterization of hepatitis B and delta coinfection in Israel. *BMC Infect Dis* 2018; 18: 97.
EU101. Zhang YY, Hansson BG. Introduction of a new hepatitis agent in retrospect: genetic studies of Swedish hepatitis D virus strains. *J Clin Microbiol* 1996; 34: 2713-7.

Region of the Americas (AMR)

AM3. Crispim MA, Fraiji NA, Campello SC, Schriefer NA, Stefani MM, Kiesslich D. Molecular epidemiology of hepatitis B and hepatitis delta viruses circulating in the Western Amazon region, north Brazil. *BMC Infect Dis* 2014; 14: 94.
AM4. Delfino CM, Gentile EA, Castillo AI, et al. Hepatitis B virus and hepatitis D virus in blood donors from Argentina: Circulation of HBsAg and reverse transcriptase mutants. *Arch Virol* 2014; 159: 1109-17.
AM10. di Filippo Villa D, Cortes-Mancera F, Payares E, et al. Hepatitis D virus and hepatitis B virus infection in Amerindian communities of the Amazonas state, Colombia. *Virol J* 2015; 12: 172.
AM16. Barros LM, Gomes-Gouvea MS, Pinho JR, et al. Hepatitis delta virus genotype 8 infection in northeast Brazil: inheritance from African slaves? *Virus Res* 2011; 160: 333-9.
AM18. Santos MD, Gomes-Gouvea MS, Nunes JD, et al. The hepatitis delta genotype 8 in northeast Brazil: The North Atlantic slave trade as the potential route for infection. *Virus Res* 2016; 224: 6-11.

AM31. Mendes-Correa MC, Gomes-Gouvea MS, Alvarado-Mora MV, et al. Hepatitis delta in HIV/HBV co-infected patients in Brazil: is it important? *Int J Infect Dis* 2011; 15: e828-32.
AM34. Alvarado-Mora MV, Romano CM, Gomes-Gouvea MS, Gutierrez MF, Carrilho FJ, Pinho JR. Dynamics of hepatitis D (delta) virus genotype 3 in the Amazon region of South America. *Infect Genet Evol* 2011; 11: 1462-8.
AM35. Botelho-Souza LF, Souza Vieira D, de Oliveira Dos Santos A, Cunha Pereira AV, Villalobos-Salcedo JM. Characterization of the genotypic profile of hepatitis delta virus: Isolation of HDV genotype-1 in the western Amazon region of Brazil. *Intervirology* 2015; 58: 166-71.
AM36. Casey JL, Brown TL, Colan EJ, Wignall FS, Gerin JL. A genotype of hepatitis D virus that occurs in northern South America. *Proc Natl Acad Sci U S A* 1993; 90: 9016-20.
AM37. Cicero MF, Pena NM, Santana LC, et al. Is hepatitis delta infections important in Brazil? *BMC Infect Dis* 2016; 16: 525.
AM38. Gomes-Gouvea MS, Pereira Soares Mdo C, Guedes de Carvalho Mello IM, et al. Hepatitis D and B virus genotypes in chronically infected patients from the eastern Amazon basin. *Acta Trop* 2008; 106: 149-55.
AM39. Gomes-Gouvea MS, Soares MC, Bensabath G, et al. Hepatitis B virus and hepatitis delta virus genotypes in outbreaks of fulminant hepatitis (Labrea black fever) in the western Brazilian Amazon region. *J Gen Virol* 2009; 90: 2638-43.
AM40. Kay A, Melo da Silva E, Pedreira H, et al. HBV/HDV co-infection in the western Brazilian Amazonia: An intriguing mutation among HDV genotype 3 carriers. *J Viral Hepat* 2014; 21: 921-4.
AM41. Kefalakes H, Koh C, Sidney J, et al. Hepatitis D virus-specific CD8(+) T cells have a memory-like phenotype associated with viral immune escape in patients with chronic hepatitis D virus infection. *Gastroenterology* 2019; 156: 1805-1819.
AM42. Nakano T, Shapiro CN, Hadler SC, et al. Characterization of hepatitis D virus genotype III among Yucpa Indians in Venezuela. *J Gen Virol* 2001; 82: 2183-9.
AM43. Parana R, Kay A, Molinet F, et al. HDV genotypes in the western Brazilian Amazon region: A preliminary report. *Am J Trop Med Hyg* 2006; 75: 475-9.
AM44. Poisson V, Ménard DB, Frost E, Perreault JP. A Canadian isolate of hepatitis D (delta) virus. *Canadian J Gastroenterol* 2000; 14: 36B-40B.
AM45. Quintero A, Uzcátegui N, Loureiro CL, et al. Hepatitis delta virus genotypes I and III circulate associated with hepatitis B virus genotype F in Venezuela. *J Med Virol* 2001; 64: 356-9.
AM46. Shakil AO, Hadziyannis S, Hoofnagle JH, Di Bisceglie AM, Gerin JL, Casey JL. Geographic distribution and genetic variability of hepatitis delta virus genotype I. *Virology* 1997; 234: 160-7.

South-East Asia Region (SEAR)

SE12. Kim HS, Kim SJ, Park HW, et al. Prevalence and clinical significance of hepatitis D virus co-infection in patients with chronic hepatitis B in Korea. *J Med Virol* 2011; 83: 1172-7.
SE4. Theamboonlers A, Hansurabhanon T, Verachai V, Chongsrisawat V, Poovorawan Y. Hepatitis D virus infection in Thailand: HDV genotyping by RT-PCR, RFLP and direct sequencing. *Infection* 2002; 30: 140-4.

Western Pacific Region (WPR)

WP2. Chang CJ, Chiang JC, Lu SN, Wang JH. Hepatitis delta virus and GBV-C infection in two neighboring hepatitis B virus and hepatitis C virus-endemic villages in Taiwan. *Chang Gung Med J* 2010; 33: 137-44.
WP4. Chen F, Zhang J, Guo F, et al. Hepatitis B, C, and D virus infection showing distinct patterns between injection drug users and the general population. *J Gastroenterol Hepatol* 2017; 32: 515-20.
WP13. Lu SN, Chen TM, Lee CM, Wang JH, Tung HD, Wu JC. Molecular epidemiological and clinical aspects of hepatitis D virus in a unique triple hepatitis viruses (B, C, D) endemic community in Taiwan. *J Med Virol* 2003; 70: 74-80.

WP14. Nguyen VT, McLaws ML, Dore GJ. Highly endemic hepatitis B infection in rural Vietnam. *J Gastroenterol Hepatol* 2007; 22: 2093-100.
WP15. Tsatsralt-Od B, Takahashi M, Nishizawa T, Inoue J, Ulaankhuu D, Okamoto H. High prevalence of hepatitis B, C and delta virus infections among blood donors in Mongolia. *Arch Virol* 2005; 150: 2513-28.
WP25. Hall N, Thuy LN, Diem Tdo T, et al. High prevalence of hepatitis delta virus among persons who inject drugs, Vietnam. *Emerg Infect Dis* 2015; 21: 540-3.
WP26. Hung CC, Wu SM, Lin PH, et al. Increasing incidence of recent hepatitis D virus infection in HIV-infected patients in an area hyperendemic for hepatitis B virus infection. *Clin Infect Dis* 2014; 58: 1625-33.
WP31. Binh MT, Hoan NX, Van Tong H, et al. HDV infection rates in northern Vietnam. *Sci Rep* 2018; 8: 8047.
WP32. Kurbanov F, Tanaka Y, Elkady A, Oyunsuren T, Mizokami M. Tracing hepatitis C and Delta viruses to estimate their contribution in HCC rates in Mongolia. *J Viral Hepat* 2007; 14: 667-74.
WP33. Liao B, Chen W, Ping Q, et al. Complete Genome Sequence of a Hepatitis D Virus Genotype 1 Strain Isolated in Guangdong, China. *Genome Announc* 2015; 3.
WP34. Ma J, J W, Jia X, Huang W, Liang Z, Wang Y. Cloning and sequence analysis of full-length cDNA of hepatitis D virus in China. *J Microbes Infect* 2015; 10: 345-50.
WP35. Ma SP, Makino Y, Sakugawa H, Kinjo F, Saito A, Tadano M. The complete genomic sequence of hepatitis delta virus genotype llb prevalent in Okinawa, Japan. *J Gen Virol* 2003; 84: 461-4.
WP36. Sakugawa H, Nakasone H, Nakayoshi T, et al. Hepatitis delta virus genotype IIb predominates in an endemic area, Okinawa, Japan. *J Med Virol* 1999; 58: 366-72.
WP37. Su CW, Huang YH, Huo TI, et al. Genotypes and viremia of hepatitis B and D viruses are associated with outcomes of chronic hepatitis D patients. *Gastroenterology* 2006; 130: 1625-35.
WP38. Sy BT, Nguyen HM, Toan NL, et al. Identification of a natural intergenotypic recombinant hepatitis delta virus genotype 1 and 2 in Vietnamese HBsAg-positive patients. *J Viral Hepat* 2015; 22: 55-63.
WP39. Takahashi M, Nishizawa T, Gotanda Y, et al. High prevalence of antibodies to hepatitis A and E viruses and viremia of hepatitis B, C, and D viruses among apparently healthy populations in Mongolia. *Clin Diagn Lab Immunol* 2004; 11: 392-8.
WP40. Tsatsralt-Od B, Takahashi M, Endo K, et al. Prevalence of hepatitis B, C, and delta virus infections among children in Mongolia: progress in childhood immunization. *J Med Virol* 2007; 79: 1064-74.
WP41. Tsatsralt-Od B, Takahashi M, Endo K, et al. Infection with hepatitis A, B, C, and delta viruses among patients with acute hepatitis in Mongolia. *J Med Virol* 2006; 78: 542-50.
WP42. Watanabe H, Nagayama K, Enomoto N, et al. Chronic hepatitis delta virus infection with genotype IIb variant is correlated with progressive liver disease. *J Gen Virol* 2003; 84: 3275-89.
WP43. Wu JC, Chen TZ, Huo TI, Lee S, Choo KB, Chen CM. Genotyping of hepatitis D virus by restriction-fragment length polymorphism and relation to outcome of hepatitis D. *Lancet* 1995; 346: 939-41.

Supplementary appendix 23. HDV Genotype Geographic Distribution Data

Ref	Year	Country	Region	N	Genotype (n)
EU101	1996	Sweden	Malmö	7	G1 (7)
EH9	2013	United Kingdom	National	2	G1 (2)
EU33	2015	United Kingdom	London	25	G1 (19) G5 (6)
EU93	2010	Germany	Berlin, Essen	42	G1 (42)
EU95	2019	Germany	Munich, Hannover	72	G1 (72)
EU73	2017	Switzerland	National	70	G1 (66) G5 (4)
EU11	2014	France	National	14	G1 (12) G6 (1) G7 (1)
EU97	2017	France	National	2152	G1 (1734) G2 (7) G3 (7) G5 (291) G6 (29) G7 (60) G8 (24)
EU90	1998	Spain	Catalonia	37	G1 (37)
EU95	2019	Spain	Madrid	12	G1 (12)
EU98	1997	Italy	Foggia	46	G1 (46)
EU95	2019	Italy	Turin	18	G1 (18)
EH81	2013	Czechia	Remedis	7	G1 (7)
EU22	2006	Poland	Gdansk	4	G1 (4)
EU91	2000	Russian Federation	Samara	4	G1 (4)
EU94	2001	Russian Federation	Yakutia	29	G1 (14) G2 (15)
EU92	2018	Republic of Moldova	Kishinev	2	G1 (2)
EU54	2013	Romania	Bucharest	38	G1 (38)
EU99	1997	Greece	Achangelos	12	G1 (12)
EU99	1997	Greece	Mainland	15	G1 (15)
EU87	2004	Turkey	Ankara	13	G1 (13)
EU86	2007	Turkey	Izmir	26	G1 (26)
EU89	2011	Turkey	Ankara	9	G1 (9)
EU96	2012	Turkey	Central Eastern Turkey	34	G1 (34)
EU88	2014	Turkey	Elazig	40	G1 (40)
EM13	2007	Lebanon	National	1	G1 (1)
EU100	2018	Israel	National	55	G1 (55)
EM64	2004	Iran	Tehran	1	G1 (1)
EM68	2008	Iran	Tehran	22	G1 (22)
EM66	2009	Iran	Tehran	26	G1 (26)
EM55	2013	Iran	Tehran	3	G1 (3)
EM70	2015	Iran	Tehran	38	G1 (38)
EM67	2019	Iran	Tehran	14	G1 (14)
EM32	2017	Saudi Arabia	Jeddah	1	G1 (1)
EU42	2008	Tajikistan	Dushanbe	10	G1 (10)
EM69	2012	Pakistan	Karachi	22	G1 (22)
EM65	2014	Pakistan	Lahore	21	G1 (21)
EM63	2018	Pakistan	Lahore	17	G1 (17)
WP39	2004	Mongolia	Ulaanbaatur	20	G1 (20)
WP41	2006	Mongolia	Ulaanbaatur	32	G1 (32)
WP15	2005	Mongolia	Ulaanbaatur	117	G1 (117)
WP40	2007	Mongolia	Ulaanbaatur	13	G1 (13)
WP32	2007	Mongolia	Ulaanbaatur	24	G1 (24)

WP33	2015	China	Guandong	1	G1 (1)
WP34	2015	China	Beijing	3	G1 (3)
WP4	2017	China	Hunan Province	48	G2 (48)
WP43	1995	China (Taiwan)	Taipei	14	G1 (8) G2(6)
WP13	2003	China (Taiwan)	Tzukuan Township	4	G2 (4)
WP37	2006	China (Taiwan)	Tapei	61	G1 (15) G2 (41) G4 (5)
WP26	2014	China (Taiwan)	Tapei	28	G2 (9) G4 (19)
WP2	2011	China (Taiwan)	Yunlin County	100	G1 (5) G2 (42) G4 (53)
SE4	2002	Thailand	Prathumtani and Songkla Province	8	G1 (8)
WP14	2007	Vietnam	Thai Binh province	25	G1 (5) G2 (20)
WP38	2014	Vietnam	Hanoi	21	G1 (19) G2 (2)
WP25	2015	Vietnam	Ha Noi	17	G1 (12) G2 (5)
WP31	2018	Vietnam	Hanoi	57	G1 (52) G2 (5)
SE12	2011	Republic of Korea	Kildong, Kangdong-gu, Seoul	2	G1 (2)
WP36	1999	Japan	Mikayo Island	6	G4 (6)
WP42	2003	Japan	Mikayo Island	33	G1 (2) G2 (1) G4 (30)
WP35	2003	Japan	Okinawa	1	G2 (1)
EM16	2003	Egypt	Mansoura	7	G1 (9)
EM70	2009	Tunisia	Tunis	1	G1 (1)
EM51	2015	Tunisia	Bizerte, Tunis, Nabeul, Sousse, Monastir	11	G1 (11)
AF39	2018	Algeria	Algiers	1	G1 (1)
AF20	2012	Mauritania	Nouakchott	28	G1 (25) G5 (3)
AF21	2012	Mauritania	Nouakchott	31	G1(28) G5 (3)
AF40	2013	Mauritania	Nouakchott	47	G1 (40) G5 (7)
AF41	2019	Gambia	Banjul, Fajara, Bansang	17	G1 (5) G5 (12)
AF27	2017	Ghana	Kumasi	2	G1 (1) G5 (1)
AF2	2014	Nigeria	Ibadan	1	G1 (1)
AF2	2014	Nigeria	Lagos	2	G1 (1) G6 (1)
AF46	2016	Nigeria	Osogbo, Ogbomoso, Ile-Ife, Ibadan	14	G1 (14)
AF38	2011	Cameroon	Yaoundé	25	G1 (22) G5 (1) G6 (1) G7 (1)
AF9	2013	Cameroon	Tokombéré	9	G1 (9)
AF51	2018	Cameroon	Yaoundé, Douala	211	G1 (138) G7 (61) G6 (11) G8 (1)
AF24	2018	Cameroon	Bankim District	3	G1 (3)
AF18	2008	Gabon	Libreville	3	G1 (1) G8 (2)
AF19	2009	Gabon	Woleu-Ntem (rural)	17	G1 (10) G7 (2) G8 (5)
AF11	2019	Gabon	Estuaire	40	G1 (21) G7 (7) G8 (12)
AF2	2014	CAR	Bangui	5	G1 (5)
AF16	2018	CAR	Bangui	18	G1 (18)
AF51	2017	DRC	Orientale Equateur Provinces	12	G1 (12)
AF34	2017	Ethiopia	Addis Ababa	12	G1 (12)
AF4	2017	Ethiopia	Gondar	6	G1 (6)
AM44	2000	Canada	Quebec	1	G1 (1)
AM46	1997	USA	Maryland	22	G1 (22)
AM41	2019	USA	Bethesda, MD	28	G1 (28)
AM42	2001	Venezuela	NorthWest	3	G3 (3)

AM45	2001	Venezuela	Sierra de Perija	6	G1 (6)
AM45	2001	Venezuela	Amazonas	4	G3 (4)
AM34	2007	Colombia	Leiticia	5	G3 (5)
AF10	2015	Colombia	Amazonas State	8	G1 (1) G3 (7)
AM36	1993	Peru	Yavari River	2	G3 (2)
AM43	2006	Brazil	Acre and Rodondo	40	G1 (22) G3 (18)
AM39	2008	Brazil	East Amazon	14	G3 (14)
AM38	2009	Brazil	Western Amazon	14	G3 (14)
AF16	2011	Brazil	Maranhão	3	G3 (1) G8 (2)
AF31	2011	Brazil	Sao Paulo	1	G1 (1)
AM3	2014	Brazil	Manaus City	38	G3 (38)
AM3	2014	Brazil	North Brazilian Amazonas	12	G3 (12)
AM3	2014	Brazil	Eirenepe City	20	G3 (20)
AM40	2014	Brazil	Porto Velho, Rio Branco, Cruzeiro do Sul	90	G3 (90)
AM35	2015	Brazil	Rodonia	52	G1 (4) G3 (48)
AF18	2016	Brazil	Maranhao Province	4	G8 (4)
AM37	2016	Brazil	Amazon	46	G3 (46)
AF4	2014	Argentina	Buenos Aires (Central Region)	1	G1 (1)

Abbreviations: DRC, Democratic Republic of the Congo; CAR, Central African Republic; USA, The United States of America.

Supplementary appendix 24. Forest Plot of Anti-HDV Prevalence Among People with Cirrhosis Relative to Comparator Populations[a]

Reference	Country	Year(s) of study	Control Reference	Control Type	Odds ratio (95% CI)	% Weight
**Internal General Population Controls**						
EM13	Lebanon	2002–04		BD	1.45 (0.06, 36.65)	1.31
EM19	Pakistan	1995		BD	6.75 (0.83, 54.66)	2.35
WP20	Mongolia	2004		BD	2.18 (0.58, 8.27)	3.57
Subtotal (I^2 = 0.0%, τ^2 = 0.0)					2.79 (0.97, 8.07)	7.23
**External General Population Controls**						
AF42	Senegal	1995–96	AF30	BD	17.00 (4.77, 60.56)	3.69
AF41	Gambia	1997–2001	AF17	CO	7.49 (2.78, 20.18)	4.24
EM21	Pakistan	2007–11	EM19	BD	4.62 (0.97, 21.90)	3.16
EU6	Belarus	1983–90	EU6	BD	26.36 (9.51, 73.06)	4.18
EU24	France	2006–12	EU11	BD	5.36 (3.50, 8.21)	5.23
Subtotal (I^2= 60.7%, τ^2 = 0.34)					9.30 (4.68, 18.46)	20.49
**Internal Hepatology Population Controls**						
AF43	Nigeria	2006		ASY	2.33 (0.22, 24.92)	2.02
EM16	Egypt	<2003		ASY	2.10 (0.45, 9.84)	3.18
EM33	Iran	2008–11		ASY	25.69 (11.24, 58.72)	4.56
EM24	Iran	2004–05		ASY	3.46 (1.47, 8.14)	4.50
EM30	Iran	2011–12		ASY	190.00 (38.68, 933.20)	3.10
EM26	Libya	2010–12		ASY	48.48 (2.48, 946.56)	1.48
EM46	Pakistan	2007–08		ASY	7.76 (3.97, 15.18)	4.85
EM37	Tunisia	2011–12		ASY	3.01 (0.35, 25.63)	2.29
EU30	Italy	2012–15		ASY	9.86 (4.70, 20.72)	4.72
EU23	Italy	2007–08		ASY	5.23 (1.20, 22.76)	3.31
EU57	Italy	2001		ASY	18.60 (5.69, 60.84)	3.85
EU21	Turkey	2001–2006		ASY	4.81 (3.34, 6.93)	5.30
EU66	Turkey	<2005		ASY	15.52 (6.40, 37.64)	4.44
EU31	Turkey	1994–96		ASY	0.45 (0.14, 1.45)	3.86
SE15	India	2003–05		ASY	0.48 (0.06, 3.76)	2.40
SE12	Republic of Korea	2003		ASY	1.58 (0.06, 41.34)	1.29
SE11	Republic of Korea	2008–10		ASY	0.23 (0.01, 4.53)	1.49
WP23	China	2011		ASY	7.26 (1.56, 33.76)	3.20
WP18	Japan	1990–95		ASY	5.64 (1.73, 18.40)	3.86
WP17	China (Taiwan)	2001–12		ASY	2.42 (1.18, 4.96)	4.76
Subtotal (I^2 = 77.4, τ^2 = 0.89)					5.57 (3.30, 9.38)	68.46
**External Hepatology Population Controls**						
EM44	Iran	<2012	EM33	ASY	114.00 (34.27, 379.24)	3.82
Overall (I^2 = 77.2, τ^2 = 0.85)					6.68 (4.37, 10.20)	100.00

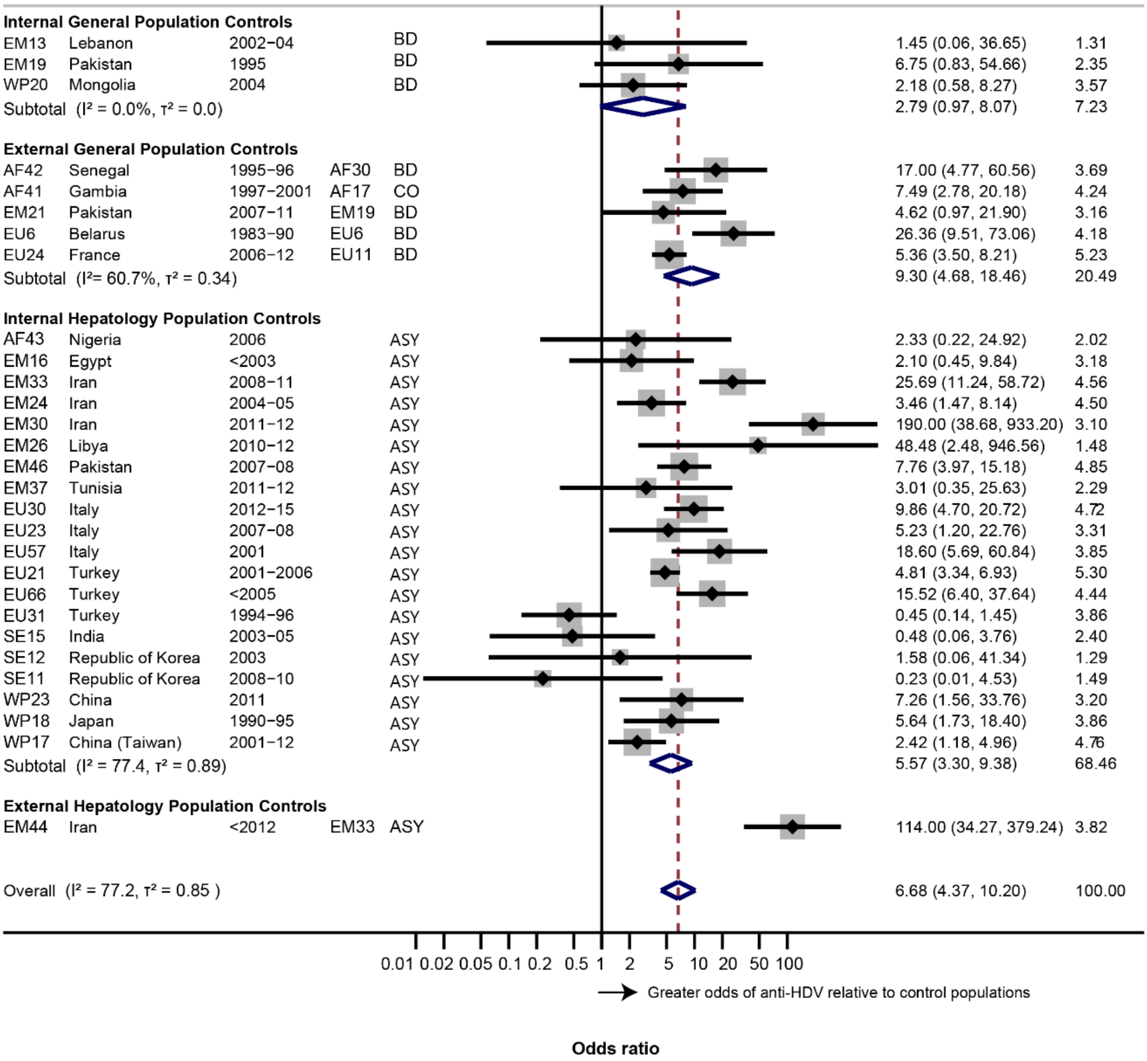

[a]Comparator populations: Blood donors (BD), Community (CO), Asymptomatic HBsAg positive people (ASY).

Abbreviations: CI, confidence interval

Supplementary appendix 25. Forest plot of Anti-HDV Prevalence Among People with Hepatocellular Carcinoma Relative to Comparator Populations[a]

Reference	Country	Year(s) of study	Control Reference	Control Type	Odds ratio (95% CI)	% Weight
**Internal General Population Controls**						
AF35	Cameroon	2013–14		CO	4.40 (2.56, 7.56)	13.12
EM13	Lebanon	2002–04		BD	2.29 (0.09, 58.75)	1.48
WP20	Mongolia	2004		BD	22.18 (2.62, 187.64)	3.07
Subtotal (I^2 = 15.8%, τ^2= 0.17)					5.30 (2.25, 12.50)	17.67
**External General Population Controls**						
AF42	Senegal	1995–96	AF30	BD	8.16 (2.32, 28.74)	6.59
AF41	Gambia	1997–2001	AF17	CO	5.26 (2.24, 12.35)	9.84
EU65	Rep. of Moldova	2014–17	EU4	AN	4.15 (2.00, 8.62)	11.08
EU68	Turkey	2000–04	EU12	CO	22.04 (9.23, 52.62)	9.67
WP19	Mongolia	2005	WP5	CO	2.94 (1.36, 6.38)	10.63
Subtotal (I^2 = 70.1%, τ^2= 0.45)					6.29 (3.08, 12.84)	47.81
**Internal Hepatology Asymptomatic Controls**						
AF43	Nigeria	<2018		ASY	3.05 (1.16, 7.96)	8.84
AF45	Nigeria	2009–10		ASY	1.79 (0.29, 10.99)	3.98
AF33	Nigeria	2006		ASY	2.73 (0.31, 23.82)	3.00
EM52	Egypt	<2011		ASY	2.60 (0.11, 62.57)	1.54
EU57	Italy	2001		ASY	0.74 (0.04, 14.45)	1.73
EU30	Italy	2007–08		ASY	7.67 (0.69, 85.04)	2.51
SE15	India	2003–05		ASY	0.69 (0.04, 12.30)	1.84
SE9	India	1992–98		ASY	9.71 (0.42, 225.84)	1.57
SE11	Rep. of Korea	2008–10		ASY	0.52 (0.03, 10.17)	1.73
SE12	Rep. of Korea	2003		ASY	1.51 (0.08, 28.28)	1.78
WP18	Japan	1990–95		ASY	59.23 (6.74, 520.17)	2.98
Subtotal (I^2= 17.1%, τ^2 = 0.26)					3.13 (1.50, 6.51)	31.52
**External Hepatology Asymptomatic Controls**						
EU31	Turkey	1994–96	EU26	ASY	3.15 (0.36, 27.49)	3.00
Overall (I^2=38.4%, τ^2 = 0.26)					4.80 (3.18, 7.26	100.00

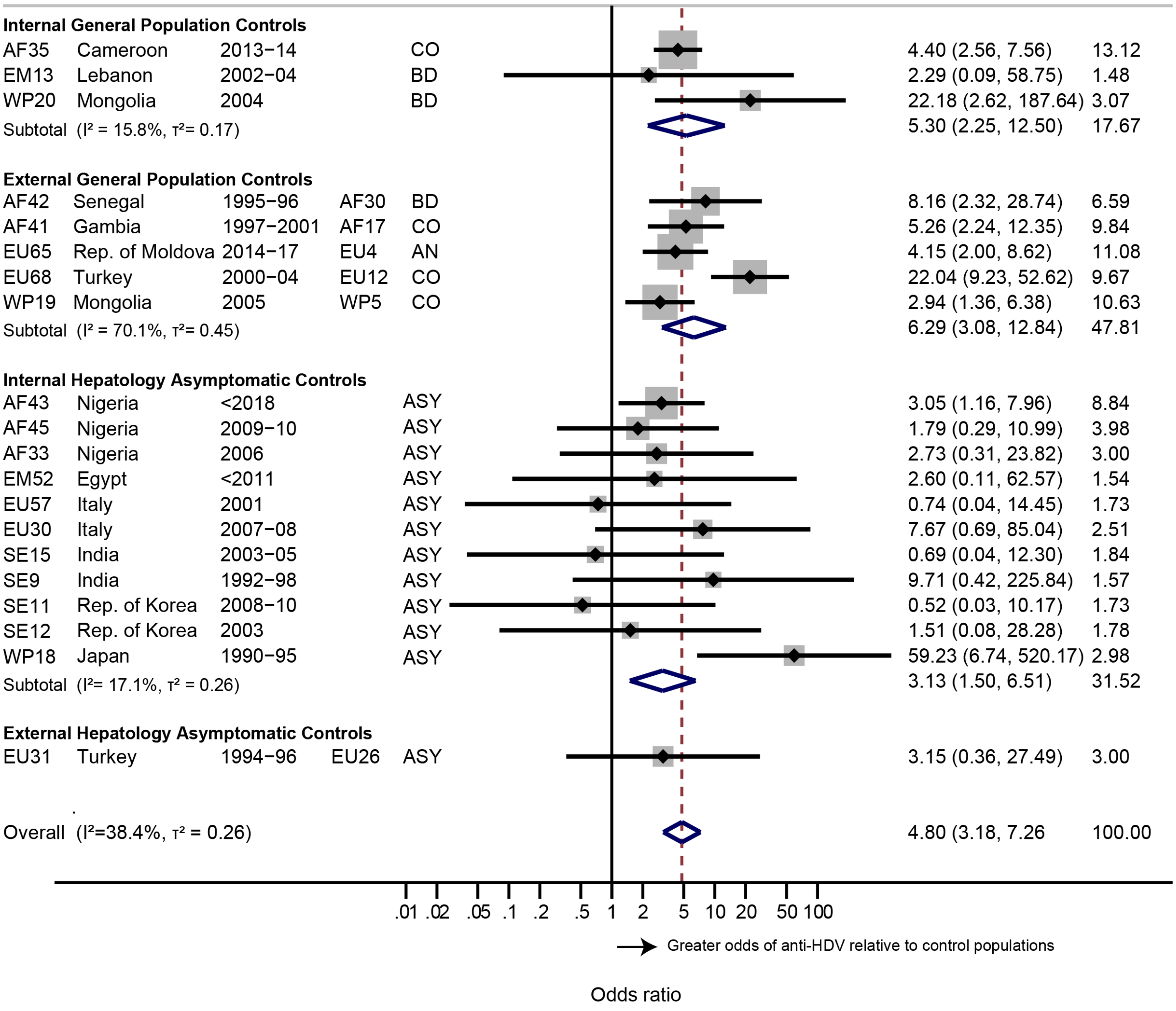

[a]Comparator populations: Community (CO), Blood donors (BD), Antenatal (AN), Asymptomatic HBsAg positive people (ASY).

Abbreviations: CI, Confidence interval; Rep, Republic.

Supplementary appendix 26. Population Attributable Fraction of HDV to the Detection of Cirrhosis Among HBsAg-Positive People

Reference	Country	HDV prev. (cirrhosis cases)	Cirrhosis OR	PAF	95% CI	SE	Weight (%)
AF43	Nigeria	0.14	2.33	0.08	[−0.13; 0.29]	0.11	2.7
AF42	Senegal	0.33	17.00	0.31	[0.11; 0.52]	0.11	2.6
AF41	Gambia	0.13	7.49	0.12	[0.03; 0.20]	0.04	4.4
EM16	Egypt	0.58	2.10	0.31	[−0.33; 0.94]	0.32	0.5
EM30	Iran	0.14	3.46	0.10	[0.04; 0.16]	0.03	4.7
EM44	Iran	0.50	114.00	0.50	[0.25; 0.74]	0.13	2.2
EM33	Iran	0.62	190.00	0.62	[0.29; 0.95]	0.17	1.5
EM24	Iran	0.66	25.69	0.63	[0.48; 0.78]	0.08	3.4
EM13	Lebanon	0.02	1.45	0.01	[−0.05; 0.06]	0.03	4.7
EM26	Libya	0.22	48.48	0.22	[0.04; 0.40]	0.09	3.0
EM19	Pakistan	0.43	6.75	0.37	[−0.05; 0.78]	0.21	1.1
EM46	Pakistan	0.82	7.76	0.71	[0.57; 0.86]	0.07	3.6
EM21	Pakistan	0.34	4.62	0.27	[0.09; 0.44]	0.09	3.1
EM37	Tunisia	0.12	3.01	0.08	[−0.12; 0.29]	0.11	2.7
EU6	Belarus	0.48	26.36	0.46	[0.24; 0.68]	0.11	2.5
EU24	France	0.10	5.36	0.08	[0.05; 0.11]	0.02	4.9
EU30	Italy	0.10	5.23	0.08	[−0.01; 0.17]	0.05	4.3
EU57	Italy	0.17	18.60	0.16	[0.11; 0.21]	0.02	4.8
EU23	Italy	0.17	9.86	0.16	[0.12; 0.20]	0.02	4.9
EU66	Turkey	0.45	15.52	0.42	[0.30; 0.54]	0.06	3.9
EU31	Turkey	0.03	0.45	−0.03	[−0.07; 0.00]	0.02	4.9
EU21	Turkey	0.23	4.81	0.19	[0.14; 0.23]	0.02	4.8
SE15	India	0.03	0.48	−0.03	[−0.09; 0.03]	0.03	4.7
SE12	Rep. of Korea	0.00	0.23	−0.01	[−0.03; 0.01]	0.01	5.0
SE11	Rep. of Korea	0.05	1.58	0.02	[−0.11; 0.15]	0.06	3.8
WP23	China	0.10	7.26	0.08	[−0.01; 0.17]	0.05	4.3
WP18	Japan	0.40	5.64	0.33	[0.05; 0.61]	0.14	1.9
WP20	Mongolia	0.86	2.18	0.46	[−0.35; 1.28]	0.42	0.3
WP17	China (Taiwan)	0.07	2.42	0.04	[0.01; 0.07]	0.02	4.9
**Random effects model**				**0.18**	**[0.10; 0.26]**		**100.0**

Heterogeneity: $I^2 = 93$ $\tau^2 = 0.0126$, $p < 0.01$

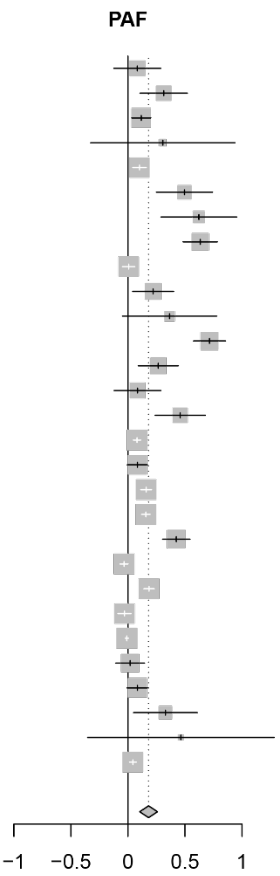

Abbreviations: HBsAg, Hepatitis B surface antigen, Prev., prevalence; OR, odds ratio; PAF, population attributable fraction; CI, confidence interval (normal approximation using standard error calculated via parametric bootstrap); SE standard error (calculated via parametric bootstrap); Rep. Republic.

Supplementary appendix 27. Population Attributable Fraction of HDV to the Detection of Hepatocellular Carcinoma Among HBsAg-Positive People

Reference	Country	HDV prev. (HCC cases)	HCC OR	PAF	95% CI	SE	Weight (%)
AF35	Cameroon	0.41	4.40	0.32	[0.17; 0.47]	0.08	5.1
AF33	Nigeria	0.03	1.79	0.01	[−0.03; 0.06]	0.02	6.4
AF45	Nigeria	0.27	3.05	0.18	[0.00; 0.36]	0.09	4.6
AF43	Nigeria	0.16	2.73	0.10	[−0.06; 0.27]	0.08	4.8
AF42	Senegal	0.19	8.16	0.17	[0.03; 0.31]	0.07	5.2
AF41	Gambia	0.10	5.26	0.08	[0.03; 0.13]	0.02	6.4
EM52	Egypt	0.17	2.60	0.10	[−0.20; 0.40]	0.15	3.0
EM13	Lebanon	0.03	2.29	0.02	[−0.06; 0.10]	0.04	6.0
EU57	Italy	0.01	0.74	−0.00	[−0.03; 0.02]	0.01	6.5
EU30	Italy	0.14	7.67	0.12	[−0.11; 0.35]	0.12	3.9
EU65	Rep. of Moldova	0.48	4.15	0.37	[0.19; 0.54]	0.09	4.7
EU68	Turkey	0.38	22.04	0.37	[0.29; 0.44]	0.04	6.1
EU31	Turkey	0.17	3.15	0.11	[−0.16; 0.39]	0.14	3.4
SE15	India	0.04	0.69	−0.02	[−0.12; 0.09]	0.05	5.7
SE9	India	0.23	9.71	0.20	[−0.05; 0.46]	0.13	3.5
SE11	Rep. of Korea	0.05	1.51	0.02	[−0.08; 0.11]	0.05	5.9
SE12	Rep. of Korea	0.01	0.52	−0.01	[−0.03; 0.01]	0.01	6.5
WP18	Japan	0.88	59.23	0.86	[0.64; 1.08]	0.11	4.0
WP20	Mongolia	0.98	22.18	0.94	[0.79; 1.09]	0.08	5.1
WP19	Mongolia	0.82	2.94	0.54	[0.26; 0.83]	0.14	3.2
**Random effects model**				**0.20**	**[0.08; 0.33]**		**100.0**

Heterogeneity: $I^2 = 94$, $\tau^2 = 0.0202$, $p < 0.01$

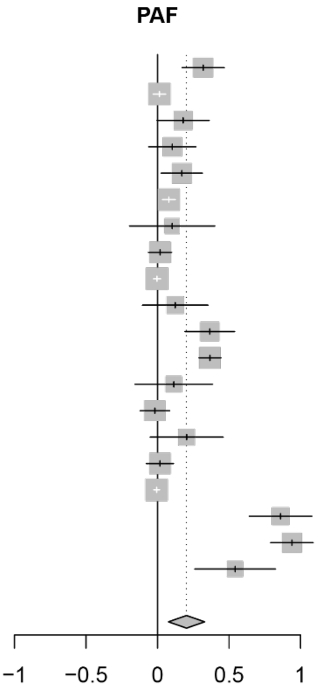

Abbreviations: HBsAg, Hepatitis B surface antigen; Prev., prevalence; HCC, Hepatocellular carcinoma; RR, risk ratio; PAF, population attributable fraction; CI, confidence interval (normal approximation using standard error calculated via parametric boostrap); SE standard error (calculated via parametric bootstrap); Rep., Republic.
